# Transcriptome adaptation of the bovine mammary gland to diets rich in unsaturated fatty acids shows greater impact of linseed oil over safflower oil on gene expression and metabolic pathways

**DOI:** 10.1186/s12864-016-2423-x

**Published:** 2016-02-09

**Authors:** Eveline M. Ibeagha-Awemu, Ran Li, Adolf A. Ammah, Pier-Luc Dudemaine, Nathalie Bissonnette, Chaouki Benchaar, Xin Zhao

**Affiliations:** Agriculture and Agri-Food Canada, Research and Development Centre, Sherbrooke, Quebec J1M 0C8 Canada; Department of Animal Science, McGill University, Ste-Anne-de-Bellevue, Quebec H9X 3 V9 Canada

**Keywords:** Transcriptome, Mammary gland, Linseed oil, Safflower oil, Fatty acid synthesis, Lipid synthesis, Lipid metabolism, Canadian Holstein cows

## Abstract

**Background:**

Nutritional strategies can decrease saturated fatty acids (SFAs) and increase health beneficial fatty acids (FAs) in bovine milk. The pathways/genes involved in these processes are not properly defined. Next-generation RNA-sequencing was used to investigate the bovine mammary gland transcriptome following supplemental feeding with 5 % linseed oil (LSO) or 5 % safflower oil (SFO). Holstein cows in mid-lactation were fed a control diet for 28 days (control period) followed by supplementation with 5 % LSO (12 cows) or 5 % SFO (12 cows) for 28 days (treatment period). Milk and mammary gland biopsies were sampled on days-14 (control period), +7 and +28 (treatment period). Milk was used to measure fat(FP)/protein(PP) percentages and individual FAs while RNA was subjected to sequencing.

**Results:**

Milk FP was decreased by 30.38 % (LSO) or 32.42 % (SFO) while PP was unaffected (LSO) or increased (SFO). Several beneficial FAs were increased by LSO (C18:1n11t, CLA:10t12c, CLA:9c11t, C20:3n3, C20:5n3, C22:5n3) and SFO (C18:1n11t, CLA:10t12c , C20:1c11, C20:2, C20:3n3) while several SFAs (C4:0, C6:0, C8:0, C14:0, C16:0, C17:0, C24:0) were decreased by both treatments (*P* < 0.05). 1006 (460 up- and 546 down-regulated) and 199 (127 up- and 72 down-regulated) genes were significantly differentially regulated (DE) by LSO and SFO, respectively. Top regulated genes (≥2 fold change) by both treatments (*FBP2, UCP2, TIEG2*, *ANGPTL4, ALDH1L2*) are potential candidate genes for milk fat traits. Involvement of *SCP2, PDK4, NQO1, F2RL1, DBI, CPT1A, CNTFR, CALB1, ACADVL, SPTLC3, PIK3CG, PIGZ, ADORA2B*, *TRIB3, HPGD, IGFBP2* and *TXN* in FA/lipid metabolism in dairy cows is being reported for the first time. Functional analysis indicated similar and different top enriched functions for DE genes. DE genes were predicted to significantly decrease synthesis of FA/lipid by both treatments and FA metabolism by LSO. Top canonical pathways associated with DE genes of both treatments might be involved in lipid/cholesterol metabolism.

**Conclusion:**

This study shows that rich α-linolenic acid LSO has a greater impact on mammary gland transcriptome by affecting more genes, pathways and processes as compared to SFO, rich in linoleic acid. Our study suggest that decrease in milk SFAs was due to down-regulation of genes in the FA/lipid synthesis and lipid metabolism pathways while increase in PUFAs was due to increased availability of ruminal biohydrogenation metabolites that were up taken and incorporated into milk or used as substrate for the synthesis of PUFAs.

**Electronic supplementary material:**

The online version of this article (doi:10.1186/s12864-016-2423-x) contains supplementary material, which is available to authorized users.

## Background

Cow milk provides nutritional benefits to humans and over the years, intensive efforts have been geared towards increasing milk production as well as its beneficial components. Growing awareness of the association between diet and health has driven market demands towards healthier products. Consequently, increasing the concentration of milk beneficial fatty acids (FAs) with protective roles against several human diseases including cancer, cardiovascular diseases, and general health of the gastrointestinal tract and immunity [1–3] is of great interest. Several strategies including nutrition and genome variation are under exploration to achieve increased milk beneficial components.

Nutritional strategies include feeding high grain/low forage and feed supplements rich in unsaturated fatty acids (USFAs) (e.g. soybean oil, corn oil, safflower oil, linseed oil, canola oil, marine algae, and fish oil) to dairy animals. These strategies have shown reduced yields of milk fat and milk saturated fatty acids (SFAs) of all chain lengths [4, 5]. In particular, FAs synthesized *de novo* are decreased to a greater extent, and this results in a shift in milk FA profile such that the proportion of short and medium-chain FAs are decreased and longer-chain and USFAs are increased [4, 5]. Thus, milk fat synthesis under regulation by dietary factors and feed ingredients high in USFAs cause a phenomenon known as milk fat depression whereby milk fat yield can be reduced by up to 50 % while other milk components are unaffected [6, 7].

Linseed oil is an attractive supplement in cow’s diets due to its rich content of alpha-linolenic acid (ALA, C18:3n3), about 50 to 60 % of total fat. Dietary supplementation of cows’ diets with different forms of linseed, including linseed oil has resulted to increases in ALA, conjugated linoleic acid (CLA) and other omega 3 FAs content of milk [8, 9]. Oil from linoleic acid (LA) rich safflower (about 76 % of total fat) variety is also used in ruminant diets. Safflower oil supplementation has resulted to increases in the CLA content of milk in dairy cows [8] and in lean/muscle tissues of lamb [10, 11]. ALA is a polyunsaturated fatty acid (PUFA) with three double bonds while LA, also a PUFA has two double bonds. The different degrees of unsaturation of the oils may result in different intermediates of biohydrogenation which may affect differently, the pathways of lipid metabolism. The profile of ruminal biohydrogenation intermediates is dependent on the composition of the diet [12] and both *in vivo* and *in vitro* studies have reported the formation of a wide range of C18:1, C18:2, C18:3 and CLA intermediates during biohydrogenation [12, 13]. Dietary supplementation of ruminant diets with plant and fish oils has been shown to affect feed intake and nutrient digestibility [14, 15]. While addition of 3 % LSO (on a dry matter basis-DM) to the diets of dairy cows showed no negative effects [14], Martin *et al*. [15] observed decreased dry matter intake and nutrient digestibility following addition of 5.7 % LSO to the diet. Recently, it was shown that up to 4 % LSO can be added to the diet of dairy cows without negative effects [13].

Numerous studies have considered the possible molecular mechanisms underlying diet-induced changes in mammary lipogenesis and fat secretion [16–18]. It has been proposed that intermediates (e.g. *trans*-10,*cis*-12-CLA) from alternative ruminal biohydrogenation pathways of USFAs exhibit inhibitory effects on milk fat synthesis [19–22] and milk yield [6]. Results of investigations into the molecular basis of nutrient effect on milk fat content and composition revealed a mechanism of down regulation of well-established lipogenic genes in mammary tissue [22–25]. Furthermore, down regulation of the mRNA abundance of genes encoding enzymes involved in FA transport/uptake, *de novo* FA synthesis, desaturation of FA, and triglyceride synthesis has been associated with milk fat depression [20]. Other studies have provided further insights into the network of genes and transcription regulators driving milk fat synthesis [26–30]. Several transcription factors including sterol regulatory binding protein 1 (SREBP1) have been implicated in the down regulation of gene expression during the processes of milk fat depression [22, 31, 32]. However, these candidate gene approaches consider only a few genes at a time thus limiting the breadth of information that can be uncovered. One study [30] has used a global microarray approach to study the transcriptome response of the bovine mammary gland to diets rich in USFAs and uncovered 972 differentially regulated genes between untreated and treated (diets supplemented with 2.7 rapeseed oil, 2.7 soybean oil and 2.7 % linseed oil, and a 1:1:1 mixture of the three oils) samples of the same cows and also uncovered several gene pathways that may be involved. This study [30] informed on the necessity of using a higher power integrative genomics strategy with possibility to interrogate the entire transcriptome without restrictions imposed by chip based assays (e.g. microarray) to better understand the molecular mechanisms underlying the effect of nutrition on milk fat synthesis and lipogenesis in general.

The next-generation RNA sequencing technology (RNA-Seq) allows the whole transcriptome characterization of the expression of all the genes that are expressed under a given condition thereby providing deeper knowledge of the transcriptomic events under such conditions. This approach has been successfully used to study the bovine milk/mammary tissue transcriptome [33–37] but none of these studies considered the effect of USFAs on mammary gland transcriptome. In this study, we used RNA-Seq to gain unprecedented insights into the regulatory mechanisms underlying nutrient effect on milk fat synthesis and concomitant increase in milk USFAs of benefit to human health.

## Methods

### Animals, management and sampling

The study was conducted in the animal facility of the Dairy and Swine Research and Development Centre, Agriculture and Agri-Food Canada. Animal care, management and use procedures were approved by the Animal Care and Ethics Committee of Agriculture and Agri-Food Canada, according to the Canadian Council on Animal Care [38].

Procedures for animal management have been reported in our companion paper on the same animals [39]. Briefly, twenty-four Canadian Holstein cows, producing 35 ± 10 kg of milk per day and in mid lactation (150 ± 50 days in milk) were used. Animals were grouped according to number of days in milk and randomly allocated to two treatments in a completely randomized block design. Twenty-four cows were fed a control diet composed of a total mixed ration of corn and grass silages (50:50) and concentrates (on dry matter bases-DM) for 28 days. This was followed by a treatment period of 28 days during which 12 animals received the control diet supplemented with 5 % linseed oil (LSO) on DM basis (LSO treatment) while another group of 12 animals were fed the control diet supplemented with 5 % safflower oil (SFO) on DM basis (SFO treatment). The FA content of LSO is comprised of 50 to 60 % (g/100 g) α-linolenic acid (C18:3n-3) while SFO is made up of about 76 % (g/100 g) linoleic acid (C18:2n6cc). Ingredients and composition of experimental diets are shown in Additional file 1. Animals were fed individually and had ad libitum access to water throughout the experiment. Feed intake was monitored daily and body weights were taken at the end of each period. Milk samples were collected on day-14 (control period), day+7 (7 days after onset of treatment), and day+28 (last day of treatment) for the measurement of test day fat percentage (FP), protein percentage (PP) and FA profiles. Mammary gland biopsies (performed on six animals/treatment) were performed as previously described [40] four hours after the morning milking on day-14, day+7 and day+28. Biopsies were performed at approximately the same site and same quarter each time. Tissues were snap frozen in liquid nitrogen and stored at −80 °C until used.

### Determination of test day fat percentage (FP), protein percentage (PP) and fatty acid profiles

FP and PP were measured in milk samples with MilkoScan FT 6000 Series mid-range infrared Fourier transform infrared-based spectrometers (Foss, Hillerod, Denmark) by Valacta, the Dairy Production Centre of Expertise for Quebec and the Atlantic provinces (Valacta Laboratories Inc., Ste-Anne-de-Bellevue, QC, Canada, www.valacta.com). Individual FA profiles were determined by gas chromatography according to O’Fallon *et al*. [41]. The Hewlett Packard 6890 N gas chromatographic system (Agilent Technology, Wilmington, DE, USA) equipped with a flame ionization detector and an auto sampler (Hewlett Packard, Avondale, PA, USA) and the gas chromatographic capillary column SLB-IL111 (100 m × 0.25 mm, 0.2 μm in thickness, Supelco, Bellefonte, PA, USA) were used. Hydrogen was the gas carrier at 1 mL/min constant flow with linear velocity of 26 cm/s. Oven temperature was set at 40 °C for 1 min, followed by 80 °C to 170 °C for 1 min, 40 °C to 195 °C for 2 min and 20 °C to 210 °C for 15 min. Injection port and detector temperatures were set at 250 °C, split ratio was set to 1:100 and injection volume was 1 μl. Quantification of FAs was performed with Agilent Technologies Chemstation vB.04.03 software using the FA methyl ester standard GLC No. 463 (Nu-Chek Prep Inc., Elysian, MN, USA) and C13:0 as the internal standard.

### Statistical analysis

Effect of treatments on test day milk FP, PP and individual FAs was analyzed with SAS software v9.3 (SAS Institute Inc., Cary, NC, USA). A completely randomized design with repeated measures and mixed effects ANOVA model (F-test) with Tukey adjustment was used. Multiple comparisons were applied to data collected on day-14 (control period), day+7 (7 days after onset of treatments) and day+28 (last day of treatment). Data was asymmetrically distributed for some FAs and was log transformed to fit a normal distribution before applying the ANOVA model. The least square means of the log transformed data were back transformed with a 95 % confidence interval for result interpretation.

### RNA isolation and sequencing

Total RNA from mammary gland biopsies (50 mg/sample) were purified using miRNeasy Kit (Qiagen Inc., Toronto, ON, Canada) according to manufacturer’s instructions, followed by DNase digestion of genomic DNA with Turbo DNase Kit (Ambion Inc. Foster City, CA, USA). The concentration of RNA was measured with Nanodrop ND-1000 instrument (NanoDrop Technologies, Wilmington, DE, USA) and the quality was assessed with Agilent 2100 Bioanalyzer (Agilent Technologies, Santa Clara, CA, USA) using the RNA 6000 Nano Labchip Kit (Agilent Technologies). All 36 samples had a RNA Integrity Number value greater than eight.

Libraries were generated from 250 ng of total RNA using the TruSeq stranded mRNA Sample Preparation Kit (Illumina Inc. San Diego, CA, USA), as per the manufacturer’s recommendations. Libraries were quantified using the Quant-iT™ PicoGreen® dsDNA Assay Kit (Life Technologies, Burlington, ON, Canada) and the Kapa Illumina GA with the Revised Primers-SYBR Fast Universal Kit (D-Mark Biosciences, Toronto, ON, Canada). Average size fragment was determined using a 2100 Bioanalyzer (Agilent Technologies) instrument. Cluster formation on the flow cell was performed using the cBot instrument (Illumina Inc,). The 36 libraries were multiplexed in equal ratios (six/lane) and sequenced on six lanes in the form of 50-cycle 100 bp paired-end reads, on a HiSeq 2000 system (Illumina Inc.) running HCS software v2.2.58. After sequencing, demultiplexed FASTQ files were generated by allowing up to one mismatch in the index. Library preparation and sequencing were performed by McGill University and Genome Quebec Innovation Centre (MUGQIC, http://gqinnovationcenter.com/).

A RNA-Seq analysis pipeline developed by MUGQIC was used for adaptor trimming, genome mapping, and transcript assembly. Briefly, trimming and clipping was done with Trimmomatic software v0.32 [42]. Reads with a minimum length of 32 nucleotides were aligned to the UMD3.1 Bos taurus reference genome [43] with TopHat v2.0.11 and then assembled into transcripts using Cufflinks v2.2.1 with genome annotation gtf file from Ensembl release 77 [44]. HTSeq-count script from the HT-Seq software suite [45] was used to generate raw read counts from the assembled transcripts. The trimmed mean of Mvalues (TMM) normalization method was used to normalize read counts data while reads per kilo base per million mapped reads (RPKM) was used to establish the transcript expression rate. EdgeR v3.10 software [46] was used to identify differentially expressed genes [47]. Because a small number of genes are highly expressed in the mammary gland and could hamper accurate quantification of lowly expressed genes, differentially expressed genes were defined significant as having a false discovery rate (FDR) with a Benjamini and Hochberg [48] corrected *P* value < 0.1 as recommended [49].

### Functional annotation and pathway analyses of differentially expressed genes

Functional annotation and pathway analyses of differentially expressed genes were performed using Ingenuity Pathways Analysis (IPA) software, March 2015 release (http://www.ingenuity.com/, Qiagen, Redwood City, CA, USA). Within IPA, the ‘Core Analysis’ function was employed to unlock the biological meaning of genes that were differentially expressed between treatments and controls in the context of canonical pathways, disease and biological functions, and networks.

### Real-time qPCR verification of RNA sequencing results

The expression levels of ten genes, including nine differentially expressed (*ACE2, FASN, FBP2, MROH2B, RASD1, SREBF1, TC2N, TIEG2,* and *UCP2*) genes and one highly expressed gene (*CSN2*) were analyzed using real-time quantitative PCR. qPCR was performed as a validation of RNA sequencing results. Reverse transcription was performed with the SuperScript™ II Reverse Transcriptase (Life Technologies Inc., Burlington, ON, Canada), using aliquots (1 μg) of the same total RNA used in RNA sequencing analysis. The cDNA samples were diluted based on the concentration of starting RNA to 20 ng/μl. Gene-specific primers were designed using Integrated DNA Technologies RealTime qPCR Assay tool (https://www.idtdna.com/scitools/Applications/RealTimePCR/) (Additional file 2). PCR efficiencies measured for all primer pairs were all within a range of ±10 %. The PCR reaction mix was composed of 5 μL Power SYBR® Green PCR Master Mix (Life Technologies Inc., Burlington, ON, Canada), 3 μL cDNA, 300 to 900 nM of each forward and reverse primers (Additional file 2), and 0.1 U AmpErase® Uracil N-Glycosylase (UNG, Life Technologies). All real-time PCR reactions were performed using the StepOnePlus™ Real-Time PCR System (Life Technologies) and the amplifications were done using the Power SYBR® Green PCR Master Mix (Life Technologies). The thermal cycling conditions were composed of a step for UNG treatment at 25 °C for 5 min followed by an initial denaturation/activation step at 95 °C for 10 min, 40 cycles at 95 °C for 30s, 60 °C for 30s and 72 °C for 30s. The experiments were carried out in triplicate for each data point. The relative quantification of gene expression was determined using the 2^-ΔΔCt^ method [50]. The fold change in gene expression was obtained following normalization to two reference genes, *RPS15* and *GAPDH*. The two reference genes were found to be the most stable out of four genes (*RPS15, GAPDH, UXT*, and *RPS9*) tested by Normfinder [51]. A pool of all the samples was used as inter-plate calibrator.

## Results

### Effect of treatments on feed intake and body weights of animals

Weekly feed intake decreased significantly (*P* < 0.05) during dietary supplementation with LSO (49.53 ± 1.28 kg [week four of treatment]) and SFO (47.89 ± 1.58 kg [week four of treatment]) as compared to the control period (55.88 ± 2.04 kg [week two of control period] for LSO and 55.67 ± 2.16 kg [week two of control period] for SFO). Body weight of animals increased significantly (*P* = 0.018) during LSO supplementation (633.67 ± 17.90 kg) as compared to the control period (618.33 ± 15.01 kg) while SFO supplementation had no significant effect on body weight of cows (643 ± 6.8 kg [treatment period] as compared to 637.67 ± 4.04 kg [control period]).

### Effect of treatments on milk fat/protein percentages, and individual fatty acid profiles

LSO treatment decreased by 30.38 % milk FP (*P* < 0.0001) from 3.62 ± 0.15 % (day-14) to 2.52 ± 0.15 % (day+28) while PP was unaffected, 3.37 ± 0.09 % (day-14) vs 3.36 ± 0.12 % (day+28) (Table 1). Similarly, SFO treatment decreased (*P* < 0.0001) FP by 32.42 % from 3.69 ± 0.09 % (day-14) to 2.50 ± 0.10 % (day+28). In contrast to LSO treatment, PP was increased (*P* < 0.0001) by SFO supplementation (3.69 ± 0.09 [day+28] vs 3.42 ± 0.07 [day-14]) (Table 1).

**Table 1:** Effect of supplemental feeding with 5 % linseed oil or 5 % safflower oil on test day milk fat percentage, protein percentage and individual fatty acid profiles (g/100 g) of Canadian Holstein cows

Parameter		Linseed oil treatment			Overall	Safflower oil treatment			Overall
		Day-14	day+7	day+28	*P*-value	Day-14	day+7	day+28	*P*-value
Fat percent		3.621^c^ ± 0.146	3.51^c^ ± 0.150	2.521^b^ ± 0.150	0.0001	3.692^d^ ± 0.094	3.423^c^ ± 0.101	2.495^b^ ± 0.100	0.0001
Protein percent		3.367^c^ ± 0.089	3.286^b^ ± 0.091	3.364^bc^ ± 0.121	0.195	3.420^c^ ± 0.074	3.306^b^ ± 0.064	3.685^c^ ± 0.091	0.0001
^a^Fatty acid									
	^e^C4:0	2.476 ± 0.244^b^	0.787 ± 0.244^c^	0.986 ± 0.244^c^	0.0000	2.276 ± 0.234^b^	1.261 ± 0.234^c^	0.894 ± 0.243^c^	0.0013
	C6:0	1.930 ± 0.200^b^	1.647 ± 0.200^b^	0.753 ± 0.200^c^	0.0003	2.202 ± 0.193^b^	1.844 ± 0.193^b^	0.722 ± 0.199^c^	0.0000
	C8:0	0.071 [0.049-0.105]^b^	0.039 [0.027- 0.058]^c^	0.020 [0.013-0.031]^c^	0.0000	0.077 [0.054-0.109]^b^	0.041 [0.029-0.058]^bc^	0.028 [0.017-0.048]^c^	0.0239
	C11:0	0.026 [0.012-0.058]	0.024 [0.011-0.053]	0.015 [0.007-0.034]	0.4416	0.037 [0.017-0.077]	0.017 [0.008-0.036]	0.032 [0.014- 0.076]	0.2575
	C12:0	0.184 ± 0.362^b^	1.572 ± 0.362^b^	1.175 ± 0.362^b^	0.0037	1.454 ± 0.347	1.408 ± 0.347	1.060 ± 0.361	0.7419
	C13:0	0.035[0.019-0.065]	0.029 [0.015-0.054]	0.014 [0.008-0.026]	0.0795	0.051 [0.029-0.092]^b^	0.022 [0.012-0.039]^bc^	0.008 [0.005-0.015]^c^	0.0009
	C14:1 t	0.023[0.014- 0.035]^b^	0.014 [0.009-0.021]^bc^	0.010 [0.007- 0.016]^c^	0.0288	0.024 [0.016-0.036]	0.015 [0.010-0.023]	0.013 [0.008-0.020]	0.1071
	C14:1	1.166[0.508-2.677]^b^	0.266 [0.120-0.591]^c^	0.109 [0.047-0.250]^c^	0.0009	1.086 [0.504-2.341]^b^	0.274 [0.127- 0.590]^c^	0.090 [0.039- 0.205]^d^	0.0000
	C14:0	13.161 ± 1.646^b^	8.004 ± 1.646^c^	5.726 ± 1.646^c^	0.0060	13.626 ± 1.581^b^	9.572 ± 1.581^c^	5.900 ± 1.633^d^	0.0016
	C15:0	0.468[0.175-1.250]^b^	0.025 [0.009-0.066]^c^	0.018 [0.007-0.049]^c^	0.0000	0.427 [0.166-1.099]^b^	0.017 [0.007-0.044]^c^	0.016 [0.006-0.042]^c^	0.0001
	C16:0	34.439 ± 5.085^b^	14.653 ± 5.085^c^	8.502 ± 5.085^c^	0.0022	31.419 ± 4.886^b^	13.141 ± 4.886^c^	15.542 ± 5.082^c^	0.0301
	C17:0	2.809 ± 0.446^b^	1.643 ± 0.446^c^	1.157 ± 0.446^c^	0.0108	2.585 ± 0.428^b^	1.792 ± 0.428^bc^	1.018 ± 0.443^c^	0.0555
	C18:0	4.080 ± 1.930^c^	11.540 ± 1.930^b^	3.491 ± 1.930^c^	0.0040	5.430 ± 1.855^c^	15.916 ± 1.855^b^	5.967 ± 1.926^c^	0.0000
	C18:1n9t	0.191 ± 0.027^bc^	0.216 ± 0.027^b^	0.147 ± 0.027^c^	0.0132	0.121 ± 0.026	0.200 ± 0.026	0.136 ± 0.027	0.0394
	C18:1n9c	10.940 ± 3.450	13.174 ± 3.450	14.442 ± 3.450	0.7999	8.903 ± 3.314^c^	21.429 ± 3.314^b^	12.595 ± 3.446^bc^	0.0025
	C18:1n11t	0.023 [0.007-0.074]^c^	0.160 [0.049-0.519]^b^	0.175 [0.054-0.567]^b^	0.0132	0.028 [0.009- 0.088]^c^	0.087 [0.028-0.269]^bc^	0.391 [0.121- 1.269]^b^	0.0074
	C18:2n6cc	0.064[0.019-0.215]^b^	0.186 [0.055-0.626]^c^	0.051 [0.015-0.172]^b^	0.0000	0.101 [0.032-0.325]^b^	0.281 [0.087-0.901]^c^	0.069 [0.021-0.232]^d^	0.0000
	C18:2n6tt	0.172 [0.094-0.316]	0.018 [0.010-0.031]	0.030 [0.017-0.054]	0.1878	0.219 [0.125-0.383]	0.083 [0.047- 0.145]	0.028 [0.016-0.051]	0.1892
	C18:3n3	0.068 [0.025-0.183]	0.164 [0.061-0.442]	0.249 [0.092-0.672]	0.2115	0.070 [0.027-0.181]	0.115 [0.044-0.299]	0.190 [0.070-0.512]	0.2577
	CLA:9c11t	0.047 [0.022-0.068]^c^	0.053 [0.036-0.076]^bc^	0.069 [0.047-0.100]^b^	0.3582	0.045 [0.022-0.093]	0.054 [0.026-0.113 ]	0.073 [0.034- 0.156]	0.7682
	CLA:10t12c	0.014 [0.008-0.023]^c^	0.071 [0.044-0.120]^b^	0.047 [0.030-0.075]^b^	0.0129	0.020 [0.014-0.029]^c^	0.034 [0.022-0.053 ]^b^	0.038 [0.024-0.062]^c^	0.3535
	C20:0	0.013[0.008- 0.021]	0.022 [0.013-0.036]	0.021 [0.013- 0.035]	0.2941	0.014 [0.008-0.022]	0.010 [0.006-0.017]	0.013[0.008- 0.022]	0.5565
	C20:1C5	0.067 [0.024-0.183]	0.019 [0.007- 0.051]	0.020 [0.008-0.053]	0.0524	0.061 [0.024- 0.154]	0.038 [0.015- 0.101]	0.014 [0.005-0.036]	0.0880
	C20:1C8	0.011 [0.005-0.027]	0.078 [0.033-0.187]	0.104 [0.042-0.258]	0.0053	0.027 [0.012-0.062]	0.008 [0.004-0.019]	0.018 [0.008-0.044]	0.1279
	C20:1C11	0.014 [0.008-0.025]	0.030 [0.017- 0.053]	0.027 [0.015-0.048]	0.1911	0.011 [0.006-0.020]^c^	0.038 [0.022-0.067]^b^	0.034 [0.019-0.061]^b^	0.0001
	C20:2	0.003 [0.002-0.005]	0.003 [0.002-0.005]	0.003 [0.002-0.005]	0.8993	0.003 [0.002-0.005]^c^	0.005 [0.003-0.008]^b^	0.014 [0.008-0.024]^b^	0.0047
	C20:5n3	0.008[0.005-0.013]^c^	0.016 [0.009- 0.026]^bc^	0.020 [0.012-0.034]^b^	0.0144	0.007 [0.004-0.011]	0.006 [0.004-0.010]	0.012 [0.007- 0.021]	0.0895
	C20:3n3	0.004[0.002-0.008]^c^	0.014 [0.008-0.024]^b^	0.021 [0.012-0.038]^b^	0.0003	0.004 [0.002-0.007]^c^	0.006 [0.003-0.010]^c^	0.020 [0.012-0.035]^b^	0.0017
	C22:5n3	0.004 [0.002-0.009]^c^	0.009 [0.005-0.018]^bc^	0.014 [0.007-0.027]^b^	0.0507	0.007 [0.004-0.013]	0.006 [0.003-0.011]	0.007 [0.004-0.014]	0.6231
	C22:0	0.011[0.008-0.015]^b^	0.005 [0.004-0.008]^c^	0.006 [0.004-0.008]^c^	0.0096	0.009 [0.007- 0.012]	0.008 [0.006-0.011]	0.006 [0.004-0.008]	0.1160
	C22:5n6	0.020 [0.013-0.031]	0.011 [0.007-0.017]	0.016 [0.010-0.025]	0.1746	0.011 [0.007-0.016]	0.015 [0.010-0.023]	0.013 [0.008-0.020]	0.4620
	C22:6n3	0.038 [0.023-0.064]^b^	0.019 [0.012-0.029]^bc^	0.011 [0.007-0.017]^c^	0.0036	0.028 [0.019-0.042]	0.027 [0.018-0.041]	0.015 [0.009-0.024]	0.1126
	C23:0	0.041 [0.009-0.190 ]^c^	0.168 [0.038-0.739]^b^	0.044 [0.010-0.194]^bc^	0.0133	0.053 [0.013- 0.221]	0.110 [0.026-0.455]	0.037 [0.009- 0.163]	0.4339
	C24:0	0.028 ± 0.004^b^	0.014 ± 0.004^c^	0.019 ± 0.004^bc^	0.0117	0.030 ± 0.004^b^	0.023 ± 0.004^bc^	0.015 ± 0.004^c^	0.0139
	Total SFA	60.743 ± 7.772^b^	40.580 ± 7.772^bc^	22.162 ± 7.772^c^	0.0059	60.974 ± 7.467^b^	45.653 ± 7.467^bc^	32.264 ± 7.748^c^	0.0314
	Total MUFA	13.125 ± 3.510	14.189 ± 3.510	15.808 ± 3.510	0.8823	11.429 ± 3.372^c^	22.300 ± 3.372^b^	13.556 ± 3.505^bc^	0.0064
	Total PUFA	0.559 [0.327-0.958]^c^	0.768 [0.449-1.316]^bc^	1.385 [0.809-2.372]^b^	0.0049	0.709 [0.423-1.190]^c^	0.849 [0.730-2.135]^bc^	1.190 [1.306-3.672]^b^	0.0124

^a^The mean values of C4:0, C6:0, C12:0, C14:0, C16:0, C17:0, C18:0, C18:1n9c, C18:1n9t, C24:0, total SFA (saturated fatty acids) and total MUFA (monounsaturated fatty acids) are represented as MEAN ± SEM, while those of C8:0, C11:0, C13:0, C14:1, C14:1 t, C15:0, C18:1n11t, C18:2n6cc, C18:2n6tt, C18:3n3, C20:0, C20:3n3, C20:5n3, C22:5n3, C22:5n6, C22:6n3, CLA:10t12c and CLA:9c11t are represented as MEAN (which is back transformed from the mean of logged mean value) with 95 % confidential intervals. ^b-d^For each parameter and treatment, means within a row with different superscripts differ significantly (*P* < 0.05). ^e^Results of the individual fatty acids, C4:0, C6:0, C8:0, C14:0, C16:0, C17:0, C18:0, C14:1, C14:1 t C18:1n9c, C18:1n9t, C18:1n11t, CLA:10t12c, CLA:9c11t , C18:2n6cc, C18:2n6tt, C18:3n3, C20:3n3, C20:5n3, C22:5n3 and C22:5n6 are the same reported in Li et al. [39]

The proportions of several SFAs (C4:0, C6:0, C8:0, C14:0, C16:0, C17:0, C24:0 and total SFA) were decreased significantly following feeding with both supplements (day-14 vs day+28) while the contents of C12:0 was increased (*P* < 0.05) by LSO supplementation (Table 1). Interestingly, the content of C18:0 increased significantly (*P* < 0.01) one week after introduction of supplements as compared to day-14 and decreased thereafter. While the contents of several MUFAs were unaffected by treatments, the concentrations of C14:1 and C14:1 t were significantly decreased (*P* < 0.05) by LSO supplementation and C14:1 by SFO supplementation (Table 1). The content of C18:1n11t was increased (*P* < 0.05) after 28 days of feeding both supplements and C20:1c11 by SFO only. The composition of C18:1n9c was increased by both supplements but significant increase was recorded following one week of supplementation with SFO. For PUFAs, the concentrations of CLA:10t12c, CLA:9c11t, C20:3n3, C20:5n3 and C22:5n3 were significantly (*P* < 0.05) increased after four weeks of feeding LSO while CLA:10t12c, C20:2 and C20:3n3 were significantly increased by SFO (Table 1). Increases in C18:2n6cc (LSO and SFO) were only significant on day+7 as compared to day-14. The proportion of C18:3n3 increased during supplemental feeding of both supplements but not significantly. The contents of more PUFAs were unaffected by SFO (C18:2n6tt, C20:5n3, C22:5n3, C22:5n6 and C22:6n3) supplementation than by LSO (C18:2n6tt, C20:2 and C22:5n6) supplementation.

### Sequencing, mapping of transcripts and expressed genes

Thirty-six libraries generated on RNA samples from cows whose diets were supplemented with 5 % SFO or 5 % LSO were sequenced. RNA-sequencing generated a total of 1,178,749,318 (1.2 billion) raw paired reads with an average of 33 M reads per library. Alignment of reads showed that 87.10 % (1,026,544,221) mapped to the bovine genome (UMD3.1.77, has 24,617 annotated genes) (Additional file 3). Of the mapped reads, 96.40 % mapped to unique positions, 3.2 % mapped to multiple positions while 0.38 % were discordant alignments. Only reads that mapped to unique positions were used in further analysis. A total of 18,692 genes were expressed out of which, 11,151 gene transcripts with more than one count per million reads in at least 10 libraries [52] were used in differential gene expression (DE) analysis.

Using percentage of the total number of RPKM values, genes were classified into very highly (RPKM >2 % of total RPKM values), highly (0.1 to 1.99 %), medium (0.01 to 0.099 %), lowly (0.001 to 0.0099 %) and very lowly (<0.0099 %) expressed genes (Table 2). Twenty-four top expressed genes (very highly and highly expressed genes) and their functions are listed in Table 3. The expression of the six main milk protein genes, *CSN1S1*, *CSN1S2, CSN2, CSN3, LGB* and *LALBA*, and *GLYCAM1* was very high and all together constituted 79.45 % of the total RPKM values (Additional file 4a). Amongst the very highly expressed genes, *CSN2* and *CSN1S1* were the most expressed with RPKM values of respectively 23.94 and 20.05 % of total. The combined RPKM value of 17 highly expressed genes was 4.79 % of total (Additional file 4b). Medium expressed genes were about 200 while the rest, about 18400 were weakly abundant and therefore classified as lowly and very lowly expressed genes in our samples (Table 2).

**Table 2:** Classification of expressed genes according to level of expression

Category	^a^RPKM range (% of total)	Number of genes	
		Linseed oil treatment	Safflower oil treatment
Very highly expressed	>2 %	7	7
Highly expressed	0.1 to 1.99 %	15	17
Medium expressed	0.01 to 0.099 %	219	212
Lowly expressed	0.001 to 0.0099 %	2574	2522
^b^Very lowly expressed, retained	<0.0099 %	8508	8393
^c^Very lowly expressed, not used in DE	<0.0099 %	7369	7314

^a^Reads per kilo base per million mapped reads (RPKM)

^b,c^Very lowly expressed genes had RPKM values of <1 %. Those that had read counts >1CPM (counts per million) in at least 10 libraries were used in differential gene expression (DE) analysis while those with >1 CPM in less than 10 libraries were not used

**Table 3:** Twenty-four top expressed genes and their functions

Gene symbol	Gene name	^a^% RPKM abundance	Gene function
CSN2	Casein beta	23.939	Major milk protein, determine surface properties of casein micelles, source of bioactive peptides and amino acids, mammary gland specific protein
CSN1S1	Casein alpha s1	20.051	Major milk protein, antioxidant peptide, plays role in the transport of calcium phosphate, source of bioactive peptides and amino acids, mammary gland specific protein
LGB (PAEP)	Beta-lactoglobulin (progestagen-associated endometrial protein)	9.993	Major milk protein, play role in retinol binding
CSN1S2	Casein alpha-S2	9.197	Major milk protein, produce casocidin-I that inhibits the growth of bacteria, plays role in the transport of calcium phosphate, source of bioactive peptides and amino acids, mammary gland specific protein
CSN3	Casein kappa	8.658	Major milk protein, micelle formation to transport calcium, source of bioactive peptides (casoplatelin inhibits platelet aggregation, casoxins have opioid activity) and amino acids, mammary gland specific
LALBA	Lactalbumin, alpha-	5.484	Major milk protein, forms regulatory subunit of lactose synthase, enables lactose synthase to synthesize lactose, causes allergic reaction in some people, mammary gland specific
GLYCAM1	Glycosylation-dependent cell adhesion molecule 1	2.127	Highly and specifically expressed in the lactating mammary gland, play role in cell adhesion molecule binding
COX1	Cytochrome c oxidase subunit 1	0.937	Has catalytic activities, is a component of the respiratory chain that catalyzes the reduction of oxygen to water, subunits 1–3 form the functional core of the enzyme complex, belongs to the heme-copper respiratory oxidase family
COX3	Cytochrome c oxidase subunit 3	0.509	Has catalytic activity, subunits I, II and III form the functional core of the enzyme complex, belongs to the cytochrome c oxidase subunit 3 family
ATP6	ATP synthase FO subunit 6	0.430	Play role in the translocation of protons across the membrane, ATPase activity
MT-ND3	Mitochondrially encoded NADH dehydrogenase 3	0.344	Core subunit of the mitochondrial membrane respiratory chain NADH dehydrogenase (Complex I), is believed to belong to the minimal assembly required for catalysis. Complex I functions in the transfer of electrons from NADH to the respiratory chain
FABP3	Fatty acid binding protein 3, muscle and heart (mammary-derived growth inhibitor)	0.2921	Play a role in the intracellular transport of long-chain fatty acids and their acyl-CoA esters, belongs to calycin superfamily, fatty-acid binding protein family
ND1	NADH-ubiquinone oxidoreductase chain 1	0.298	Catalytic activity, NADH dehydrogenase activity, belongs to the complex I subunit 1 family
COII (MT-CO2)	Mitochondrially encoded cytochrome c oxidase II	0.301	Is the component of the respiratory chain that catalyzes the reduction of oxygen to water
MT-ATP8	Mitochondrially encoded ATP synthase 8	0.252	Unknown function. Is a player in metabolic pathways.
MT-CYB	mitochondrially encoded cytochrome b	0.248	Component of the ubiquinol-cytochrome c reductase complex (complex III or cytochrome b-c1 complex), a respiratory chain that generates an electrochemical potential coupled to ATP synthesis (by similarity)
HSTN	Histatherin	0.232	Unknown
SPP1	Secreted phosphoprotein 1	0.163	Acts as a cytokine, involved in enhancing production of interferon-gamma and interleukin-12, reduces production of interleukin-10, is essential in the pathway that leads to type I immunity, probably involved in cell adhesion, belongs to the osteopontin family
MT-ND4	Mitochondrially Encoded NADH Dehydrogenase 4	0.152	Core subunit of the mitochondrial membrane respiratory chain NADH dehydrogenase (Complex I), is believed to belong to the minimal assembly required for catalysis. Complex I functions in the transfer of electrons from NADH to the respiratory chain
RPLP1	Ribosomal protein, large, P1	0.144	Is a functional constituent of ribosomes and functions in translational elongation
MFGE8	Milk fat globule-EGF factor 8 protein	0.152	Specific ligand for alpha-v/beta-3 and alpha-v/beta-5 receptors, Contributes to phagocytic removal of apoptotic cells in many tissues
SCD	Stearoyl-CoA desaturase (delta-9-desaturase)	0.133	Catalyzes the insertion of a double bond into a spectrum of fatty acyl-CoA substrates including palmitoyl-CoA and stearoyl-CoA, belongs to the fatty acid desaturase family
RNASE1	Ribonuclease, RNase A family, 1	0.099	Nucleic acid binding and ribonuclease A activity
EEF1A1	Eukaryotic translation elongation factor 1 alpha 1	0.099	promotes the GTP-dependent binding of aminoacyl-tRNA to the A-site of ribosomes during protein biosynthesis, involved in Th1 (T helper 1) cytokine production

^a^RPKM, reads per kilo base per million mapped reads. Values presented as a percentage of all RPKM values

### Effect of supplemental linseed oil (LSO) feeding on mammary gland transcriptome

**Fig. 1:**
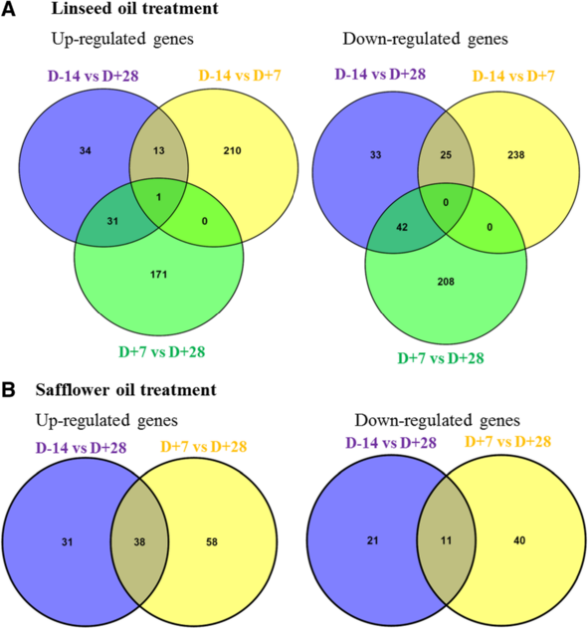
**a-b**: Number of commonly and uniquely regulated genes between the different periods of comparison: **a** Linseed oil treatment and **b** Safflower oil treatment

During the four weeks of supplementing the feed of cows with 5 % LSO following a control feeding period (no oil added to diet), transcriptome adaptations revealed that a total of 1006 genes were significantly differentially regulated (FDR < 0.1) including 460 up-regulated and 546 down regulated (Additional file 5a-c). Specifically, comparing gene expression of cows at 7 days (day+7) after onset of LSO supplementation with the control period (day-14) indicated that 224 genes were significantly up-regulated while 263 genes were significantly down regulated (Fig. 1a). When the control period (day-14) was compared with the late treatment period (day+28), fewer genes were affected, being 79 up-regulated while 100 were down regulated. Interestingly, the highest number of affected genes was seen when the early treatment period (day+7) was compared with the late treatment period (day+28), being 203 up- and 251 down-regulated genes. Similar and regulated genes unique to each pair of comparison are shown in Fig. 1a and Additional file 5d-e. Most significantly up-regulated genes with ≥2 fold increase in expression in at least one comparison period including *FBP2, DMGDH, UCP2, CYP2B6, TRIB3, SESN2, ANGPTL4, TIEG2, CPT1A, CALB1, ALDH1L2, RNASE1, KLK7* and *IGSF9B* are listed in Table 4. Similarly, down regulated genes with ≥2 fold decrease in expression including *M-SAA3.2, KRT15, CHI3L1, LPIN1, RAB17, MROH2B, SAA3, CYBRD1, LOXL4, CDH16, ASB11, C28H100R, LOXL4, LTF, CFI, SDS, NXPE2, FRMPD3* and *TMEM132E* are listed in Table 4.

**Table 4:** ^a^Differentially expressed genes with fold change ≥2 in mammary gland of cows fed 5 % linseed oil

	^b^LSO day+7 compared with day-14				LSO day+28 compared with day+7				LSO day+28 compared with day-14			
Gene	^c^FC	log2FC	*P*-value	FDR *P*-value	FC	log2FC	*P*-value	FDR *P*-value	FC	log2FC	*P*-value	FDR *P*-value
ASB11	−3.091	−1.628	1.3E-10	4.9E-07	2.102	1.072	2.2E-05	0.0039	2.102	1.072	2.2E-05	0.0039
DMGDH	−2.157	−1.109	0.0037	0.0930	4.359	2.124	6.0E-08	9.6E-05	4.359	2.124	6.0E-08	9.6E-05
ENSBTAG00000034372	−2.477	−1.309	8.4E-06	0.0026	2.000	0.987	0.0007	0.0361	1.982	0.987	0.0007	0.0361
FBP2	3.298	1.721	0.0026	0.0785	3.343	1.741	0.0005	0.0312	3.343	1.741	0.0005	0.0312
ESRP2	−2.000	−0.998	0.0001	0.0139	2.316	1.212	3.2E-06	0.0011	2.316	1.212	3.2E-06	0.0011
FRMPD3	2.198	1.136	4.0E-07	0.0003	−2.550	−1.351	1.8E-09	1.7E-05	−2.550	−1.351	1.8E-09	1.7E-05
IGSF9B	3.032	1.600	0.0010	0.0479	−2.708	−1.437	0.0031	0.0857	−2.708	−1.437	0.0031	0.0857
LTF	−2.452	−1.294	1.8E-05	0.0043	2.039	1.028	0.0005	0.0316	2.039	1.028	0.0005	0.0316
SMTNL2	−2.000	−0.969	9.0E-05	0.0128	2.151	1.105	6.1E-06	0.0015	2.151	1.105	6.1E-06	0.0014
RAB17	−2.537	−1.343	0.0005	0.0346	2.311	1.209	0.0017	0.0614	2.311	1.209	0.0017	0.0614
ALDH1L2	-	-	-	-	2.325	1.217	5.6E-06	0.0014	2.325	1.217	5.6E-06	0.0014
ANGPTL4	-	-	-	-	2.834	1.503	2.2E-06	0.0009	2.834	1.503	2.2E-06	0.0009
BEST3	-	-	-	-	−2.908	−1.540	3.1E-05	0.0052	−2.908	−1.540	3.1E-05	0.0052
BSP30C	-	-	-	-	−2.370	−1.245	5.3E-06	0.0014	−2.370	−1.245	5.3E-06	0.0014
C28H10ORF10	-	-	-	-	2.131	1.092	1.3E-06	0.0007	2.131	1.0917	1.3E-06	0.0007
CENPJ	-	-	-	-	−2.000	−1.000	0.0002	0.0142	−2.000	−1.000	0.0002	0.0142
CLSTN3	-	-	-	-	2.092	1.065	0.0012	0.0518	2.092	1.065	0.0012	0.0518
CYP2B6	-	-	-	-	3.123	1.643	7.8E-08	9.6E-05	3.123	1.643	7.8E-08	9.6E-05
ENSBTAG00000014220	-	-	-	-	2.355	1.236	9.2E-06	0.0020	2.355	1.236	9.2E-06	0.0020
ENSBTAG00000045737	-	-	-	-	3.366	1.751	0.0016	0.0598	3.366	1.751	0.0016	0.0598
FCAMR	-	-	-	-	2.081	1.057	0.0003	0.0219	2.081	1.057	0.0003	0.0219
IGFBP2	-	-	-	-	2.155	1.108	0.0019	0.0655	2.155	1.108	0.0019	0.0655
KRT18	-	-	-	-	2.083	1.059	5.6E-06	0.0014	2.083	1.059	5.6E-06	0.0014
NXPE2	-	-	-	-	−2.349	−1.232	0.0004	0.0280	−2.349	−1.232	0.0004	0.0280
RNASE1	-	-	-	-	2.021	1.015	0.0024	0.0736	2.021	1.015	0.0024	0.0735
SCN5A	-	-	-	-	−2.124	−1.087	0.0002	0.0171	−2.124	−1.087	0.0002	0.0171
SESN2	-	-	-	-	2.637	1.399	4.3E-07	0.0003	2.637	1.399	4.3E-07	0.0004
STMN1	-	-	-	-	2.064	1.046	2.1E-06	0.0009	2.064	1.046	2.1E-06	0.0009
TIEG2	-	-	-	-	2.015	1.011	2.8E-07	0.0003	2.015	1.011	2.8E-07	0.0003
TMEM132E	-	-	-	-	−2.951	−1.561	7.1E-05	0.0082	−2.951	−1.561	7.1E-05	0.0082
TRIB3	-	-	-	-	2.564	1.358	3.8E-07	0.0003	2.564	1.358	3.8E-07	0.0004
UCP2	-	-	-	-	3.153	1.657	7.2E-08	9.6E-05	3.153	1.657	7.2E-08	9.6E-05
ARG2	-	-	-	-	−1.951	−0.964	0.0002	0.0155	-	-	-	-
CALB1	2.240	1.164	0.0002	0.0170	-	-	-	-	-	-	-	-
CDH16	−2.818	−1.494	3.0E-15	3.4E-11	-	-	-	-	-	-	-	-
CDO1	−2.345	−1.230	5.3E-08	7.3E-05	-	-	-	-	-	-	-	-
CFB	−2.170	−1.118	0.0004	0.0326	-	-	-	-	-	-	-	-
CFI	−2.454	−1.295	0.0022	0.0737	-	-	-	-	-	-	-	-
CHI3L1	−4.095	−2.034	0.0009	0.0455	-	-	-	-	-	-	-	-
CYBRD1	−3.310	−1.727	7.4E-06	0.0024	-	-	-	-	-	-	-	-
ENSBTAG00000047368	−2.584	−1.370	0.0006	0.0372	-	-	-	-	-	-	-	-
ENSBTAG00000048058	−2.239	−1.163	3.6E-07	0.0004	-	-	-	-	-	-	-	-
FOS	−2.171	−1.118	2.2E-05	0.0049	-	-	-	-	-	-	-	-
H10ORF10	−2.497	−1.320	4.4E-09	1.0E-05	-	-	-	-	-	-	-	-
ITGB6	−2.296	−1.199	0.0039	0.0954	-	-	-	-	-	-	-	-
KRT15	−4.347	−2.120	5.8E-05	0.0099	-	-	-	-	-	-	-	-
LOXL4	−3.087	−1.626	1.4E-08	2.5E-05	-	-	-	-	-	-	-	-
MLXIPL	−2.125	−1.088	2.0E-06	0.0011	-	-	-	-	-	-	-	-
M-SAA3.2	−5.748	−2.523	9.0E-05	0.0128	-	-	-	-	-	-	-	-
PRR15L	−2.139	−1.097	0.0004	0.0315	-	-	-	-	-	-	-	-
SAA3	−3.834	−1.939	5.6E-06	0.0023	-	-	-	-	-	-	-	-
SDS	−2.355	−1.236	8.9E-05	0.0128	-	-	-	-	-	-	-	-

^a^Only genes with fold change values ≥2.00 are shown. Complete list of differentially expressed genes are shown in Additional file 5a-c. ^b^LSO- linseed oil, day-14 (control period), day+7 (7 days of supplemental feeding with 5 % linseed oil), day+28 (28 days of supplemental feeding with 5 % linseed oil). ^c^FC (Fold change)

### Effect of supplemental safflower oil (SFO) feeding on mammary gland transcriptome

SFO supplementation of the diets of mid-lactation cows had less effect on gene expression as compared to LSO. Results indicate that out of 11,151 genes compared, 199 genes were significantly differentially regulated (FDR < 0.1) (127 genes up-regulated and 72 down-regulated) by SFO during the 28 days of dietary supplementation (Fig. 1b and Additional file 6a-b). Comparing the different periods, no genes were significantly differential expressed between day-14 and day+7 while 69 genes were significantly up-regulated and 32 down-regulated when the late treatment period (day+28, four weeks after onset of treatment) was compared to the control period (day-14) (Fig. 1b and Additional file 6a-b). Similarly, 96 genes were up-regulated and 51 down-regulated when the late treatment period (day+28) was compared with the early treatment period (day+7) (Additional file 6a-b). Between these periods, 38 up-regulated and 11 down-regulated genes were common (Fig. 1b and Additional file 6c-d). The most significantly regulated gene was FBP2 (fructose-1,6-bisphosphatase 2) with a 6.3 fold increase in expression between day-14 and day+28. Genes with ≥ 2 fold changes in expression include *FBP2, ACE2, UCP2, OLRI*, ENSBTAG00000016127, *SLC17A9*, *RNASE1* and *SCIN* (up-regulated) and *MROH2B* and *ISG15* (down-regulated) and are shown in Table 5.

**Table 5:** ^a^Differentially expressed genes with fold change ≥2 in mammary gland of cows fed 5 % safflower oil (on dry matter bases)

	^b^SFO day+28 compared with day-14				SFO day+28 compared with day+7			
Genes	^c^FC	log2FC	*P*-value	FDR *P*-value	FC	log2FC	P-value	FDR *P*-value
RNASE1	3.664	1.874	2.33E-07	0.0008	3.280	1.713	7.21E-07	0.0018
UCP2	2.362	1.240	1.91E-06	0.0021	2.839	1.505	7.88E-09	8.79E-05
SPADH1	2.104	1.073	1.45E-05	0.0098	2.191	1.132	4.28E-06	0.0037
FBP2	6.302	2.656	2.52E-05	0.0111	4.973	2.314	4.26E-05	0.0127
LBP	2.318	1.213	2.87E-05	0.0122	2.052	1.037	0.0003	0.0437
DPP10	−2.308	−1.207	5.29E-05	0.0177	−2.110	−1.077	0.0004	0.0520
ANGPTL4	2.131	1.091	0.0001	0.0326	2.247	1.168	2.73E-05	0.0092
ACE2	3.061	1.614	1.28E-07	0.0008	-	-	-	-
HPGD	2.159	1.110	1.77E-07	0.0008	-	-	-	-
MROH2B	−2.784	−1.477	1.06E-05	0.0084	-	-	-	-
TIEG2	2.115	1.080	1.49E-05	0.0098	-	-	-	-
SCGB1D	2.112	1.079	4.70E-05	0.0168	-	-	-	-
OLR1	3.168	1.664	0.0004	0.0650	-	-	-	-
ALDH1L2	2.001	1.001	0.0004	0.0651	-	-	-	-
ENSBTAG00000016127	-	-	-	-	3.659	1.871	1.29E-06	0.0024
SLC17A9	-	-	-	-	2.543	1.346	4.08E-06	0.0037
MMP19	-	-	-	-	2.206	1.141	1.75E-05	0.0065
ENSBTAG00000000414	-	-	-	-	2.316	1.213	2.09E-05	0.0075
SCIN	-	-	-	-	2.510	1.328	2.31E-05	0.0081
IL17RB	-	-	-	-	2.217	1.149	4.57E-05	0.0127
STATH	-	-	-	-	−2.030	−1.022	0.0002	0.0393
ISG15	-	-	-	-	−2.676	−1.420	0.0006	0.0640
MX1					−1.964	−0.974	0.0009	0.0843

^a^Only genes with fold change values ≥2.00 are shown. Complete list of differentially expressed genes are shown in Additional file 6a-b. No genes were significantly differentially expressed between day+7 as compared to day-14. ^b^SFO (safflower oil), day-14 (control period), day+7 (7 days of supplemental feeding with 5 % safflower oil), day+28 (28 days of supplemental feeding with 5 % safflower oil). ^c^FC (Fold change)

Interestingly, both treatments significantly affected several genes in the solute-carrier superfamily of genes, 41 by LSO and 5 by SFO (Additional file 7). Whereas the effect of LSO on mammary gland transcriptome was more potent than SFO, no differences were observed when we compared the gene expression on day+7 of LSO and SFO treatments. The same observation was made when day+28 was compared. This was surprising since it was clear from gene expression results that LSO has a more pronounced effect on the transcriptome than SFO. We suspected that inter-cow variations could be responsible. To demonstrate this, we conducted a principal component analysis on the samples at the different time points (36 samples) and found out that neither component 1 and 2 or 2 and 3 could separate the animals according to treatment (Additional file 8). Instead, samples from the same animal at the different time points tended to cluster together. Also, animals were spread-out hinting at wide inter-animal variations.

### Real time qPCR validation of RNA-sequencing results

To validate gene expression results obtained by sequencing, nine DE genes and one highly (but not DE) expressed gene were selected and quantified by qPCR. Results are shown in Table 6 and support the differential expression pattern of genes obtained for both treatments by RNA-sequencing. Significant differential expression recorded by RNA-Seq for *ACE2, FABP2, CREBF1* and *UPS* was confirmed by qPCR. Significant differential expression of *TC2N* and *TIEG2* by RNA-Seq for LSO was confirmed by qPCR while their expression tended towards significance (*P* < 0.090) for SFO treatment by qPCR method. Expression of *FASN* by qPCR was significant in SFO treatment only. The expression of *MROH2B* and *RASD1* showed similar down-regulation patterns by both methods but only significant by RNA-seq method. *FBP2* expression level was highest by both methods of quantification.

**Table 6:** Results of qPCR validation of RNA-sequencing data

Gene	qPCR				RNA-seq		
	Fold change D-14 vs D7	Fold change D7 vs D28	Fold change D-14 vs D28	Overall *P*-value	Fold change D-14 vs D28	*P*-value	FDR *P*-value
Linseed oil treatment							
CSN2	1.011	−1.410	−1.397	0.3644	−1.003	0.9856	0.9956
ACE2	1.021	1.915**	1.955	0.0450	2.006**	1.85E-05	0.0050
FASN	−1.156	−1.167	−1.348	0.5653	−1.520*	0.0010	0.0740
FBP2	3.921**	3.248**	12.735**	0.0012	11.023**	1.95E-09	4.33E-06
MROH2B	−1.957	−1.506	−2.950	0.4434	−4.154**	0.0002	0.0229
RASD1	−1.376	−1.284	−1.767	0.2045	−1.719**	0.0004	0.0472
SREBF1	−1.934*	−1.080	−2.088**	0.0297	−1.645**	7.36E-07	0.0005
TC2N	1.124	1.998*	2.246**	0.0358	2.272**	1.82E-06	0.0009
TIEG2	1.069	2.382**	2.546**	0.0070	2.591**	8.01E-12	4.45E-08
UCP2	1.065	2.807**	2.989**	0.0019	2.256**	0.0002	0.0228
Safflower oil treatment							
CSN2	−1.233	−1.048	−1.292	0.5687	−1.003	0.9856	0.9956
ACE2	1.830*	1.551	2.838**	0.0241	3.061**	1.28E-07	0.0008
FASN	−1.420**	−1.318**	−1.873**	0.0020	−1.625*	0.0006	0.0854
FBP2	3.583*	3.980*	14.261**	0.0059	6.302**	2.52E-05	0.0111
MROH2B	−1.634	−1.972	−3.215*	0.1064	−2.784**	1.06E-05	0.0083
RASD1	1.162	−1.495	−1.287	0.1605	−1.878**	1.71E-06	0.0020
SREBF1	−1.859	−1.420	−2.646**	0.0123	−1.937**	4.62E-06	0.0042
TC2N	1.219	1.831	2.232*	0.0584	1.852*	0.0007	0.0946
TIEG2	1.187	1.547	1.837*	0.0903	2.114**	1.49E-05	0.0097
UCP2	1.002	1.948**	1.952	0.0475	2.362**	1.91E-06	0.0021

*FDR/*P*-value < 0.1 **FDR/*P*-value < 0.05

### Biological function enrichment and pathway analysis of DE genes

The effect of dietary SFO or LSO supplements on biological pathways was studied to identify functions and pathways that were most affected after four weeks of feeding diets high in USFAs to cows. Results show that these genes are enriched in functions related to molecular/cellular, diseases/disorders and physiological system, as well as canonical pathways and network functions (Additional files 9 and 10).

**Fig. 2:**
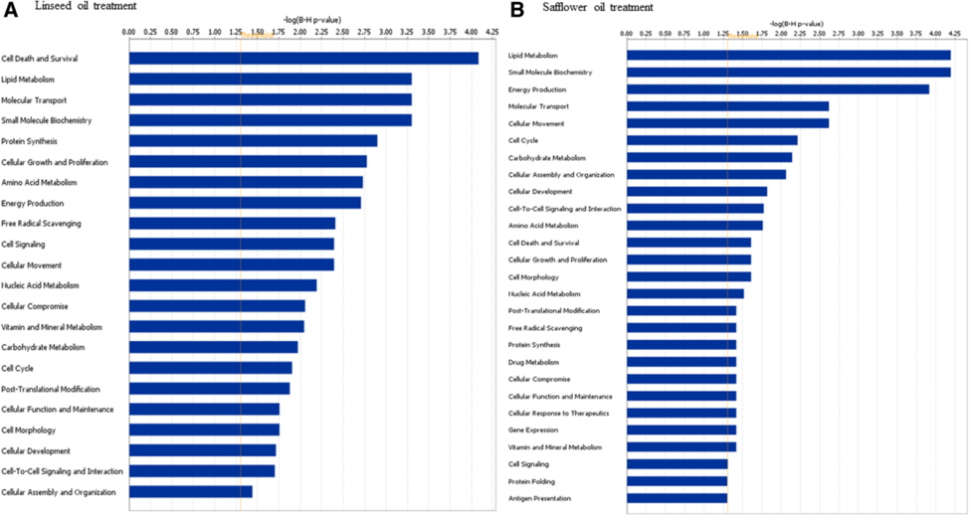
Differentially expressed genes in the mammary glands of cows on supplemental feeding with 5%linseed oil (**a**) or 5 % safflower oil (**b**) are significantly enriched in several molecular and cellular functions

Notable amongst enriched molecular and cellular functions for cows on LSO treatment were cell death and survival, lipid metabolism, molecular transport, small molecule biochemistry, protein synthesis, cellular growth and proliferation and amino acid metabolism (Fig. 2a). For safflower oil treatment, top cellular and molecular functions included lipid metabolism, small molecule biochemistry, energy production, molecular transport, cellular movement, cell cycle and carbohydrate metabolism (Fig. 2b). A striking observation concerning the affected pathways is the high similarity between the two treatments. Although a different ranking and involvement of more DE genes in LSO treatment was observed, most of the pathways affected by LFO were also affected by SFO (Fig. 2).

**Fig. 3:**
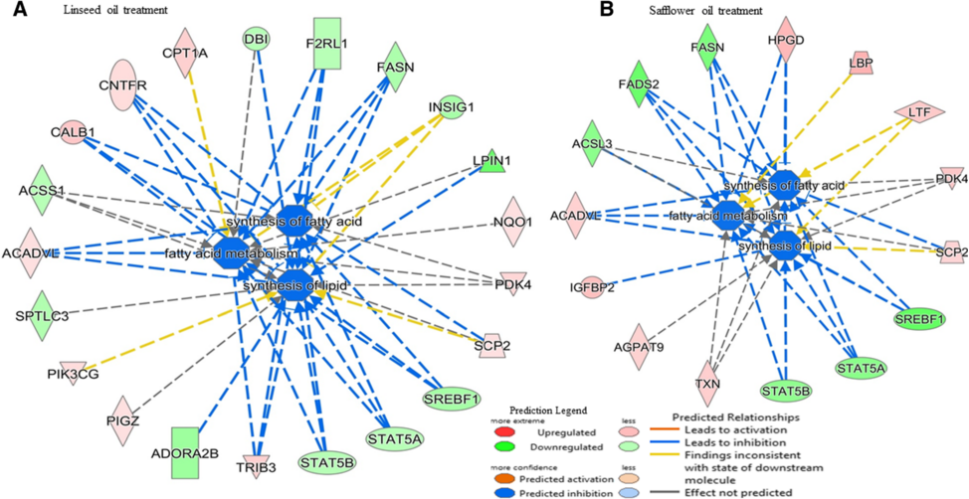
Interaction between differentially expressed genes predicted by IPA to significantly decrease synthesis of fatty acid (z-score = −2.70, *P*-value = 2.0E-05), synthesis of lipid (z-score = −2.281, *P*-value = 2.9E-04) and fatty acid metabolism (z-score = −2.212, *P*-value = 1.1E-04) in linseed oil treatment (**a**) and synthesis of fatty acid (z-score = −2.269, *P*-value = 3.9E-08) and lipid (z-score = −2.371, *P*-value = 4.3E-05, *P*-value = 1.17E-05) in safflower oil treatment (**b**). Activity of genes showed a negative z-score (−1.960) for fatty acid metabolism in safflower oil treatment but a decrease was not predicted by IPA

**Fig. 4:**
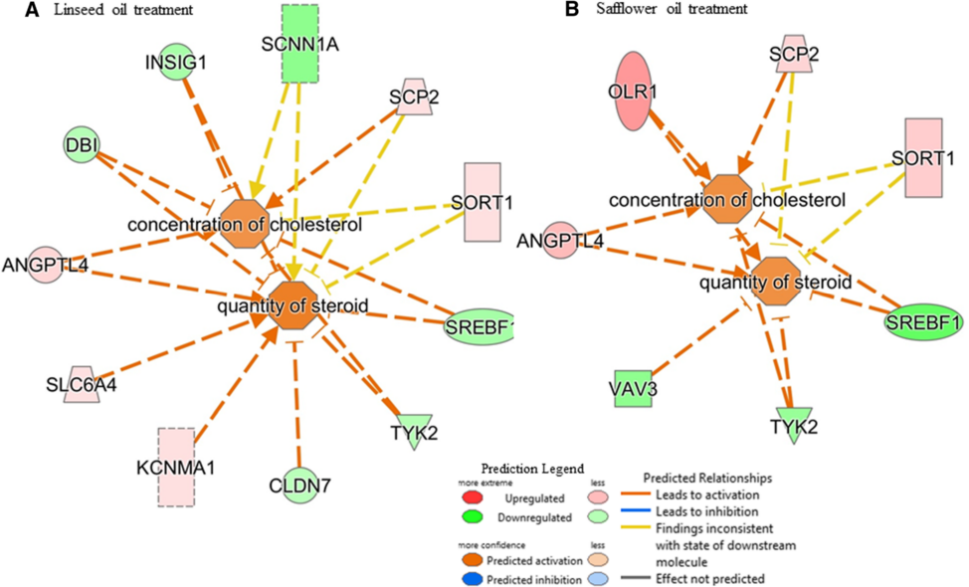
Interaction between differentially expressed genes in (**a**) linseed oil treatment (LSO) and (**b**) safflower oil treatment (SFO) showing increased activity in the concentration of cholesterol (z-score = 1.076, *P*-value = 7.68E-03 for LSO and 1.135 for SFO, *P*-value = 4.50E-03) and quantity of steroid (z-score = 1.428, *P*-value = 9.47E-03 for LSO and 1.129, *P*-value = 1.59E-03 for SFO)

**Fig. 5:**
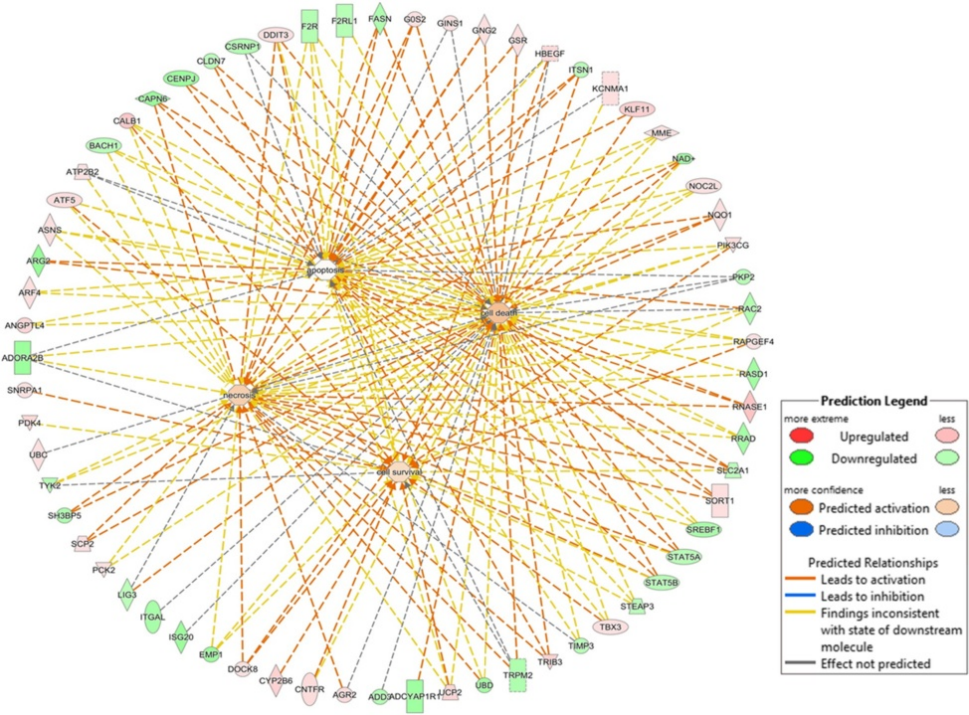
Interaction between differentially expressed genes implicated in apoptosis, necrosis, cell death and survival in cows whose diets were supplemented with 5 % linseed oil for 28 days as compared to the same cows on the control diet

Within the category of lipid metabolism, several functions were affected (Additional file 11a-b) and also more genes in LSO treatment (40 genes) were implicated as compared to SFO treatment (29 genes). Notable were the effects on concentrations of acylglycerol, triacylglycerol, lipid, FA and eicosanoid, oxidation of lipid and FA, synthesis of FA and lipid, accumulation of lipid and triacylglycerol, modification of palmitic acid, metabolism of FA and succinic acid, and conversion of lipid which according to a negative activation score (z-score) indicates a decrease in activity (Additional file 11a-b). In particular and considering the expression direction (up- or down-regulated) of implicated genes (Additional files 12, 13 and 14), IPA analysis predicts significant reduction of FA synthesis (z-score = −2.710, *P*-value = 2.0E-05), lipid synthesis (z-score = −2.281, *P*-value = 2.9E-04), and FA metabolism (z-score = −2.212, *P*-value = 1.1E-04) following supplemental feeding with LSO (Fig. 3a), and synthesis of FA (z-score = −2.269, *P*-value = 3.9E-08) and lipid (z-score = −2.371, *P*-value = 4.3E-05) following supplemental feeding with SFO (Fig. 3b). Whereas gene expression showed a negative z-score (−1.960, *P*-value = 1.17E-05) for FA metabolism in SFO treatment, decreased activation was not predicted by IPA. The network of interactions (Fig. 3a-b) shows how three key transcription factors, *SREBF1, STAT5A* and *STAT5B* relate with other molecules, notably *FASN, F2RL1, CNTFR, CALB1, ACSS4, ACADVL* and *TRIB3* to downregulate the synthesis of FA, lipid and FA metabolism. Furthermore, effects of several DE genes (*SREBF1, SCNN1A, INSIG1, DB1 TYK2, CLDN7, SORT1, SCP2, ANGPTL4, SLC6A4* and *KCNMA1*) on the concentration of cholesterol and quantity of steroid with a positive z-score indicate activation, though not predicted by IPA (Fig. 4). Cell death and survival were the top molecular and cellular functions involving 65 differentially expressed genes in cows fed 5 % LSO for 28 days as compared to the same cows on the control diet. Sub categories most affected were cell death (63 genes, *P*-value 8.06E-08), apoptosis (48 genes, *P*-value 1.93E-05), necrosis (44 genes, P-value 3.48E-05) and cell survival (22 genes, P-value 3.91E-03) (Fig. 5, and Additional file 15). Several genes were predicted to decrease cell death and apoptosis based on their expression direction (Additional files 16 and 17).

Top canonical pathways that were significantly enriched by both treatments when day-14 was compared with day+28 included oncostatin M signaling, IL-22 signaling and role of JAK family kinases in IL-6-type cytokine signaling while two pathways each were unique to LSO (TR/RXR Activation, unfolded protein response) and SFO (role of JAK2 in hormone-like cytokine signaling, antioxidant action of vitamin C) treatments (Table 7, Additional files 9 and 10). When comparing day+7 with day+28, only one pathway, LXR/RXR activation, was common to both treatments while four pathways were unique to each treatment (Table 7, Additional files 9 and 10). In line with results of DE genes, several top canonical pathways including UDP-N-acetyl-D-glucosamine Biosynthesis II and UDP-N-acetyl-D-galactosamine biosynthesis II were un-regulated only in LSO treatment when day-14 and day+7 were compared (Table 7, Additional file 9).

**Table 7:** ^a^Top canonical pathways up-regulated by differentially expressed genes in mammary glands of cows whose diets were supplemented with 5 % linseed oil or 5 % safflower oil

Experimental periods compared	Linseed oil treatment			Safflower oil treatment		
	Top Canonical pathways	*P*-value	FDR BH *P*-value	Top Canonical pathways	*P*-value	FDR BH *P*-value
Day-14 vs day+7	Complement System	1.27E-05	4.75E-03	-	-	-
	UDP-N-acetyl-D-glucosamine Biosynthesis II	1.94E-04	3.63E-02	-	-	-
	UDP-N-acetyl-D-galactosamine Biosynthesis II	7.75E-04	8.68E-02	-	-	-
	Unfolded protein response	1.08E-03	8.68E-02	-	-	-
	Antigen Presentation Pathway	1.16E-03	8.68E-02	-	-	-
Day+7 vs day+28	Role of Tissue Factor in Cancer	7.81E-05	2.93E-02	Mitochondrial Dysfunction	1.42E-04	2.54E-02
	FXR/RXR Activation	1.11E-03	2.11E-01	Antioxidant Action of Vitamin C	4.82E-04	3.50E-02
	Arginine Degradation I (Arginase Pathway)	2.37E-03	2.42E-01	Zymosterol Biosynthesis	6.37E-04	3.50E-02
	Growth Hormone Signaling	2.67E-03	2.42E-01	Oxidative Phosphorylation	7.83E-04	3.50E-02
	^b^LXR/RXR Activation	3.18E-03	2.42E-01	LXR/RXR Activation	1.25E-03	4.48E-02
Day-14 vs day+28	Oncostatin M Signaling	1.18E-04	4.19E-02	Oncostatin M Signaling	4.71E-04	1.77E-02
	IL-22 Signaling	7.43E-04	5.21E-02	IL-22 Signaling	1.65E-04	1.29E-02
	Role of JAK family kinases in IL-6-type Cytokine Signaling	8.40E-04	5.21E-02	Role of JAK family kinases in IL-6-type Cytokine Signaling	1.86E-04	1.29E-02
	TR/RXR Activation	6.44E-04	5.21E-02	Role of JAK2 in Hormone-like Cytokine Signaling	5.13E-04	1.77E-02
	Unfolded protein response	7.16E-04	5.21E-02	Antioxidant Action of Vitamin C	9.88E-04	2.73E-02

^a^Top canonical pathways were the first five enriched pathways with unadjusted *P*-values <0.002. ^b^Highlighted pathways are common between linseed oil and safflower oil treatments

Overall, the top networks that were significantly enriched after 28 days of feeding USFAs to cows as compared to the control period (same cows) were lipid metabolism, small molecule biochemistry, cell morphology, cellular function and maintenance, cell-to-cell signaling and interaction, cellular growth and proliferation, posttranslational modifications, metabolic disease, protein synthesis and carbohydrate metabolism (Additional files 9 and 10). The relationships between DE genes in the network of genes implicated in lipid metabolism/small molecule biochemistry/molecular transport are shown in Additional file 18.

## Discussion

In this study, next-generation RNA sequencing technology was used to reflect a biological system’s repertoire of RNA molecules in the bovine mammary gland following dietary supplementation with 5 % LSO or 5 % SFO. Data from the control and treated periods were all drawn from the same animals giving the opportunity to measure the effect of treatments on gene expression without the influence of different genetic components. Furthermore, experimental animals were all in the same stage of lactation (mid-lactation) and sampled within a short period implying that changes in gene expression due to a change from one stage of lactation to another was not expected to influence gene expression results in this study.

Supplementation of cow’s diets with USFAs decreased feed intake significantly but body weights increased (*P* < 0.05) (LSO) or were not affected (SFO) implying that there was enough energy from supplemental oils to support animal’s growth. Our data are supported by previous observations [14, 15]. Although a study found no effect of addition of up to 4 % LSO to the diets of dairy cows on feed intake [13], our data suggest that addition of 5 % LSO or 5 % SFO affected palatability of feed and consequently feed intake, which was more apparent during the first week of supplementation. Similarly, four weeks of dietary supplementation of cow diets with 5 % LSO or 5 % LSO decreased milk FP by 30.38 % (LSO) or 32.21 % (SFO), which supports earlier investigations [6, 24, 53–58]. These reductions are confirmed with the observed significant reductions in the concentrations of majority of SFAs measured. For example, the concentration of C16:0, one of the most abundant SFAs in cow milk decreased by 50 % (LSO) or 53 % (SFO) at the end of supplementation. Our data thus confirm the effectiveness of LSO and SFO to increase the concentrations of healthy FAs in milk while decreasing SFAs.

Previous studies have shown that the number of genes expressed in the bovine mammary gland increases with increasing days in lactation, with the highest at peak lactation followed by gradual decline with increasing days in lactation [33]. The number of genes expressed at mi-lactation and pattern of highly expressed genes in this study reflects previous information [33, 36] and validates RNA-Seq as a valuable tool in the study of mammary gland biology. The genes of the main proteins in milk, *CSN1S1, CSN1S2, CSN2, CSN3, LGB* and *LALBA*, as expected were highly expressed thus concurring with the abundance of these proteins in milk and with a previous study [33]. Furthermore, the expression of these genes were not affected by treatments which supports the observed lack of significant effects of LSO on test-day PP and agrees with previous investigations [34, 59]. *GLYCAM1* detected as highly expressed was also reported amongst highly expressed genes at day 15 of lactation [33]. *GLYCAM1* codes for a milk fat globule membrane protein and is a member of the mucin gene family. Some of the top expressed genes (Table 3) have functions related with milk fat synthesis/uptake (*FABP3, SCD*) and mammary gland health (*SPP1, MFGE8* and *EEF1A1*).

The PUFA content was increased significantly in the milk of treated cows, more so by LS0 (147.76 %) than by SFO (67.84 %). Milk FAs arise from *de novo* synthesis in the mammary gland or are taken up from circulation (~50:50) [5]. In this study, genes with roles in the synthesis of long chain PUFAs, like *FADS1*, *FADS2*, *FADS3*, were not affected by treatments suggesting that treatments increased the availability of PUFAs that were up taken from circulation into milk. Furthermore, LSO (rich in α-linolenic acid with three double bonds) and SFO (rich in linoleic acid with two double bonds) appeared to have affected the biohydrogenation process and associated pathways differently leading to ruminal outflow of a wide range of biohydrogenation metabolites that were eventually absorbed and incorporated into milk. Furthermore, these metabolites, especially those resulting from biohydrogenation of LSO could have affected more pathways and also with greater intensity (Fig. 2, Additional files 9, 10) and could explain the higher number of DE genes by LSO. It is known that the composition of the diet affect ruminal biohydrogenation pathways in a dynamic way leading to the production of a wide range of positional and geometric isomers and modified FAs including CLA:10c12c and a multitude of non-conjugated and partially conjugated C18:2 and C18:3 isomers [12, 13, 60, 61]. It was demonstrated that feeding dairy cows with increasing levels (2, 3 or 4 %) of inclusion of linseed oil resulted in a linear increase in the proportions of several intermediates of ruminal biohydrogenation products of PUFAs, like C18:1 t10, C18:1 t11 (trans vaccenic acid), C18:2c9t11 (CLA), C18:2t11c15 and C18:3c9t11c15 [13]. In this study, the concentrations of C18:1n11t and CLA:10t12c were significantly increased by both treatments and CLA:9c11t by LSO only. It has been demonstrated that a great proportion of milk CLA:9c11t is produced endogenously in the mammary gland using rumen produced C18:1 t11 as a substrate [62]. It is also likely that, other intermediates were also further desaturated to long chain PUFAs in the mammary gland. Our data suggest that increased milk PUFA content during supplemental feeding with LSO and SFO was due to (1) increased availability of biohydrogenation metabolites (mostly PUFAs) that were up taken and incorporated into milk and (2) increased availability of FA metabolites that were further desaturated to longer chain PUFAs in the mammary gland.

On the other hand, total SFA was decreased by 63.52 % (LSO) or 47.09 % (SFO) after four weeks of supplementation. Individual SFAs most affected were short and medium chain FAs, which are those FAs mostly synthesized *de novo*. Several genes known to play roles in *de novo* FA synthesis, including *SREBF1 (*sterol regulatory element binding factor1)*, FASN (fatty acid synthase)*, *ACSS1* (acetyl-CoA short chain synthetase1) and potential new players (Fig. 3) were significantly down regulated by treatments in this study. Activation of FA and cholesterol metabolic pathways requires the coordinated regulation of a network of genes involved in *de novo* FA synthesis such as *FASN* and *ACACA* (acetyl-CoA carboxylase alpha) (down regulated in this study but not significantly), and fatty acid desaturation such as *SCD1* (stearoyl-CoA desaturase 1) (down regulated in this study but not significantly). It has been noted that *SREBF1* (*SREBP1*) affects the mRNA expression levels of *ACACA*, *FASN*, and *SCD* genes in relation to changes in triglyceride content and lipid droplet accumulation in mammary gland epithelial cells *in vitro* thus supporting its regulation of FA synthesis [63]. In dairy cows, support for a central role for SREBP1 in the regulation of mammary lipid synthesis during milk fat depression with CLA isomers has been demonstrated [23]. Decreased expression of *SREBF1* gene in this study correlated with decreased (LSO: *FASN, LPIN1, DBI, ACSS1, ADORA2B, SPTLC3, F2RL1, INSIG1 and STAT5A and B*) or increased (LSO: *SCP2, PDK4, NQO1, CPT1A, CNTFR, CALB1, ACADVL, PIK3CG, PIGZ, TRIB3*) expression of genes involved in the synthesis of FAs/lipids and FA metabolism (Fig. 3). Increased/reduced expression of some of these genes or their protein products have been shown to be involved in the synthesis of lipid/FAs or FA metabolism in rodents, humans and ruminants [64–67] (Additional files 12–14). *INSIG 1* and *2* are known to alter rates of lipogenesis by interacting with *SCAP* (SREBP cleavage activating protein) to regulate the sensitivity of *SREBP1* and *2* processing via *SCAP* [68, 69]. Involvement of *STAT5A* and *5B* in FA metabolism and synthesis of lipid/FA has been demonstrated in mice [64, 70] and polymorphisms in these genes have been associated with milk production traits in cattle [71–73]. In cows with low fat percentages, it was observed that the mRNA expression level of *TRIB3* was higher than in cows with higher fat percentages [34]. Furthermore, *TRIB3* is located in reported QTL regions for milk FP and PP [74, 75]. It should be noted that more of these genes were regulated by LSO as compared to SFO. Our data therefore support roles for *SREBF1*and these genes (Fig. 3) in the down regulation of FA/lipid synthesis. Furthermore, the implication of most of these genes (*LSO: SCP2, PDK4, NQO1, F2RL1, DBI, CPT1A, CNTFR, CALB1, ACADVL, SPTLC3, PIK3CG, PIGZ, ADORA2B* and *TRIB3*. SFO: *SCP2, PDK4, HPGD, ACADVL, IGFBP2* and *TXN*) in the regulation of FA/lipid synthesis and FA metabolism is being reported in bovine for the first time. Our data therefore suggest that decrease in SFAs in this study was mainly due to down-regulation of genes in the FA/lipid synthesis and lipid metabolism pathways. Similarly and supported by previous reports in humans and rodent models [66, 76–79], decreased expression of *SREBF1*and regulation of other genes by USFAs in this study related with increased concentration of cholesterol and quantity of lipid (Fig. 4).

Our data show a greater impact of LSO on mammary gland transcriptome than SFO. However, there were no significant differences in gene expression between treatments mainly due to inter-cow variations. This observation supports earlier findings in cows [30] and goats [80] fed diets supplemented with different USFA rich ingredients (rapeseed oil, soybean oil, linseed oil and sunflower oil). Furthermore, our data show that more genes were significantly differentially regulated by LSO than by SFO supported by miRNA data on the same animals that showed a differential regulation pattern of miRNA expression by LSO and SFO [39].

Genes significantly regulated (≥2 fold change) by both LSO and SFO could be important candidates in shared pathways of mammary lipogenesis/FA uptake like *FBP2, UCP2, TIEG2, ANGPTL4* and *ALDH1L2*. Similarly, genes significantly regulated with ≥2 fold change by either LSO (e.g. *DMGDH, CY2B6, TRIB3, SESN2, BEST3, TMEM132E*, etc.) or SFO (e.g. *ACE2, OLR1, MROH2B*, etc.) could have biological significance in the specific pathways up-regulated by either of the oils. *FBP2*, the most significantly regulated gene in this study, is a regulatory enzyme in gluconeogenesis [81]. In a transgenic mouse model, overexpression of *FBP2* in the liver resulted to lean body weight mostly by reducing adiposity levels by about 50 % and that reductions in food intake and not higher energy expenditure were found to be the contributing factors [82]. FBP2 protein expression is up-regulated in the liver of rats on a high fat diet [83]. Therefore, *FBP2* may be involved in the regulation of lipogenesis when there is increased presence of FAs in circulation or when nutrient availability is reduced due to reduced feed intake. Mitochondrial uncoupling protein 2 (UCP2) and 3, members of the larger family of mitochondrial anion carrier proteins have been implicated in several human health conditions including thermogenesis, obesity, diabetes and heart failure [84]. The mRNA levels of UCPs have been reported to be increased by the presence of long-chain FAs [85, 86]. Our data support these findings [85, 86] and suggest an essential role for *UCP2* in mammary lipogenesis and lipid metabolism in cattle. Kruppel-like factor 11 (*KLF11* or *TIEG2*) is a zinc finger transcription factor that has been implicated in the regulation of complex metabolic diseases [87]. The liver and adipose tissues have been identified as key sources of *ANGPTL4* (angiopoietin-like 4), an adipokine associated with the regulation of lipid metabolism in cattle [88]. Its expression was found to change with altered energy balance in lactating dairy cattle [89]. In this study, the up-regulation of *ANGPTL4* is predicted to increase concentration of cholesterol and quantity of steroid. It was shown in transgenic mice that *ANGPTL3* and *ANGPTL4* regulated circulating triglyceride levels under different nutritional regimes and thus play roles in lipid metabolism through differential inhibition of lipoprotein lipase [77]. *ALDH1L2* (aldehyde dehydrogenase 1 family, member L2) is a member of the aldehyde dehydrogenase superfamily and the formyl transferase superfamily and is known to have enzymatic properties [90]. *UCP2* and *ANGPTL4* also have roles in cell growth and apoptosis and their expression direction were predicted to activate cell death/apoptosis and cell survival in this study (Fig. 5). Taken together, these reports and our data support major roles for these genes (*FBP2, UCP2, TIEG2, ANGPTL4* and *ALDH1L2*) in bovine mammary lipid synthesis/metabolism, cell growth and survival and mammary gland biology. These genes may therefore be potential markers of milk fat synthesis/uptake from circulation.

Mammary specific serum amyloid A3 (*M-SAA3*), *SAA3* and *TRIB3* (tribbles pseudokinase 3) (Additional file 5) were significantly down-regulated by LSO in this study and are amongst genes proposed as candidates for milk protein and fat concentrations in dairy cattle [34]. *SAA3* has been implicated in lipid metabolism, cholesterol transport, and in host defence [91, 92]. The expression of several members of the solute carrier supper gene family was altered by dietary supplements in this study, more so by LSO than SFO (Additional file 7). The solute carrier superfamily encodes membrane bound transporters responsible for transporting substrates including carboxylate, acetyl coenzyme A, vitamins, FAs, lipids and urea across membranes [34]. Since test day FP was decreased by treatments in this study, down-regulation of several of these transporter genes suggest decreased activity of their protein products in transporting FA components and other materials into the mammary gland. Further investigations are needed to clarify this point.

The effect of LSO and SFO on the mammary gland transcriptome resulted in DE genes being enriched in a range of networks, molecular and metabolic pathways related with lipid metabolism, cell death and survival, transport and cellular function and maintenance, which supports findings of a number of studies on bovine milk and mammary tissue transcriptomes under varying lactation conditions [30, 33–35, 37]. Our data further confirm results of miRNA analysis on the same animals that showed target gene enrichment in similar pathways and gene networks [39]. In particular, down-regulation of genes in lipid metabolic pathways and consequently reduced milk FP has been demonstrated in dairy cows in response to USFA supplements including LSO [30, 93] and supported by our data.

Our data show that pathways in cell death and survival were affected by treatments, more so by LSO (Fig. 5 and Additional file 15). Controlled cell death in the mammary gland is vital to maintain proper physiological processes and functioning. Experimental animals were in mid-lactation, a period characterized by a decline in milk production due to a gradual decrease in mammary cell number with advancing days in lactation. This waning in cell number is due to continual cell death by apoptosis which far exceeds the rate of cell renewal [94]. Interestingly, the expression direction (up- or down-regulated) of several genes (Additional files 16 and 17) in this study was predicted by IPA to decrease cell death and apoptosis. When the rate of mammary cell apoptosis/death is decreased after peak lactation, it could ensure greater cell survival and consequently enhanced lactation persistency and productivity. Some of the implicated genes also have roles in lipid synthesis/metabolism like, *SREBF1, STAT5A/5B, SCP2, CNTFR, FASN, ADORA2B, CALB1, TRIB3, F2RL1, PIK3CG* and *PDK4*, thus further emphasizing their significance in mammary gland growth, development and productivity. Our data suggest that supplemental feeding with LSO in addition to enhancing the concentrations of beneficial FAs in milk could also enhance mammary cell survival and lactation persistency.

Three canonical pathways (oncostatin signaling, IL-22 signaling and role of JAK family kinases in IL-6-type cytokine signaling) were significantly enriched by DE genes of both treatments (day-14 vs day+28). Oncostatin M (OSM), a member of the IL-6 family of cytokines mediates its biological effects by binding to receptors on leukemia inhibiting factor receptor or OSM receptor-beta and activation of JAK family members, which in turn leads to activation of STAT proteins. In this study, two STAT genes (*STAT5A* and *5B*) and two further genes (*TIMP3* and *TYK2*) implicated in Oncostatin M signaling, IL-22 signaling/role of JAK family kinases in IL-6-type cytokine signaling (STAT5A and 5B and TYK2) were significantly down-regulated and predicted by IPA to down-regulate significantly Oncostatin M (z-score = −2, *p*-value = 1.18E-04) and JAK/STAT (z-score = −1, p-value = 2.1E-03) signaling pathways by LSO treatment, thus suggesting the involvement of these pathways in mammary lipid metabolism.

The liver X receptors/retinoid X receptors (LXRs/RXRs) pathway was also significantly enriched by DE genes of both treatments (day+7 and day+28 compared). LXRs are members of the nuclear receptor superfamily of transcription factors that regulate the transcription of several genes involved in cholesterol metabolism [95]. LXR function by activating genes that promote the elimination of/limit the accumulation of cellular cholesterol when in excess [95]. In this study, eight and five DE molecules in respectively LSO and SFO treatments were enriched in this pathway and positive Z-scores (Z-score = 0.816 for LSO and 1.00 for SFO) which denote activation, suggest the involvement of this pathway in lipid metabolism/cholesterol metabolism in dairy cattle.

A huge difference between the two types of supplemental oils was seen during the first 7 days of supplementation resulting in DE genes and enriched pathways in LSO but not for SFO when Day-14 was compared with day+7 (Tables 4, 5 and 7). Six DE genes enriched in the complement system showed a balance in gene expression (3 up-regulated [C2, C3 and C1QA] and 3 down-regulated [CFB, CFI and SERPING1]) which translated to a zero Z-score implying that the complement system (part of the innate immune system) was neither activated nor depressed by LSO treatment. Enrichment of the UDP-N-acetyl-D-glucosamine Biosynthesis II and UDP-N-acetyl-D-galactosamine Biosynthesis II biosynthesis pathways could have contributed to the synthesis of metabolites or activated pathways that contributed to the difference in response by cows fed LSO as compared to SFO.

## Conclusion

Milk FP was decreased by ~30 % by USFA enriched supplements accompanied by reductions in the concentrations of several SFAs while several USFAs were increased. LSO modulated the transcriptome of the mammary gland of dairy cows to a greater extent as compared to SFO by affecting more genes and pathways. Several DE genes (*FBP2, UCP2, TIEG2, ANGPTL4, ALDH1L2*) with ≥2 fold regulation by both treatments are potential candidate genes of mammary lipid metabolism. Involvement of some genes (*SCP2, PDK4, NQO1, F2RL1, DBI, CPT1A, CNTFR, CALB1, ACADVL, SPTLC3, PIK3CG, PIGZ, ADORA2B*, *TRIB3, HPGD, IGFBP2* and *TXN*) in FA/lipid metabolism in dairy cows is being reported in this study for the first time. DE genes were enriched in several pathways including lipid metabolism and cell death/survival. Several DE genes were predicted by IPA to significantly decrease the synthesis of FAs and lipid, and FA metabolism by LSO treatment and synthesis of FA and lipid metabolism by SFO. Expression direction of several DE genes in LSO treatment was predicted to decrease death of mammary cells and could be managed to achieve enhanced lactation persistency and productivity.

Finally, this study provides a broader picture of the transcriptomic events that are involved in mammary gland adaptation to diets rich in USFAs with indication that LSO, rich in α-linolenic acid with 3 double bonds, has a greater impact on mammary gland transcriptome by affecting more genes, pathways and processes as compared to SFO, rich in linoleic acid with 2 double bonds. Our study suggests that decrease in milk SFAs was due to down-regulation of genes in the FA/lipid synthesis and lipid metabolism pathways while increase in PUFAs was due to increased availability of ruminal biohydrogenation metabolites that were up taken and incorporated into milk or used as substrate for the synthesis of long chain PUFAs in the mammary gland. Our study further suggests that DE genes were involved in similar (lipid metabolism, molecular transport, small molecule biochemistry) and different (cell death and survival, protein synthesis, cellular growth and proliferation, and amino acid metabolism for LSO; and energy production, cellular movement, cell cycle and carbohydrate metabolism for SFO) functions/pathways by the two types of supplemental ingredients. Moreover, our study provides further knowledge on mammary lipogenesis and data that can be used to develop new nutritional strategies for a better management of milk increased beneficial FAs.

## Additional files

Additional file 1:
**Ingredients and composition of experimental diets.** (DOCX 18 kb) Additional file 2:
**Genes and primer sequences used in qPCR validation of RNA-sequencing data.** (DOCX 19 kb) Additional file 3:
**Reads and mapping statistics according to treatment.** (XLSX 20 kb) Additional file 4:
**Top expressed genes according to treatment.** The combined reads per kilo base per million mapped reads (RPKM) of seven very highly expressed genes constituted 79.45 % of total reads while RPKM of 24 highly expressed genes constituted 4.79 % of total reads. (PDF 122 kb) Additional file 5:
**a-e: Differentially expressed genes between cows on control diet and the same cows supplemented with 5 % linseed oil for 28 days (day-14 vs day+28, day-14 vs day+7 and day+7 vs day+28).** (XLSX 149 kb) Additional file 6:
**a-d: Differentially expressed genes between cows on control diet and the same cows supplemented with 5 % safflower oil for 28 days (day-14 vs day+28 and day+7 vs day+28).** (XLSX 41 kb) Additional file 7:
**Differentially expressed genes in the solute carrier super gene family.** (XLSX 24 kb) Additional file 8:
**Principal component analysis of the gene expression of samples from cows whose diets were supplemented with 5 % linseed oil or 5 % safflower oil (on dry matter bases).** Neither component 1 and 2 (A), or 2 and 3 (B) could separate the samples according to treatments. Samples were widely dispersed while those from the same animal tended to group together, hinting at wide inter-animal variations. (PDF 178 kb) Additional file 9:
**Differentially expressed genes in linseed oil treatment are enriched in several molecular/cellular and physiological system development functions, diseases and disorders, canonical pathways and network functions.** (DOCX 23 kb) Additional file 10:
**Differentially expressed genes in safflower oil treatment are enriched in several molecular/cellular and physiological system development functions, diseases and disorders, canonical pathways and network functions.** (DOCX 20 kb) Additional file 11:
**a-b: Several functions of lipid metabolism were affected by genes differentially expressed when cows were fed diets supplemented with 5%linseed oil (a) or 5 % safflower oil (b) for 4 weeks as compared to same cows on a control diet.** (XLS 50 kb) Additional file 12:
**Differentially expressed genes implicated in the synthesis of lipid between cows on control diets and same cows supplemented with linseed oil for 28 days.** Synthesis of lipid predicted to decrease (Z-score −2.281, p-value 2.94E-04). (DOCX 23 kb) Additional file 13:
**Differentially expressed genes implicated in fatty acid metabolism between cows on control diets and same cows supplemented with linseed oil for 28 days.** Fatty acid metabolism predicted to decrease (Z-score −2.212, p-value 1.05E-04). (DOCX 22 kb) Additional file 14:
**Differentially expressed genes implicated in the synthesis of fatty acids between cows on control diets and cows supplemented with linseed oil for 28 days.** Synthesis of fatty acid predicted to decrease (Z-score −2.710, p-value 2.00E-05). (DOCX 20 kb) Additional file 15:
**Differentially expressed genes with functions in cell death and apoptosis in linseed oil treatment.** (XLS 35 kb) Additional file 16:
**Differentially expressed genes implicated in cell death of cows in LSO treatment as compared to the same cows on the control diet.** Expression direction of several genes predicted to decrease cell death. (DOCX 34 kb) Additional file 17:
**Differentially expressed genes implicated in apoptosis of cows in LSO treatment as compared to the same cows on the control diet.** Expression direction of several genes predicted to decrease apoptosis. (DOCX 35 kb) Additional file 18:
**The relationships between DE genes (day-14 vs day+28) in the network of genes implicated in lipid metabolism/small molecule biochemistry/molecular transport in (A) linseed oil and (B) safflower oil treatments.** (PDF 226 kb)

## References

[1] Bhattacharya A, Banu J, Rahman M, Causey J, Fernandes G (2006). Biological effects of conjugated linoleic acids in health and disease. J Nutr Biochem.

[2] Salter AM. Dietary fatty acids and cardiovascular disease. Animal. 2013;7(Suppl1):163–71. 10.1017/S1751731111002023 23031737

[3] Kris-Etherton PM, Fleming JA (2015). Emerging nutrition science on fatty acids and cardiovascular disease: nutritionists’ perspectives. Advances in Nutrition: An International Review Journal.

[4] Bauman DE, Griinari JM (2001). Regulation and nutritional manipulation of milk fat: low-fat milk syndrome. Livest Prod Sci.

[5] Bauman DE, Griinari JM (2003). Nutritional regulation of milk fat synthesis. Annu Rev Nutr.

[6] Jenkins TC, Harvatine KJ (2014). Lipid feeding and milk fat depression. Vet Clin N Am Food Anim Pract.

[7] Shingfield KJ, Griinari JM (2007). Role of biohydrogenation intermediates in milk fat depression. Eur J Lipid Sci Technol.

[8] Bell JA, Griinari JM, Kennelly JJ (2006). Effect of safflower oil, flaxseed oil, monensin, and vitamin E on concentration of conjugated linoleic acid in bovine milk fat. J Dairy Sci.

[9] Loor JJ, Ferlay A, Ollier A, Ueda K, Doreau M, Chilliard Y (2005). High-concentrate diets and polyunsaturated oils alter trans and conjugated isomers in bovine rumen, blood, and milk. J Dairy Sci.

[10] Boles JA, Kott RW, Hatfield PG, Bergman JW, Flynn CR (2005). Supplemental safflower oil affects the fatty acid profile, including conjugated linoleic acid, of lamb. J Anim Sci.

[11] Kott RW, Hatfield PG, Bergman JW, Flynn CR, Van Wagoner H, Boles JA (2003). Feedlot performance, carcass composition, and muscle and fat CLA concentrations of lambs fed diets supplemented with safflower seeds. Small Rumin Res.

[12] Palmquist DL, Lock AL, Shingfield KJ, Bauman DE (2005). Biosynthesis of conjugated linoleic acid in ruminants and humans. Adv Food Nutr Res.

[13] Benchaar C, Romero-Pérez GA, Chouinard PY, Hassanat F, Eugene M, Petit HV (2012). Supplementation of increasing amounts of linseed oil to dairy cows fed total mixed rations: Effects on digestion, ruminal fermentation characteristics, protozoal populations, and milk fatty acid composition. J Dairy Sci.

[14] Ueda K, Ferlay A, Chabrot J, Loor JJ, Chilliard Y, Doreau M (2003). Effect of linseed oil supplementation on ruminal digestion in dairy cows fed diets with different forage:concentrate ratios. J Dairy Sci.

[15] Martin C, Rouel J, Jouany JP, Doreau M, Chilliard Y (2008). Methane output and diet digestibility in response to feeding dairy cows crude linseed, extruded linseed, or linseed oil. J Anim Sci.

[16] Bauman DE, Perfield JW, Harvatine KJ, Baumgard LH (2008). Regulation of fat synthesis by conjugated linoleic acid: lactation and the ruminant model. J Nutr.

[17] Harvatine KJ, Robblee MM, Boisclair YR, Bauman DE (2008). Trans-10, cis-12 conjugated linoleic acid (CLA) induces a dose-dependent reduction in milk fat synthesis in C57BL6J mice. J Dairy Sci.

[18] Harvatine KJ, Perfield JW, Bauman DE (2009). Expression of enzymes and key regulators of lipid synthesis is upregulated in adipose tissue during CLA-induced milk fat depression in dairy cows. J Nutr.

[19] Baumgard LH, Corl BA, Dwyer DA, Saebo A, Bauman DE (2000). Identification of the conjugated linoleic acid isomer that inhibits milk fat synthesis. American Journal of Physiology: Regulatory, Integrative and Comparative Physiology.

[20] Baumgard LH, Matitashvili E, Corl BA, Dwyer DA, Bauman DE (2002). Trans-10, cis-12 conjugated linoleic acid decreases lipogenic rates and expression of genes involved in milk lipid synthesis in dairy cows. J Dairy Sci.

[21] Baumgard LH, Sangster JK, Bauman DE (2001). Milk fat synthesis in dairy cows is progressively reduced by increasing supplemental amounts of trans-10, cis-12 conjugated linoleic acid (CLA). J Nutr.

[22] Peterson DG, Matitashvili EA, Bauman DE (2004). The inhibitory effect of trans-10, cis-12 CLA on lipid synthesis in bovine mammary epithelial cells involves reduced proteolytic activation of the transcription factor SREBP-1. The Journal of Nutrition.

[23] Harvatine KJ, Bauman DE (2006). SREBP1 and thyroid hormone responsive spot 14 (S14) are involved in the regulation of bovine mammary lipid synthesis during diet-induced milk fat depression and treatment with CLA. J Nutr.

[24] Shingfield KJ, Bernard L, Leroux C, Chilliard Y (2010). Role of trans fatty acids in the nutritional regulation of mammary lipogenesis in ruminants. Animal.

[25] Jacobs AAA, van Baal J, Smits MA, Taweel HZH, Hendriks WH, van Vuuren AM (2011). Effects of feeding rapeseed oil, soybean oil, or linseed oil on stearoyl-CoA desaturase expression in the mammary gland of dairy cows. J Dairy Sci.

[26] Bionaz M, Loor J (2008). Gene networks driving bovine milk fat synthesis during the lactation cycle. BMC Genomics.

[27] Bionaz M, Loor JJ (2008). ACSL1, AGPAT6, FABP3, LPIN1, and SLC27A6 are the most abundant isoforms in bovine mammary tissue and their expression is affected by stage of lactation. The Journal of Nutrition.

[28] Kadegowda AK, Bionaz M, Piperova LS, Erdman RA, Loor JJ (2009). Peroxisome proliferator-activated receptor-gamma activation and long-chain fatty acids alter lipogenic gene networks in bovine mammary epithelial cells to various extents. J Dairy Sci.

[29] Invernizzi G, Thering B, McGuire M, Savoini G, Loor J (2010). Sustained upregulation of stearoyl-CoA desaturase in bovine mammary tissue with contrasting changes in milk fat synthesis and lipogenic gene networks caused by lipid supplements. Funct Integr Genomics.

[30] Mach N, Jacobs AAA, Kruijt L, van Baal J, Smits MA (2011). Alteration of gene expression in mammary gland tissue of dairy cows in response to dietary unsaturated fatty acids. Animal.

[31] Ma L, Corl BA (2012). Transcriptional regulation of lipid synthesis in bovine mammary epithelial cells by sterol regulatory element binding protein-1. J Dairy Sci.

[32] Harvatine KJ, Bauman DE (2006). SREBP1 and thyroid hormone responsive spot 14 (S14) are involved in the regulation of bovine mammary lipid synthesis during diet-induced milk fat depression and treatment with CLA. J Nutr.

[33] Wickramasinghe S, Rincon G, Islas-Trejo A, Medrano J (2012). Transcriptional profiling of bovine milk using RNA sequencing. BMC Genomics.

[34] Cui X, Hou Y, Yang S, Xie Y, Zhang S, Zhang Y (2014). Transcriptional profiling of mammary gland in Holstein cows with extremely different milk protein and fat percentage using RNA sequencing. BMC Genomics.

[35] Bionaz M, Periasamy K, Rodriguez-Zas SL, Everts RE, Lewin HA, Hurley WL (2012). Old and new stories: revelations from functional analysis of the bovine mammary transcriptome during the lactation cycle. PLoS One.

[36] Canovas A, Rincon G, Bevilacqua C, Islas-Trejo A, Brenaut P, Hovey RC (2014). Comparison of five differentRNAsources to examine the lactating bovine mammary gland transcriptome using RNA-Sequencing. Scientific Reports.

[37] Hosseini A, Sharma R, Bionaz M, Loor JJ (2013). Transcriptomics comparisons of mac-t cells versus mammary tissue during late pregnancy and peak lactation. Advances in Dairy Research.

[38] CCAC: Guidelines on the care and use of farm animals in research, teaching and testing. Canadian Council on Animal Care 2009, (http://www.ccac.ca/Documents/Standards/Guidelines/Farm_Animals.pdf)

[39] Li R, Beaudoin F, Ammah A, Bissonnette N, Benchaar C, Zhao X (2015). Deep sequencing shows microRNA involvement in bovine mammary gland adaptation to diets supplemented with linseed oil or safflower oil. BMC Genomics.

[40] Farr VC, Stelwagen K, Cate LR, Molenaar AJ, McFadden TB, Davis SR (1996). An improved method for the routine biopsy of bovine mammary tissue. J Dairy Res.

[41] O’Fallon JV, Busboom JR, Nelson ML, Gaskins CT (2007). A direct method for fatty acid methyl ester synthesis: application to wet meat tissues, oils, and feedstuffs. J Anim Sci.

[42] Bolger AM, Lohse M, Usadel B. Trimmomatic: a flexible trimmer for Illumina sequence data. Bioinformatics 2014, 30(15):2114-2120. 10.1093/bioinformatics/btu170 PMC4103590 24695404

[43] Elsik CG, Tellam RL, Worley KC, Gibbs RA, Muzny DM, Weinstock GM (2009). The genome sequence of taurine cattle: a window to ruminant biology and evolution. Science.

[44] Trapnell C, Roberts A, Goff L, Pertea G, Kim D, Kelley DR (2012). Differential gene and transcript expression analysis of RNA-seq experiments with TopHat and Cufflinks. Nat Protoc.

[45] Anders S, Pyl PT, Huber W (2015). HTSeq—a Python framework to work with high-throughput sequencing data. Bioinformatics.

[46] Robinson MD, McCarthy DJ, Smyth GK (2010). edgeR: a Bioconductor package for differential expression analysis of digital gene expression data. Bioinformatics.

[47] Rajkumar A, Qvist P, Lazarus R, Lescai F, Ju J, Nyegaard M (2015). Experimental validation of methods for differential gene expression analysis and sample pooling in RNA-seq. BMC Genomics.

[48] Benjamini Y, Hochberg Y (1995). Controlling the false discovery rate: a practical and powerful approach to multiple testing. J R Stat Soc Ser B Methodol.

[49] Anders S, Huber W (2010). Differential expression analysis for sequence count data. Genome Biol.

[50] Livak KJ, Schmittgen TD (2001). Analysis of relative gene expression data using real-time quantitative PCR and the 2 − ΔΔCT method. Methods.

[51] Andersen CL, Jensen JL, Ørntoft TF (2004). Normalization of real-time quantitative reverse transcription-pcr data: a model-based variance estimation approach to identify genes suited for normalization, applied to bladder and colon cancer data sets. Cancer Res.

[52] Chen Y, McCarthy D, Robinson M, Smyth GK. edgeR: differential expression analysis of digital gene expression data User’s Guide. http://bioconductor.org/packages/release/bioc/html/edge.html 2014:1–78. 10.1093/bioinformatics/btp616 PMC2796818 19910308

[53] Ma L, Cook KL, Bauman DE, Harvatine KJ (2015). Short communication: Milk fat depression induced by conjugated linoleic acid and a high-oil and low-fiber diet occurs equally across the day in Holstein cows. J Dairy Sci.

[54] Pappritz J, Meyer U, Kramer R, Weber E-M, Jahreis G, Rehage J (2011). Effects of long-term supplementation of dairy cow diets with rumen-protected conjugated linoleic acids (CLA) on performance, metabolic parameters and fatty acid profile in milk fat. Arch Anim Nutr.

[55] McGuire MA, Griinari JM, Dwyer DA, Bauman DE (1995). Role of insulin in the regulation of mammary synthesis of fat and protein. J Dairy Sci.

[56] Ramirez Ramirez HA, Castillo Lopez E, Harvatine KJ, Kononoff PJ (2015). Fat and starch as additive risk factors for milk fat depression in dairy diets containing corn dried distillers grains with solubles. J Dairy Sci.

[57] Rico DE, Holloway AW, Harvatine KJ (2014). Effect of monensin on recovery from diet-induced milk fat depression. J Dairy Sci.

[58] Rico DE, Ying Y, Clarke AR, Harvatine KJ (2014). The effect of rumen digesta inoculation on the time course of recovery from classical diet-induced milk fat depression in dairy cows. J Dairy Sci.

[59] Finucane K, McFadden T, Bond J, Kennelly J, Zhao F-Q (2008). Onset of lactation in the bovine mammary gland: gene expression profiling indicates a strong inhibition of gene expression in cell proliferation. Funct Integr Genomics.

[60] Jenkins TC, Wallace RJ, Moate PJ, Mosley EE (2008). Board-invited review: Recent advances in biohydrogenation of unsaturated fatty acids within the rumen microbial ecosystem. J Anim Sci.

[61] Lee Y-J, Jenkins TC (2011). Biohydrogenation of linolenic acid to stearic acid by the rumen microbial population yields multiple intermediate conjugated diene isomers. The Journal of Nutrition.

[62] Griinari JM, Corl BA, Lacy SH, Chouinard PY, Nurmela KV, Bauman DE (2000). Conjugated linoleic acid is synthesized endogenously in lactating dairy cows by Δ9-desaturase. J Nutr.

[63] Li N, Zhao F, Wei C, Liang M, Zhang N, Wang C (2014). Function of SREBP1 in the milk fat synthesis of dairy cow mammary epithelial cells. Int J Mol Sci.

[64] Barnstein BO, Li G, Wang Z, Kennedy S, Chalfant C, Nakajima H (2006). Stat5 expression is required for IgE-mediated mast cell function. J Immunol.

[65] Engelking LJ, Liang G, Hammer RE, Takaishi K, Kuriyama H, Evers BM (2005). Schoenheimer effect explained – feedback regulation of cholesterol synthesis in mice mediated by Insig proteins. J Clin Investig.

[66] Amigo L, Zanlungo S, Miquel JF, Glick JM, Hyogo H, Cohen DE (2003). Hepatic overexpression of sterol carrier protein-2 inhibits VLDL production and reciprocally enhances biliary lipid secretion. J Lipid Res.

[67] Grassian AR, Metallo CM, Coloff JL, Stephanopoulos G, Brugge JS (2011). Erk regulation of pyruvate dehydrogenase flux through PDK4 modulates cell proliferation. Genes Dev.

[68] Espenshade PJ, Hughes AL (2007). Regulation of sterol synthesis in eukaryotes. Annu Rev Genet.

[69] Weber LW, Boll M, Stampfl A (2004). Maintaining cholesterol homeostasis: Sterol regulatory element-binding proteins. World J Gastroenterol.

[70] Schirra HJ, Anderson CG, Wilson WJ, Kerr L, Craik DJ, Waters MJ (2008). Altered metabolism of growth hormone receptor mutant mice: a combined nmr metabonomics and microarray study. PLoS One.

[71] Selvaggi M, Dario C, Normanno G, Celano GV, Dario M (2009). Genetic polymorphism of STAT5A protein: relationships with production traits and milk composition in Italian Brown cattle. J Dairy Res.

[72] Khatib H, Monson RL, Schutzkus V, Kohl DM, Rosa GJM, Rutledge JJ (2008). Mutations in the STAT5A Gene are associated with embryonic survival and milk composition in cattle. J Dairy Sci.

[73] He Y, Chu Q, Ma P, Wang Y, Zhang Q, Sun D (2011). Association of bovine CD4 and STAT5b single nucleotide polymorphisms with somatic cell scores and milk production traits in Chinese Holsteins. J Dairy Res.

[74] Kolbehdari D, Wang Z, Grant JR, Murdoch B, Prasad A, Xiu Z (2009). A whole genome scan to map QTL for milk production traits and somatic cell score in Canadian Holstein bulls. J Anim Breed Gen.

[75] Wu X, Fang M, Liu L, Wang S, Liu J, Ding X, et al. Genome wide association studies for body conformation traits in the Chinese Holstein cattle population. BMC Genomics. 2013;14:897–7. 10.1186/1471-2164-14-897 PMC3879203 24341352

[76] Bi L, Chiang JYL, Ding W-X, Dunn W, Roberts B, Li T (2013). Saturated fatty acids activate ERK signaling to downregulate hepatic sortilin 1 in obese and diabetic mice. J Lipid Res.

[77] Köster A, Chao YB, Mosior M, Ford A, Gonzalez-DeWhitt PA, Hale JE (2005). Transgenic Angiopoietin-Like (Angptl)4 overexpression and targeted disruption of angptl4 and angptl3: regulation of triglyceride metabolism. Endocrinology.

[78] Engelking LJ, Kuriyama H, Hammer RE, Horton JD, Brown MS, Goldstein JL (2004). Overexpression of Insig-1 in the livers of transgenic mice inhibits SREBP processing and reduces insulin-stimulated lipogenesis. J Clin Investig.

[79] Charles R-P, Guitard M, Leyvraz C, Breiden B, Haftek M, Haftek-Terreau Z (2008). Postnatal requirement of the epithelial sodium channel for maintenance of epidermal barrier function. J Biol Chem.

[80] Ollier S, Leroux C, de la Foye A, Bernard L, Rouel J, Chilliard Y (2009). Whole intact rapeseeds or sunflower oil in high-forage or high-concentrate diets affects milk yield, milk composition, and mammary gene expression profile in goats. J Dairy Sci.

[81] Wu C, Khan SA, Peng L-J, Lange AJ (2006). Roles for fructose-2,6-bisphosphate in the control of fuel metabolism: beyond its allosteric effects on glycolytic and gluconeogenic enzymes. Adv Enzym Regul.

[82] Visinoni S, Khalid NFI, Joannides CN, Shulkes A, Yim M, Whitehead J (2012). The role of liver fructose-1,6-Bisphosphatase in regulating appetite and adiposity. Diabetes.

[83] Song S, Andrikopoulos S, Filippis C, Thorburn AW, Khan D, Proietto J (2001). Mechanism of fat-induced hepatic gluconeogenesis: effect of metformin. Am J Physiol Endocrinol Metab.

[84] Donadelli M, Dando I, Fiorini C, Palmieri M (2014). UCP2, a mitochondrial protein regulated at multiple levels. Cell Mol Life Sci.

[85] Hsu H-C, Chen C-Y, Chen M-F (2014). N-3 polyunsaturated fatty acids decrease levels of doxorubicin-induced reactive oxygen species in cardiomyocytes -- involvement of uncoupling protein UCP2. J Biomed Sci.

[86] Yonezawa T, Sanosaka M, Haga S, Kobayashi Y, Katoh K, Obara Y (2008). Regulation of uncoupling protein 2 expression by long-chain fatty acids and hormones in bovine mammary epithelial cells. Biochem Biophys Res Commun.

[87] Lomberk G, Grzenda A, Mathison A, Escande C, Zhang J-S, Calvo E (2013). Krüppel-like factor 11 regulates the expression of metabolic genes via an evolutionarily conserved protein interaction domain functionally disrupted in maturity onset diabetes of the young. The Journal of Biological Chemistry.

[88] Mamedova LK, Robbins K, Johnson BJ, Bradford BJ (2010). Tissue expression of angiopoietin-like protein 4 in cattle. J Anim Sci.

[89] Koltes DA, Spurlock DM (2012). Adipose tissue angiopoietin-like protein 4 messenger RNA changes with altered energy balance in lactating Holstein cows. Domest Anim Endocrinol.

[90] Strickland KC, Krupenko NI, Dubard ME, Hu CJ, Tsybovsky Y, Krupenko SA (2011). Enzymatic properties of Aldh1l2, A Mitochondrial 10-Formyltetrahydrofolate Dehydrogenase. Chem Biol Interact.

[91] Molenaar AJ, Harris DP, Rajan GH, Pearson ML, Callaghan MR, Sommer L (2009). The acute-phase protein serum amyloid A3 is expressed in the bovine mammary gland and plays a role in host defence. Biomarkers.

[92] van der Westhuyzen DR, Cai L, de Beer MC, de Beer FC (2005). Serum amyloid a promotes cholesterol efflux mediated by scavenger receptor B-I. J Biol Chem.

[93] Mach N, Zom RLG, Widjaja HCA, van Wikselaar PG, Weurding RE, Goselink RMA (2013). Dietary effects of linseed on fatty acid composition of milk and on liver, adipose and mammary gland metabolism of periparturient dairy cows. J Anim Physiol Anim Nutr.

[94] Capuco AV, Wood DL, Baldwin R, McLeod K, Paape MJ (2001). Mammary cell number, proliferation, and apoptosis during a bovine lactation: relation to milk production and effect of bST1. J Dairy Sci.

[95] Repa JJ, Turley SD, Lobaccaro J-MA, Medina J, Li L, Lustig K (2000). Regulation of absorption and ABC1-mediated efflux of cholesterol by RXR heterodimers. Science.

