# Supplementary material for: Transcriptome adaptation of the bovine mammary gland to diets rich in unsaturated fatty acids shows greater impact of linseed oil over safflower oil on gene expression and metabolic pathways
Source: BMC Genomics. 2016 Feb 9;17:104. doi: 10.1186/s12864-016-2423-x (PMC4748538; doi:10.1186/s12864-016-2423-x)
Supplement: Additional file 1: — Ingredients and composition of experimental diets. (DOCX 18 kb) [file 12864_2016_2423_MOESM1_ESM.docx]

**Additional file 1**

**Ingredients and composition of the experimental diets**

| **Ingredients** | | **Treatments** |  | |
| --- | --- | --- | --- | --- |
| (% of dry matter) | | **Control diet** | **Diet safflower oil** | **Diet linseed oil** |
| Chopped hay | | 3.3 | 3.3 | 3.3 |
| Corn silage | | 28.6 | 28.6 | 28.6 |
| Hay silage | | 28.5 | 28.5 | 28.5 |
| Corn grain | | 23.4 | 23.4 | 23.4 |
| Soybean meal | | 10.6 | 10.6 | 10.6 |
| ^1^Protein supplements | | 3.7 | 3.7 | 3.7 |
| Calcium carbonate | | 0.4 | 0.4 | 0.4 |
| Iodide mineral supplement | | 1.5 | 1.5 | 1.5 |
| Safflower oil | | - | 5 | - |
| Linseed oil | | - | - | 5 |
| ^1^Protein supplement | | 3.7 | 3.7 | 3.7 |
| Fatty acid composition of oil (g/100g) | |  | Safflower oil | Linseed oil |
|  | C4:0 | - | 0.000 | 0.000 |
|  | C8:0 | - | 0.000 | 0.000 |
|  | C14:0 | - | 0.021 | 0.142 |
|  | C14:1 | - | 0.065 | 0.000 |
|  | C14:1t | - | 0.083 | 0.057 |
|  | C16:0 | - | 5.570 | 8.939 |
|  | C18:0 | - | 2.590 | 2.851 |
|  | C18:1n9c | - | 15.400 | 6.244 |
|  | C18:2n6cc (linoleic acid) | - | 64.800 | 14.016 |
|  | C18:3n3 (α-linolenic acid) | - | 0.001 | 70.818 |
|  | C20:0 | - | 0.002 | 0.300 |
|  | C22:5n3 | - | 0.005 | 0.056 |
|  | C22:5n6 | - | 0.029 | 0.426 |
|  | C20:0 | - | 0.002 | 0.300 |

^1^Protein supplement: 30 % corn gluten feed, 30% corn distiller’s grain, 20%canola meal and

20% heat treated soybean meal.
