# Supplementary material for: Transcriptome adaptation of the bovine mammary gland to diets rich in unsaturated fatty acids shows greater impact of linseed oil over safflower oil on gene expression and metabolic pathways
Source: BMC Genomics. 2016 Feb 9;17:104. doi: 10.1186/s12864-016-2423-x (PMC4748538; doi:10.1186/s12864-016-2423-x)
Supplement: Additional file 2: — Genes and primer sequences used in qPCR validation of RNA-sequencing data. (DOCX 19 kb) [file 12864_2016_2423_MOESM2_ESM.docx]

**Additional file 2**

**Genes and primer sequences used in qPCR validation of RNA-sequencing data**

| Target gene | Accession number | Primer | Concentration (nM) | Sequence | Amplicon length |
| --- | --- | --- | --- | --- | --- |
| SREBF1 | NM_001113302.1 | Forward | 300 | 5’-GAC TAC ATC CGC TTC CTT CAG-3’ | 81 |
|  |  | Reverse | 300 | 5’-CCA GGT CCT TCA GCG ATT-3’ |  |
| MROH2B | XM_005221571.2 | Forward | 300 | 5’-CCT GCA GAC CTG ATG GAT TT-3’ | 88 |
|  |  | Reverse | 300 | 5’-TGG GCT CTT CAG CAT TGA TAG-3’ |  |
| RASD1 | NM_001206261.1 | Forward | 300 | 5’-TTC CAC CGC AAG TTC TAC TG-3’ | 102 |
|  |  | Reverse | 300 | 5’-GAT GAA CAC GTC TCC GGT AAG-3’ |  |
| FASN | NM_001012669.1 | Forward | 900 | 5’-AGC ACA CGC CTG TAG TAT TC-3’ | 77 |
|  |  | Reverse | 900 | 5’-TTC CAG AAG CCG CAC TT-3’ |  |
| FBP2 | NM_001046164.2 | Forward | 300 | 5’-CCT GGT GTC GGA AGA GAA TAA A-3’ | 98 |
|  |  | Reverse | 300 | 5’-AGG CAG TCA ATG TTG GAA GA-3’ |  |
| ACE2 | NM_001024502.4 | Forward | 300 | 5’-GGG AGT GGT AGT GAT TGG TAT TG-3’ | 89 |
|  |  | Reverse | 300 | 5’-TTC AAG TCC ACA GAG CCA TAA G-3’ |  |
| UCP2 | NM_001033611.2 | Forward | 300 | 5’-GAC GTG GTC AAG ACG AGA TAC-3’ | 101 |
|  |  | Reverse | 900 | 5’-AGG GCA TGA ACC CTT TGT AG-3’ |  |
| TIEG2 (KLF11) | NM_001190301.1 | Forward | 300 | 5’-ACC AAA GGA CTT CCA TTC TCT ATC-3’ | 70 |
|  |  | Reverse | 300 | 5’-GGA ACA ACT GTC CCA GTC AA-3’ |  |
| TC2N | NM_001193204.1 | Forward | 300 | 5’-TCA TCT CAG CGG TTC ATT CAG-3’ | 100 |
|  |  | Reverse | 300 | 5’-GTG TCC AGG CTT CTG TTA CTC-3’ |  |
| CSN2 | XM_010806178.1 | Forward | 900 | 5’-CAG AGG ATG AAC TCC AGG ATA AA-3’ | 97 |
|  |  | Reverse | 900 | 5’-GGT TTG AGT AAG AGG AGG GAT G-3’ |  |
| GAPDH | NM_001034034.2 | Forward | 300 | 5’-TGG AAA GGC CAT CAC CAT CT-3’ | 62 |
|  |  | Reverse | 300 | 5’-CCC ACT TGA TGT TGG CAG-3’ |  |
| UXT | NM_001037471.2 | Forward | 300 | 5’-TGT GGC CCT TGG ATA TGG TT-3’ | 81 |
|  |  | Reverse | 900 | 5’-GGT TGT CGC TGA GCT CTG TG-3’ |  |
| RPS9 | NM_001101152.2 | Forward | 300 | 5’-TTT CCA GAG CGT TGG CTT AG-3’ | 114 |
|  |  | Reverse | 300 | 5’-GGA CTT CTC GAA GGG TCT CC-3’ |  |
| RPS15 | NM_001024541.2 | Forward | 300 | 5’-GAT CAT TCT ACC CGA GAT GGT G-3’ | 127 |
|  |  | Reverse | 300 | 5’-GGG CTT GTA AGT GAT GGA GAA-3’ |  |
