# Supplementary material for: Transcriptome adaptation of the bovine mammary gland to diets rich in unsaturated fatty acids shows greater impact of linseed oil over safflower oil on gene expression and metabolic pathways
Source: BMC Genomics. 2016 Feb 9;17:104. doi: 10.1186/s12864-016-2423-x (PMC4748538; doi:10.1186/s12864-016-2423-x)
Supplement: Additional file 4: — Top expressed genes according to treatment. The combined reads per kilo base per million mapped reads (RPKM) of seven very highly expressed genes constituted 79.45 % of total reads while RPKM of 24 highly expressed genes constituted 4.79 % of total reads. (PDF 122 kb) [file 12864_2016_2423_MOESM4_ESM.pdf]

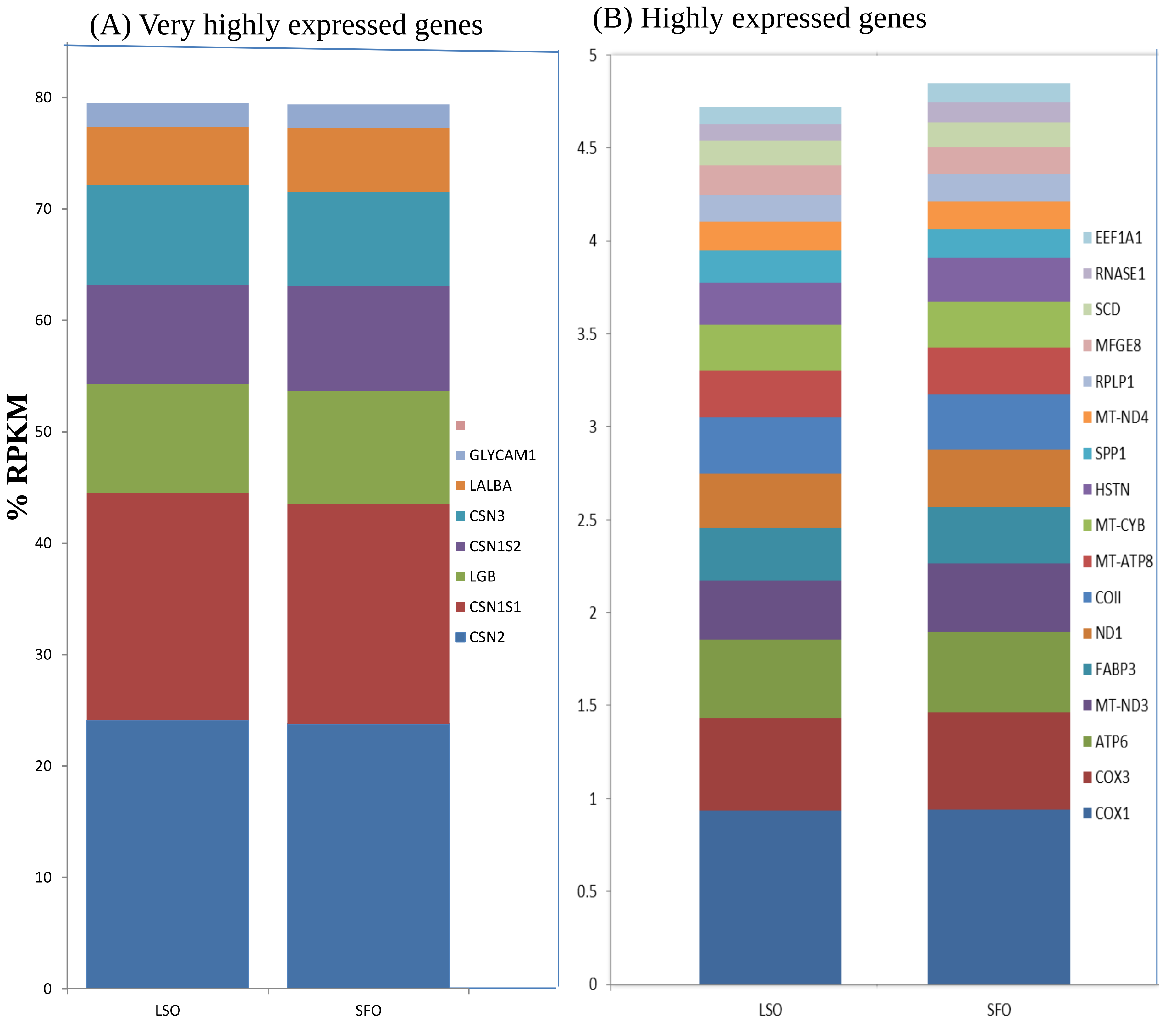

Additional file 4: Top expressed genes according to treatment. The combined reads per kilo base per million mapped reads (RPKM) of seven very highly expressed genes constituted 79.45% of total reads while RPKM of 24 highly expressed genes constituted 4.79% of total reads.
