# Supplementary material for: Transcriptome adaptation of the bovine mammary gland to diets rich in unsaturated fatty acids shows greater impact of linseed oil over safflower oil on gene expression and metabolic pathways
Source: BMC Genomics. 2016 Feb 9;17:104. doi: 10.1186/s12864-016-2423-x (PMC4748538; doi:10.1186/s12864-016-2423-x)
Supplement: Additional file 9: — Differentially expressed genes in linseed oil treatment are enriched in several molecular/cellular and physiological system development functions, diseases and disorders, canonical pathways and network functions. (DOCX 23 kb) [file 12864_2016_2423_MOESM9_ESM.docx]

**Additional file 9**

**Differentially expressed genes in linseed oil treatment are enriched in several molecular/cellular and physiological system development functions, diseases and disorders, canonical pathways and network functions**

|  | **Top Canonical pathways** | **P-value** | **FDR BH P-value** |
| --- | --- | --- | --- |
| **LSDay-14 vs day+7** | Complement System | 1.27E-05 | 4.75E-03 |
|  | UDP-N-acetyl-D-glucosamine Biosynthesis II | 1.94E-04 | 3.63E-02 |
|  | UDP-N-acetyl-D-galactosamine Biosynthesis II | 7.75E-04 | 8.68E-02 |
|  | Unfolded protein response | 1.08E-03 | 8.68E-02 |
|  | Antigen Presentation Pathway | 1.16E-03 | 8.68E-02 |
|  |  |  |  |
| **LSDay+7 vs day+28** | Role of Tissue Factor in Cancer | 7.81E-05 | 2.93E-02 |
|  | FXR/RXR Activation | 1.11E-03 | 2.11E-01 |
|  | Arginine Degradation I (Arginase Pathway) | 2.37E-03 | 2.42E-01 |
|  | Growth Hormone Signaling | 2.67E-03 | 2.42E-01 |
|  | LXR/RXR Activation | 3.18E-03 | 2.42E-01 |
|  |  |  |  |
| **LSDay-14 vs day+28** | Oncostatin M Signaling | 1.18E-04 | 4.19E-02 |
|  | TR/RXR Activation | 6.44E-04 | 5.21E-02 |
|  | Unfolded protein response | 7.16E-04 | 5.21E-02 |
|  | IL-22 Signaling | 7.43E-04 | 5.21E-02 |
|  | Role of JAK family kinases in IL-6-type Cytokine Signaling | 8.40E-04 | 5.21E-02 |

**Top Diseases and disorders (Linseed treatment)**

|  | **Name** | **P-value** | **FDR BH P-value** | **#molecules** |
| --- | --- | --- | --- | --- |
| **LSD-14 vs 7** | Inflammatory Response | 3.81E-03 - 5.15E-10 | 2.94E-06 – 4.36E-02 | 119 |
|  | Cancer | 3.81E-03 - 1.59E-09 | 4.54E-06 – 4.36E-02 | 397 |
|  | Organismal Injury and Abnormalities | 3.81E-03 - 1.59E-09 | 4.54E-06 – 4.36E-02 | 398 |
|  | Cardiovascular Disease | 3.18E-03 - 4.88E-09 | 5.80E-06 – 3.86E-02 | 61 |
|  | Immunological Disease | 3.81E-03 - 9.63E-08 | 4.54E-06 – 4.36E-02 | 133 |
|  |  |  |  |  |
| **LSD7 vs 28** | Cancer | 3.32E-03 - 1.16E-09 | 8.55E-06-4.88E-02 | 369 |
|  | Organismal Injury and Abnormalities | 3.32E-03 - 1.16E-09 | 8.55E-06-4.88E-02 | 371 |
|  | Cardiovascular Disease | 2.88E-03 - 4.67E-08 | 8.6E-05-4.4E-02 | 70 |
|  | Reproductive System Disease | 3.32E-03 - 3.06E-07 | 2.05E-04-4.88E-02 | 103 |
|  | Dermatological Diseases and Conditions | 1.57E-03 - 6.33E-07 | 2.68E-04-3.27E-02 | 49 |
|  |  |  |  |  |
| **LSD-14 vs 28** | Cancer | 1.38E-02 - 4.26E-09 | 1.23E-05-6.98E-02 | 148 |
|  | Organismal Injury and Abnormalities | 1.38E-02 - 4.26E-09 | 1.23E-05-6.98E-02 | 149 |
|  | Reproductive System Disease | 1.27E-02 - 1.60E-06 | 5E-04-6.98E-02 | 60 |
|  | Metabolic Disease | 1.17E-02 - 2.72E-06 | 6.05E-04-6.98E-02 | 49 |
|  | Respiratory Disease | 1.27E-02 - 3.45E-06 | 6.66E-04-6.98E-02 | 10 |

**Molecular and Cellular Functions (Linseed treatment)**

|  | **Name** | **P-value** | **FDR BH P-value** | **#molecules** |
| --- | --- | --- | --- | --- |
| LSD-14 vs 7 | Cellular Function and Maintenance | 3.81E-03 - 4.09E-09 | 5.80E-06 – 4.36E-02 | 132 |
|  | Cellular Movement | 3.62E-03 - 8.87E-09 | 7.23E-06 – 4.23#-02 | 93 |
|  | Cell-To-Cell Signaling and Interaction | 3.81E-03 - 3.37E-08 | 2.40E-05 – 4.36E-02 | 69 |
|  | Cellular Growth and Proliferation | 3.83E-03 - 1.23E-07 | 4.66E-05 – 4.37E-02 | 149 |
|  | Cell Morphology | 3.42E-03 - 2.14E-07 | 6.80E-05 – 4.07E-02 | 73 |
|  |  |  |  |  |
| LSD7 vs 28 | Cell Death and Survival | 3.10E-03 - 4.04E-08 | 8.6E-05-4.61E-02 | 144 |
|  | Molecular Transport | 3.13E-03 - 1.61E-07 | 1.48E-04-4.66E-02 | 109 |
|  | Lipid Metabolism | 3.13E-03 - 1.96E-07 | 1.6E-04-4.66E-02 | 81 |
|  | Small Molecule Biochemistry | 3.13E-03 - 1.96E-07 | 1.6E-04-4.66E-02 | 105 |
|  | Cellular Function and Maintenance | 2.37E-03 - 3.73E-07 | 2.05E-04-3.87E-02 | 83 |
|  |  |  |  |  |
| LSD-14 vs 28 | Cell Death and Survival | 1.35E-02 - 8.60E-08 | 8.31E-05-6.98E-02 | 65 |
|  | Lipid Metabolism | 1.38E-02 - 1.53E-06 | 5E-04-6.98E-02 | 40 |
|  | Molecular Transport | 1.38E-02 - 1.53E-06 | 5E-04-6.98E-02 | 43 |
|  | Small Molecule Biochemistry | 1.38E-02 - 1.53E-06 | 5E-04-6.98E-02 | 60 |
|  | Protein Synthesis | 1.17E-02 - 8.26E-06 | 1.26E-03-6.98E-02 | 22 |

**Physiological system development and function (Linseed treatment)**

|  | **Name** | **P-value** | **FDR BH P-value** | **#molecules** |
| --- | --- | --- | --- | --- |
| **LSD-14 vs 7** | Hematological System Development and Function | 3.81E-03 - 5.08E-09 | 5.80E-06 – 4.36E-02 | 97 |
|  | Tissue Morphology | 3.44E-03 - 5.08E-09 | 5.80E-06 – 4.07E-02 | 75 |
|  | Immune Cell Trafficking | 3.81E-03 - 8.87E-09 | 7.23E-06 – 4.36E-02 | 68 |
|  | Humoral Immune Response | 2.82E-03 - 1.01E-06 | 1.98^E^-04 – 3.55^E^-02 | 40 |
|  | Cardiovascular System Development and Function | 2.32E-03 - 1.61E-06 | 2.63E-04 – 3.37E-02 | 62 |
|  |  |  |  |  |
| **LSD7 vs 28** | Cardiovascular System Development and Function | 3.17E-03 - 3.91E-07 | 2.05E-04-4.69E-02 | 81 |
|  | Tissue Development | 2.81E-03 - 3.91E-07 | 2.05E-04-4.35E-02 | 108 |
|  | Organismal Development | 3.10E-03 - 5.26E-07 | 2.58E-04-4.61E-02 | 131 |
|  | Tissue Morphology | 3.31E-03 - 7.65E-07 | 2.96E-04-4.88E-02 | 101 |
|  | Organismal Survival | 1.45E-05 - 2.40E-06 | 6.3E-04-2.05E-03 | 108 |
|  |  |  |  |  |
| **LSD-14 vs 28** | Organismal Survival | 8.56E-03 - 1.87E-06 | 5E-04-5.65E-02 | 53 |
|  | Cardiovascular System Development and Function | 1.31E-02 - 1.90E-06 | 5E-04-6.98E-02 | 24 |
|  | Hematological System Development and Function | 1.24E-02 - 1.90E-06 | 5E-04-6.98E-02 | 32 |
|  | Connective Tissue Development and Function | 1.27E-02 - 3.05E-06 | 6.32E-04-6.98E-02 | 30 |
|  | Tissue Morphology | 1.17E-02 - 3.05E-06 | 6.32E-04-6.98E-02 | 44 |

**Top Networks (Linseed treatment)**

|  | **ID** | **Associated network functions** | **^1^Score** |
| --- | --- | --- | --- |
| **LSD-14 vs 7** | 1 | Gene Expression, Protein Synthesis, Developmental Disorder | 42 |
|  | 2 | Auditory Disease, Hereditary Disorder, Neurological Disease | 39 |
|  | 3 | Carbohydrate Metabolism, Cancer, Organismal Injury and Abnormalities | 39 |
|  | 4 | Gastrointestinal Disease, Cell Morphology, Cellular Assembly and Organization | 33 |
|  | 5 | Cell Morphology, Cellular Assembly and Organization, Cancer | 31 |
|  |  |  |  |
| **LSD7 vs 28** | 1 | Developmental Disorder, Hereditary Disorder, Metabolic Disease | 130 |
|  | 2 | Cancer, Organismal Injury and Abnormalities, Connective Tissue Disorders | 101 |
|  | 3 | Endocrine System Development and Function, Small Molecule Biochemistry, Developmental Disorder | 88 |
|  | 4 | Cardiovascular Disease, Gene Expression, Cancer | 82 |
|  | 5 | Inflammatory Response, Hematological System Development and Function, Tissue Morphology | 71 |
|  |  |  |  |
| **LSD-14 vs 28** | 1 | Hereditary Disorder, Metabolic Disease, Neurological Disease | 80 |
|  | 2 | Lipid Metabolism, Small Molecule Biochemistry, Molecular Transport | 43 |
|  | 3 | Cancer, Cell Morphology, Cellular Function and Maintenance | 33 |
|  | 4 | Developmental Disorder, Hematological Disease, Hereditary Disorder | 33 |
|  | 5 | Neurological Disease, Developmental Disorder, Hereditary Disorder | 26 |

^1^The score, a numerical valued used to rank networks, takes into account the number of Network Eligible molecules in the network and its size, as well as the total number of Network Eligible molecules in the dataset and the total number of molecules in the Ingenuity Knowledge Base that could potentially be included in networks. Networks are thus ordered according to their score, with the highest scoring network displayed at the top.
