# Supplementary material for: Transcriptome adaptation of the bovine mammary gland to diets rich in unsaturated fatty acids shows greater impact of linseed oil over safflower oil on gene expression and metabolic pathways
Source: BMC Genomics. 2016 Feb 9;17:104. doi: 10.1186/s12864-016-2423-x (PMC4748538; doi:10.1186/s12864-016-2423-x)
Supplement: Additional file 10: — Differentially expressed genes in safflower oil treatment are enriched in several molecular/cellular and physiological system development functions, diseases and disorders, canonical pathways and network functions. (DOCX 20 kb) [file 12864_2016_2423_MOESM10_ESM.docx]

**Additional file 10**

**Differentially expressed genes in safflower oil treatment are enriched in several molecular/cellular and physiological system development functions, diseases and disorders, canonical pathways and network functions**

|  | **Top Canonical pathways** | **P-value** | **FDR BH P-value** |
| --- | --- | --- | --- |
| **SFDay+7 vs day+28** | Mitochondrial Dysfunction | 1.42E-04 | 2.54E-02 |
|  | Antioxidant Action of Vitamin C | 4.82E-04 | 3.50E-02 |
|  | Zymosterol Biosynthesis | 6.37E-04 | 3.50E-02 |
|  | Oxidative Phosphorylation | 7.83E-04 | 3.50E-02 |
|  | LXR/RXR Activation | 1.25E-03 | 4.48E-02 |
| **SFDay-14 vs day+28** | IL-22 Signaling | 1.65E-04 | 1.29E-02 |
|  | Role of JAK family kinases in IL-6-type Cytokine Signaling | 1.86E-04 | 1.29E-02 |
|  | Oncostatin M Signaling | 4.71E-04 | 1.77E-02 |
|  | Role of JAK2 in Hormone-like Cytokine Signaling | 5.13E-04 | 1.77E-02 |
|  | Antioxidant Action of Vitamin C | 9.88E-04 | 2.73E-02 |

**Top Diseases and disorders (Safflower treatment)**

|  | **Name** | **P-value** | **FDR BH P-value** | **#molecules** |
| --- | --- | --- | --- | --- |
| **SFD7 vs 28** | Cancer | 6.60E-03 - 2.49E-07 | 3.77E-04-3.49E-02 | 120 |
|  | Organismal Injury and Abnormalities | 6.60E-03 - 2.49E-07 | 3.77E-04-3.49E-02 | 123 |
|  | Developmental Disorder | 6.60E-03 - 5.77E-07 | 3.77E-04-3.49E-02 | 27 |
|  | Hereditary Disorder | 6.60E-03 - 5.77E-07 | 3.77E-04-3.49E-02 | 29 |
|  | Metabolic Disease | 6.60E-03 - 5.77E-07 | 3.77E-04-3.49E-02 | 46 |
| **SFD-14 vs 28** | Developmental Disorder | 2.22E-02 - 5.23E-06 | 2.41E-03-7.87E-02 | 16 |
|  | Hereditary Disorder | 2.22E-02 - 5.23E-06 | Hereditary Disorder | 22 |
|  | Metabolic Disease | 2.66E-02 - 5.23E-06 | Metabolic Disease | 33 |
|  | Nutritional Disease | 7.58E-03 - 3.77E-05 | Nutritional Disease | 15 |
|  | Cancer | 2.66E-02 - 8.34E-05 | 7.25E-03-8.68E-02 | 80 |

**Molecular and Cellular Functions (Safflower treatment)**

|  | **Name** | **P-value** | **FDR BH P-value** | **#molecules** |
| --- | --- | --- | --- | --- |
| **SFD7 vs 28** | Cellular Movement | 5.68E-03 - 2.21E-08 | 7.22E-05-3.49E-02 | 41 |
|  | Cell Death and Survival | 6.60E-03 - 4.54E-06 | 1.3E-03-3.49E-02 | 50 |
|  | Cellular Function and Maintenance | 6.60E-03 - 8.09E-06 | 1.55E-03-3.49E-02 | 35 |
|  | Lipid Metabolism | 6.60E-03 - 1.25E-05 | 1.7E-03-3.49E-02 | 39 |
|  | Small Molecule Biochemistry | 6.60E-03 - 1.25E-05 | 1.7E-03-3.49E-02 | 54 |
| **SFD-14 vs 28** | Lipid Metabolism | 2.22E-02 - 3.85E-08 | 6.37E-05-7.87E-02 | 29 |
|  | Small Molecule Biochemistry | 2.66E-02 - 3.85E-08 | 6.37E-05-8.68E-02 | 33 |
|  | Energy Production | 1.34E-02 - 1.47E-07 | 1.21E-04-6.11E-02 | 11 |
|  | Molecular Transport | 2.22E-02 - 8.97E-06 | 2.41E-03-7.87E-02 | 24 |
|  | Cellular Movement | 2.36E-02 - 1.01E-05 | 2.41E-03-8.19E-02 | 27 |

**Physiological system development and function (Safflower treatment)**

|  | **Name** | **P-value** | **FDR BH P-value** | **#molecules** |
| --- | --- | --- | --- | --- |
| **SFD7 vs 28** | Hematopoiesis | 6.60E-03 - 8.09E-06 | 1.55E-03-3.49E-02 | 8 |
|  | Cardiovascular System Development and Function | 6.60E-03 - 2.06E-05 | 2.07E-03-3.49E-02 | 22 |
|  | Hematological System Development and Function | 6.60E-03 - 2.06E-05 | 2.07E-03-3.49E-02 | 34 |
|  | Connective Tissue Development and Function | 6.60E-03 - 2.85E-05 | 2.16E-03-3.49E-02 | 25 |
|  | Tissue Morphology | 6.60E-03 - 2.85E-05 | 2.16E-03-3.49E-02 | 36 |
|  |  |  |  |  |
| **SFD-14 vs 28** | Hematological System Development and Function | 2.61E-02 - 5.65E-05 | 6.13E-03-8.68E-02 | 17 |
|  | Tissue Morphology | 2.61E-02 - 5.65E-05 | 6.13E-03-8.68E-02 | 25 |
|  | Organ Morphology | 2.66E-02 - 5.94E-05 | 6.13E-03-8.68E-02 | 14 |
|  | Skeletal and Muscular System Development and Function | 2.66E-02 - 5.94E-05 | 6.13E-03-8.68E-02 | 11 |
|  | Reproductive System Development and Function | 2.22E-02 - 8.01E-05 | 7.25E-03-7.87E-02 | 11 |

Top Networks (Safflower treatment)

|  | **ID** | **Associated network functions** | **^1^Score** |
| --- | --- | --- | --- |
| **SFD7 vs 28** | 1 | Developmental Disorder, Hereditary Disorder, Metabolic Disease | 40 |
|  | 2 | Cardiovascular Disease, Cellular Function and Maintenance, Hematopoiesis | 33 |
|  | 3 | Amino Acid Metabolism, Small Molecule Biochemistry, Drug Metabolism | 30 |
|  | 4 | Cellular Development, Cellular Growth and Proliferation, Cell-To-Cell Signaling and Interaction | 28 |
|  | 5 | Carbohydrate Metabolism, Molecular Transport, Small Molecule Biochemistry | 26 |
|  |  |  |  |
| **SFD-14 vs 28** | 1 | Lipid Metabolism, Molecular Transport, Small Molecule Biochemistry | 36 |
|  | 2 | Post-Translational Modification, Lipid Metabolism, Small Molecule Biochemistry | 33 |
|  | 3 | Developmental Disorder, Hereditary Disorder, Metabolic Disease | 31 |
|  | 4 | Organ Morphology, Skeletal and Muscular System Development and Function, Protein Synthesis | 28 |
|  | 5 | Cell-To-Cell Signaling and Interaction, Cellular Growth and Proliferation, Carbohydrate Metabolism | 28 |

^1^The score, a numerical valued used to rank networks, takes into account the number of Network Eligible molecules in the network and its size, as well as the total number of Network Eligible molecules in the dataset and the total number of molecules in the Ingenuity Knowledge Base that could potentially be included in networks. Networks are thus ordered according to their score, with the highest scoring network displayed at the top.
