# Supplementary material for: Transcriptome adaptation of the bovine mammary gland to diets rich in unsaturated fatty acids shows greater impact of linseed oil over safflower oil on gene expression and metabolic pathways
Source: BMC Genomics. 2016 Feb 9;17:104. doi: 10.1186/s12864-016-2423-x (PMC4748538; doi:10.1186/s12864-016-2423-x)
Supplement: Additional file 12: — Differentially expressed genes implicated in the synthesis of lipid between cows on control diets and same cows supplemented with linseed oil for 28 days. Synthesis of lipid predicted to decrease (Z-score −2.281, p-value 2.94E-04). (DOCX 23 kb) [file 12864_2016_2423_MOESM12_ESM.docx]

**Additional file 12**

**Differentially expressed genes implicated in the synthesis of lipid between cows on control diets and same cows supplemented with linseed oil for 28 days. Synthesis of lipid predicted to decrease (Z-score -2.281, p-value 2.94E-04)**

| **^1^Genes in dataset** | **Prediction (based on expression direction of genes in dataset)** | **Fold change** | **Literature findings (references)** |
| --- | --- | --- | --- |
| SCP2 | Decreased | 1.290 | Decreases (Amigo et al. 2002, Yamamoto et al. 1991) |
| CNTFR | Decreased | 1.793 | Decreases (Dinarello et al. 1996) |
| STAT5A | Decreased | -1.317 | Increases (1) Barnstein et al. 2006) |
| FASN | Decreased | -1.520 | Increases (Schmitt et al. 2011; Elis et al. 2013; Jensen-Urstad and Semenkovich, 2012) |
| ACADVL | Decreased | 1.401 | Decreases (Chen et al.2008) |
| DBI | Decreased | -1.354 | Increases (Yang et al. 2001, Zhang et al. 2012) |
| ADORA2B | Decreased | -1.804 | Increases (Chen et al. 2008) |
| CALB1 | Decreased | 2.990 | Decreases (Rabinovitch et al. 2001) |
| STAT5B | Decreased | -1.328 | Increases (Barnstein et al. 2006) |
| TRIB3 | Decreased | 2.456 | Decreases (Qi et al. 2006) |
| SREBF1 | Decreased | -1.645 | Increases (Weber et al. 2004; Huang et al. 2012) |
| F2RL1 | Decreased | -1.320 | Increases (Nichols et al. 2012) |
| LPIN1 | Decreased | -2.805 | Increases (Schmitt et al. 2011; Meana et al. 2014) |
| INSIG1 | Increased | -1.638 | Decreases (10) Engelking et al. 2005, 2006 |
| PIK3CG | Increased | 1.788 | Increases (Rommel et al. 2007) |
| SPTLC3 | Affected | -1.418 | Affects (Han et al. 2009) |
| ACSS1 | Affected | -1.319 | Affects (Schwer et al. 2006; Fujino et al. 2001) |
| PDK4 | Affected | 2.142 | Affects (Hwang et al. 2009; Grassian et al. 2011) |
| PIGZ | Affected | 1.559 | Affects (Taron et al. 2004) |

^1^13 of 19 genes have expression direction consistent with decreases in synthesis of lipid

**References**

Amigo L, Zanlungo S, Miquel JF, Glick JM, Hyogo H, Cohen DE, Rigotti A, Nervi F. Hepatic overexpression of sterol carrier protein-2 inhibits VLDL production and reciprocally enhances biliary lipid secretion. J Lipid Res. 2003 Feb;44(2):399-407. Epub 2002 Nov 4.

Barnstein BO, Li G, Wang Z, Kennedy S, Chalfant C, Nakajima H, Bunting KD, Ryan JJ. Stat5 expression is required for IgE-mediated mast cell function. J Immunol. 2006 Sep 1;177(5):3421-6.

Chen YC, Huang SH, Wang SM. Adenosine-stimulated adrenal steroidogenesis involves the adenosine A2A and A2B receptors and the Janus kinase 2-mitogen-activated protein kinase kinase-extracellular signal-regulated kinase signaling pathway. Int J Biochem Cell Biol. 2008;40(12):2815-25. Epub 2008 Jun 5

Dinarello CA. Biologic basis for interleukin-1 in disease. Blood. 1996 Mar 15;87(6):2095-147.

Elis S, Coyral-Castel S, Freret S, Cognié J, Desmarchais A, Fatet A, Rame C, Briant E, Maillard V, Dupont J. Expression of adipokine and lipid metabolism genes in adipose tissue of dairy cows differing in a female fertility quantitative trait locus. J Dairy Sci. 2013 Dec;96(12):7591-602. Epub 2013 Oct 11.

Engelking LJ, Evers BM, Richardson JA, Goldstein JL, Brown MS, Liang G. Severe facial clefting in Insig-deficient mouse embryos caused by sterol accumulation and reversed by lovastatin. J Clin Invest. 2006 Sep;116(9):2356-65.

Engelking LJ, Liang G, Hammer RE, Takaishi K, Kuriyama H, Evers BM, Li WP, Horton JD, Goldstein JL, Brown MS. Schoenheimer effect explained--feedback regulation of cholesterol synthesis in mice mediated by Insig proteins. J Clin Invest. 2005 Sep;115(9):2489-98. Epub 2005 Aug 11.

Fujino T, Kondo J, Ishikawa M, Morikawa K, Yamamoto TT. Acetyl-CoA synthetase 2, a mitochondrial matrix enzyme involved in the oxidation of acetate. J Biol Chem. 2001 Apr 6;276(14):11420-6. Epub 2001 Jan 9.

Grassian AR, Metallo CM, Coloff JL, Stephanopoulos G, Brugge JS. Erk regulation of pyruvate dehydrogenase flux through PDK4 modulates cell proliferation. Genes Dev. 2011 Aug 15;25(16):1716-33.

Han G, Gupta SD, Gable K, Niranjanakumari S, Moitra P, Eichler F, Brown RH, Harmon JM, Dunn TM. Identification of small subunits of mammalian serine palmitoyltransferase that confer distinct acyl-CoA substrate specificities. Proc Natl Acad Sci U S A. 2009 May 19;106(20):8186-91. Epub 2009 May 5.

Huang WC, Li X, Liu J, Lin J, Chung LW. Activation of androgen receptor, lipogenesis, and oxidative stress converged by SREBP-1 is responsible for regulating growth and progression of prostate cancer cells. Mol Cancer Res. 2012 Jan;10(1):133-42. Epub 2011 Nov 7.

Hwang B, Jeoung NH, Harris RA. Pyruvate dehydrogenase kinase isoenzyme 4 (PDHK4) deficiency attenuates the long-term negative effects of a high-saturated fat diet. Biochem J. 2009 Oct 15;423(2):243-52. Epub 2009 Sep 25.

Meana C, Peña L, Lordén G, Esquinas E, Guijas C, Valdearcos M, Balsinde J, Balboa MA. Lipin-1 Integrates Lipid Synthesis with Proinflammatory Responses during TLR Activation in Macrophages. J Immunol. 2014 Nov 1;193(9):4614-22. Epub 2014 Sep 24.

Moon YS, Latasa MJ, Griffin MJ, Sul HS. Suppression of fatty acid synthase promoter by polyunsaturated fatty acids. J Lipid Res. 2002 May;43(5):691-8.

Nichols HL, Saffeddine M, Theriot BS, Hegde A, Polley D, El-Mays T, Vliagoftis H, Hollenberg MD, Wilson EH, Walker JK, DeFea KA. β-Arrestin-2 mediates the proinflammatory effects of proteinase-activated receptor-2 in the airway. Proc Natl Acad Sci U S A. 2012 Oct 9;109(41):16660-5. Epub 2012 Sep 25.

Qi L, Heredia JE, Altarejos JY, Screaton R, Goebel N, Niessen S, Macleod IX, Liew CW, Kulkarni RN, Bain J, Newgard C, Nelson M, Evans RM, Yates J, Montminy M. TRB3 links the E3 ubiquitin ligase COP1 to lipid metabolism. Science. 2006 Jun 23;312(5781):1763-6.

Rabinovitch A, Suarez-Pinzon WL, Sooy K, Strynadka K, Christakos S. Expression of calbindin-D(28k) in a pancreatic islet beta-cell line protects against cytokine-induced apoptosis and necrosis. Endocrinology. 2001 Aug;142(8):3649-55.

Rommel C, Camps M, Ji H. PI3K delta and PI3K gamma: partners in crime in inflammation in rheumatoid arthritis and beyond? Nat Rev Immunol. 2007 Mar;7(3):191-201. Epub 2007 Feb 9.

Schmitt E, Ballou MA, Correa MN, DePeters EJ, Drackley JK, Loor JJ. Dietary lipid during the transition period to manipulate subcutaneous adipose tissue peroxisome proliferator-activated receptor-γ co-regulator and target gene expression. J Dairy Sci. 2011 Dec;94(12):5913-25.

Schwer B, Bunkenborg J, Verdin RO, Andersen JS, Verdin E. Reversible lysine acetylation controls the activity of the mitochondrial enzyme acetyl-CoA synthetase 2. Proc Natl Acad Sci U S A. 2006 Jul 5;103(27):10224-9. Epub 2006 Jun 20.

Taron BW, Colussi PA, Wiedman JM, Orlean P, Taron CH. Human Smp3p adds a fourth mannose to yeast and human glycosylphosphatidylinositol precursors in vivo. J Biol Chem. 2004 Aug 20;279(34):36083-92. Epub 2004 Jun 18.

Weber LW, Boll M, Stampfl A. Maintaining cholesterol homeostasis: sterol regulatory element-binding proteins. World J Gastroenterol. 2004 Nov 1;10(21):3081-7.

Yamamoto R, Kallen CB, Babalola GO, Rennert H, Billheimer JT, Strauss JF. Cloning and expression of a cDNA encoding human sterol carrier protein 2. Proc Natl Acad Sci U S A. 1991 Jan 15;88(2):463-7.

Yang Y, Pritchard PH, Bhuiyan J, Seccombe DW, Moghadasian MH. Overexpression of acyl-coA binding protein and its effects on the flux of free fatty acids in McA-RH 7777 cells. Lipids. 2001 Jun;36(6):595-600.

Zhang J, Diaz A, Mao L, Ahlquist P, Wang X. Host acyl coenzyme A binding protein regulates replication complex assembly and activity of a positive-strand RNA virus. J Virol. 2012 May;86(9):5110-21. Epub 2012 Feb 15.
