# Supplementary material for: Transcriptome adaptation of the bovine mammary gland to diets rich in unsaturated fatty acids shows greater impact of linseed oil over safflower oil on gene expression and metabolic pathways
Source: BMC Genomics. 2016 Feb 9;17:104. doi: 10.1186/s12864-016-2423-x (PMC4748538; doi:10.1186/s12864-016-2423-x)
Supplement: Additional file 13: — Differentially expressed genes implicated in fatty acid metabolism between cows on control diets and same cows supplemented with linseed oil for 28 days. Fatty acid metabolism predicted to decrease (Z-score −2.212, p-value 1.05E-04). (DOCX 22 kb) [file 12864_2016_2423_MOESM13_ESM.docx]

**Additional file13**

**Differentially expressed genes implicated in fatty acid metabolism between cows on control diets and same cows supplemented with linseed oil for 28 days. Fatty acid metabolism predicted to decrease (Z-score -2.212, P-value 1.05E-04)**

| ^1^Genes in dataset | Prediction (based on expression direction of genes in dataset) | Fold change | Literature findings (references) |
| --- | --- | --- | --- |
| CNTFR | Decreased | 1.793 | Decreases (Dinarello et al. 1996) |
| STAT5A | Decreased | -1.317 | Increases (Schirra et al. 2008, Barnstein et al. 2006) |
| FASN | Decreased | -1.520 | Increases (Elis et al. 2013, Jensen-Urstad and Semenkovich 2012) |
| ACADVL | Decreased | 1.401 | Decreases (Goetzman et al. 2005) |
| CALB1 | Decreased | 2.990 | Decreases (Rabinovitch et al. 2001) |
| STAT5B | Decreased | -1.328 | Increases (Schirra et al. 2008, Barnstein et al. 2006) |
| TRIB3 | Decreased | 2.456 | Decreases (Qi et al. 2006) |
| SREBF1 | Decreased | -1.645 | Increases (Weber et al. 2004, Huang et al. 2012) |
| F2RL1 | Decreased | -1.320 | Increases (Nichols et al. 2012) |
| CPT1A | Increased | 2.512 | Increases (Akkaoui et al. 2009 Rao et al. 2011) |
| INSIG1 | Increased | -1.638 | Decreases (Engelking et al. 2005) |
| LPIN1 | Affected | -2.805 | Affects (Finck et al. 2006) |
| NQO1 | Affected | 1.358 | Affects (Iskander and Jaiswal 2005) |
| DBI | Affected | -1.354 | Affects (Kolmer et al. 1993) |
| ACSS1 | Affected | -1.319 | Affects (Fujino et al. 2001, Schwer et al. 2006) |
| PDK4 | Affected | 2.142 | Affects (Grassian et al. 2011, Hwang et al. 2009) |
| SCP2 | Affected | 1.290 | Affects (Amigo et al. 2003, Schroeder et al. 2003) |

^1^9 of 17 genes have expression direction consistent with decreases in fatty acid metabolism

**References**

Akkaoui M, Cohen I, Esnous C, Lenoir V, Sournac M, Girard J, Prip-Buus C. Modulation of the hepatic malonyl-CoA-carnitine palmitoyltransferase 1A partnership creates a metabolic switch allowing oxidation of de novo fatty acids. Biochem J. 2009 Jun 15;420(3):429-38. Epub 2009 May 27.

Amigo L, Zanlungo S, Miquel JF, Glick JM, Hyogo H, Cohen DE, Rigotti A, Nervi F. Hepatic overexpression of sterol carrier protein-2 inhibits VLDL production and reciprocally enhances biliary lipid secretion. J Lipid Res. 2003 Feb;44(2):399-407. Epub 2002 Nov 4.

Barnstein BO, Li G, Wang Z, Kennedy S, Chalfant C, Nakajima H, Bunting KD, Ryan JJ. Stat5 expression is required for IgE-mediated mast cell function. J Immunol. 2006 Sep 1;177(5):3421-6.

Dinarello CA. Biologic basis for interleukin-1 in disease. Blood. 1996 Mar 15;87(6):2095-147.

Elis S, Coyral-Castel S, Freret S, Cognié J, Desmarchais A, Fatet A, Rame C, Briant E, Maillard V, Dupont J. Expression of adipokine and lipid metabolism genes in adipose tissue of dairy cows differing in a female fertility quantitative trait locus. J Dairy Sci. 2013 Dec;96(12):7591-602. Epub 2013 Oct 11.

Engelking LJ, Liang G, Hammer RE, Takaishi K, Kuriyama H, Evers BM, Li WP, Horton JD, Goldstein JL, Brown MS. Schoenheimer effect explained--feedback regulation of cholesterol synthesis in mice mediated by Insig proteins. J Clin Invest. 2005 Sep;115(9):2489-98. Epub 2005 Aug 11.

Finck BN, Gropler MC, Chen Z, Leone TC, Croce MA, Harris TE, Lawrence JC, Kelly DP. Lipin 1 is an inducible amplifier of the hepatic PGC-1alpha/PPARalpha regulatory pathway. Cell Metab. 2006 Sep;4(3):199-210.

Fujino T, Kondo J, Ishikawa M, Morikawa K, Yamamoto TT. Acetyl-CoA synthetase 2, a mitochondrial matrix enzyme involved in the oxidation of acetate. J Biol Chem. 2001 Apr 6;276(14):11420-6. Epub 2001 Jan 9.

Goetzman ES, Tian L, Wood PA. Differential induction of genes in liver and brown adipose tissue regulated by peroxisome proliferator-activated receptor-alpha during fasting and cold exposure in acyl-CoA dehydrogenase-deficient mice. Mol Genet Metab. 2005 Jan;84(1):39-47. Epub 2004 Nov 11.

Grassian AR, Metallo CM, Coloff JL, Stephanopoulos G, Brugge JS. Erk regulation of pyruvate dehydrogenase flux through PDK4 modulates cell proliferation. Genes Dev. 2011 Aug 15;25(16):1716-33.

Huang WC, Li X, Liu J, Lin J, Chung LW. Activation of androgen receptor, lipogenesis, and oxidative stress converged by SREBP-1 is responsible for regulating growth and progression of prostate cancer cells. Mol Cancer Res. 2012 Jan;10(1):133-42. Epub 2011 Nov 7.

| Hwang B, Jeoung NH, Harris RA. Pyruvate dehydrogenase kinase isoenzyme 4 (PDHK4) deficiency attenuates the long-term negative effects of a high-saturated fat diet. Biochem J. 2009 Oct 15;423(2):243-52. Epub 2009 Sep 25. |
| --- |

Iskander K, Jaiswal AK. Quinone oxidoreductases in protection against myelogenous hyperplasia and benzene toxicity. Chem Biol Interact. 2005 May 30;153-154:147-57. Epub 2005 Apr 7.

Jensen-Urstad AP, Semenkovich CF. Fatty acid synthase and liver triglyceride metabolism: housekeeper or messenger? Biochim Biophys Acta. 2012 May;1821(5):747-53. Epub 2011 Oct 8.

Kolmer M, Alho H, Costa E, Pani L. Cloning and tissue-specific functional characterization of the promoter of the rat diazepam binding inhibitor, a peptide with multiple biological actions. Proc Natl Acad Sci U S A. 1993 Sep 15;90(18):8439-43.

Nichols HL, Saffeddine M, Theriot BS, Hegde A, Polley D, El-Mays T, Vliagoftis H, Hollenberg MD, Wilson EH, Walker JK, DeFea KA. β-Arrestin-2 mediates the proinflammatory effects of proteinase-activated receptor-2 in the airway. Proc Natl Acad Sci U S A. 2012 Oct 9;109(41):16660-5. Epub 2012 Sep 25.

Qi L, Heredia JE, Altarejos JY, Screaton R, Goebel N, Niessen S, Macleod IX, Liew CW, Kulkarni RN, Bain J, Newgard C, Nelson M, Evans RM, Yates J, Montminy M. TRB3 links the E3 ubiquitin ligase COP1 to lipid metabolism. Science. 2006 Jun 23;312(5781):1763-6.

Rabinovitch A, Suarez-Pinzon WL, Sooy K, Strynadka K, Christakos S. Expression of calbindin-D(28k) in a pancreatic islet beta-cell line protects against cytokine-induced apoptosis and necrosis. Endocrinology. 2001 Aug;142(8):3649-55.

Rao JN, Warren GZ, Estolt-Povedano S, Zammit VA, Ulmer TS. An environment-dependent structural switch underlies the regulation of carnitine palmitoyltransferase 1A. J Biol Chem. 2011 Dec 9;286(49):42545-54. Epub 2011 Oct 11.

Schirra HJ, Anderson CG, Wilson WJ, Kerr L, Craik DJ, Waters MJ, Lichanska AM. Altered metabolism of growth hormone receptor mutant mice: a combined NMR metabonomics and microarray study. PLoS One. 2008;3(7):e2764. Epub 2008 Jul 23.

Schroeder F, Zhou M, Swaggerty CL, Atshaves BP, Petrescu AD, Storey SM, Martin GG, Huang H, Helmkamp GM, Ball JM. Sterol carrier protein-2 functions in phosphatidylinositol transfer and signaling. Biochemistry. 2003 Mar 25;42(11):3189-202.

Schwer B, Bunkenborg J, Verdin RO, Andersen JS, Verdin E. Reversible lysine acetylation controls the activity of the mitochondrial enzyme acetyl-CoA synthetase 2. Proc Natl Acad Sci U S A. 2006 Jul 5;103(27):10224-9. Epub 2006 Jun 20.

Weber LW, Boll M, Stampfl A. Maintaining cholesterol homeostasis: sterol regulatory element-binding proteins. World J Gastroenterol. 2004 Nov 1;10(21):3081-7.
