# Supplementary material for: Transcriptome adaptation of the bovine mammary gland to diets rich in unsaturated fatty acids shows greater impact of linseed oil over safflower oil on gene expression and metabolic pathways
Source: BMC Genomics. 2016 Feb 9;17:104. doi: 10.1186/s12864-016-2423-x (PMC4748538; doi:10.1186/s12864-016-2423-x)
Supplement: Additional file 16: — Differentially expressed genes implicated in cell death of cows in LSO treatment as compared to the same cows on the control diet. Expression direction of several genes predicted to decrease cell death. (DOCX 34 kb) [file 12864_2016_2423_MOESM16_ESM.docx]

**Additional file 16**

**Differentially expressed genes implicated in cell death between cows on control diets and same cows supplemented with linseed oil for 28 days. Cell death with a positive Z-score (0.555, P-value 8.6E-08) indicates a small degree of activation**

| Genes in  dataset | ^1^Prediction (based on expression direction) | Fold change | Literature findings (references) |  |
| --- | --- | --- | --- | --- |
| RNASE1 | Increased | 3.182 | Increases (Piccoli et al. 1999) |  |
| CALB1 | Decreased | 2.990 | Decreases (Cluskey et al. 2001, Jeon et al. 2004) |  |
| KLF11 (TIEG2) | Increased | 2.592 | Increases (Fernandez-Zapico et al. 2011) |  |
| TRIB3 | Increased | 2.456 | Increases (Shimizu et al. 2012, Wu et al. 2003) |  |
| CYP2B6 | Increased | 2.371 | Increases (Jounaidi et al. 200) |  |
| UCP2 | Decreased | 2.256 | Decreases (Deng et al. 2012, Diano et al. 2003) |  |
| ANGPTL4 | Decreased | 2.166 | Decreases (Hou et al. 2014, Kim et al. 2000) |  |
| HBEGF | Decreased | 2.006 | Decreases (Fischer et al. 2004, Zhang et al. 2014) |  |
| AGR2 | Increased | 1.850 | Increases (Ryu et al. 2013) |  |
| ATF5 | Decreased | 1.820 | Decreases (Persengiev et al. 2002) |  |
| CNTFR | Decreased | 1.793 | Decreases (Mittoux et al. 2002) |  |
| PIK3CG | Decreased | 1.788 | Decreases (Rommel et al. 2007, Sasaki et al. 2000) |  |
| PCK2 | Decreased | 1.714 | Decreases (Méndez-Lucas et al. 2014) |  |
| RAPGEF4 | Decreased | 1.657 | Decreases (Ahmed et al. 2011) |  |
| UBC | Affected | 1.563 | Affects (Ryu et al. 2007) |  |
| DDIT3 | Increased | 1.553 | Increases (Lovat et al. 2002, Loinard et al. 2012) |  |
| G0S2 | Increased | 1.535 | Increases (Welch et al. 2009) |  |
| ASNS | Decreased | 1.495 | Decreases (Cui et al. 2007) |  |
| ARF4 | Decreased | 1.478 | Decreases (Woo et al. 2008) |  |
| ATP2B2 | Affected | 1.435 | Affects (Jiang et al. 2010) |  |
| TBX3 | Decreased | 1.396 | Decreases (Carlson et al. 2002, Ito et al. 2005) |  |
| GSR | Increased | 1.384 | Increases (Ye et al. 1999) |  |
| SORT1 | Increased | 1.368 | Increases (Nykjaer et al. 2004, Campagnolo et al. 2014) |  |
| NQO1 | Increased | 1.358 | Increases (Kung et al. 2014, Zhang et al. 2014) |  |
| KCNMA1 | Decreased | 1.336 | Decreases (Rüttiger et al. 2004) |  |
| GNG2 | Increased | 1.309 | Increases (Giambarella et al. 1997) |  |
| DOCK8 | Increased | 1.294 | Increases (Ham et al. 2013) |  |
| SCP2 | Increased | 1.290 | Increases (Kriska et al. 2006, Atshaves et al. 2002) |  |
| NOC2L | Decreased | 1.280 | Decreases (Wu et al. 2011) |  |
| MME | Decreased | 1.265 | Decreases (Sumitomo et al. 2001) |  |
| GINS1 | Affected | 1.250 | Affects (Ueno et al. 2005) |  |
| LIG3 | Increased | -1.252 | Decreases (Bordone et al. 2002) |  |
| SLC2A1 | Increased | -1.252 | Decreases (Zhao et al. 2007) |  |
| CLDN7 | Increased | -1.279 | Decreases (Nübel et al. 2007) |  |
| STEAP3 | Decreased | -1.279 | Increases (Steiner et al. 2000, Passer et al. 2003) |  |
| BACH1 | Decreased | -1.288 | Increases (Balan and Pal, 2014) |  |
| STAT5A | Increased | -1.317 | Decreases (Casetti et al. 2013, Ahonen et al. 2003) |  |
| F2RL1 | Decreased | -1.320 | Increases (Peng et al. 2013) |  |
| TIMP3 | Decreased | -1.328 | Increases (Bond et al. 2000, Drynda et al. 2005) |  |
| STAT5B | Increased | -1.328 | Decreases (Behbod et al. 2003, Casetti et al. 2013) |  |
| ITSN1 | Increased | -1.329 | Decreases (Das et al. 2007, Predescu et al. 2007) |  |
| F2R | Decreased | -1.375 | Increases (Niessen et al. 2008) |  |
| TYK2 | Decreased | -1.377 | Increases (Ghoreschi et al. 2009) |  |
| PKP2 | Affected | -1.391 | Affects (Kim et al. 2013) |  |
| RAC2 | Affected | -1.420 | Affects (Sengupta et al. 2010) |  |
| CSRNP1 | Affected | -1.433 | Affects (Cheng et al. 2013) |  |
| ADD3 | Affected | -1.454 | Affects (Sahr et al. 2009) |  |
| TRPM2 | Increased | -1.458 | Decreases (Gao et al. 2010) |  |
| FASN | Increased | -1.520 | Decreases (Shiragami et al. 2013, Bandyopadhyay et al. 2005) |  |
| SH3BP5 | Increased | -1.542 | Decreases (Chambers et al. 2013) |  |
| ITGAL | Affected | -1.590 | Affects (Moretta et al. 2008) |  |
| SREBF1 | Decreased | -1.645 | Increases (Wang et al. 2003) |  |
| RRAD | Decreased | -1.673 | Increases (Sun et al. 2011) |  |
| RASD1 | Decreased | -1.719 | Increases (Vaidyanathan et al. 2004) |  |
| UBD | Decreased | -1.757 | Increases (Raasi et al. 2001) |  |
| ADCYAP1R1 | Increased | -1.759 | Decreases (Pilzer and Gozes 2006) |  |
| ADORA2B | Decreased | -1.804 | Increases (Long et al. 2013, Csóka et al. 2010) |  |
| ISG20 | Affected | -1.841 | Affects (Zhong et al. 2010) |  |
| CENPJ | Increased | -1.855 | Decreases (Hung et al. 2004) |  |
| EMP1 | Decreased | -1.877 | Increases (Wilson et al. 2002) |  |
| CAPN6 | Increased | -1.928 | Decreases (Liu et al. 2011) |  |
| ARG2 | Increased | -1.942 | Decreases (Lewis et al. 2011) |  |
| NAD+ (C3) | Increased | -1.942 | Decreases (Pliyev et al. 2014, Pillai et al. 2005) |  |

^1^Expression direction of 27 genes in this study decreases cell death and expressed direction of 27 genes increases cell death

**References**

Ahmed AA, Wang X, Lu Z, Goldsmith J, Le XF, Grandjean G, Bartholomeusz G, Broom B, Bast RC. Modulating microtubule stability enhances the cytotoxic response of cancer cells to Paclitaxel. Cancer Res. 2011 Sep 1;71(17):5806-17.

Ahonen TJ, Xie J, LeBaron MJ, Zhu J, Nurmi M, Alanen K, Rui H, Nevalainen MT. Inhibition of transcription factor Stat5 induces cell death of human prostate cancer cells. J Biol Chem. 2003 Jul 18;278(29):27287-92.

Atshaves BP, Storey SM, Petrescu A, Greenberg CC, Lyuksyutova OI, Smith R, Schroeder F. Expression of fatty acid binding proteins inhibits lipid accumulation and alters toxicity in L cell fibroblasts. Am J Physiol Cell Physiol. 2002 Sep;283(3):C688-703.

Balan M, Pal S. A novel CXCR3-B chemokine receptor-induced growth-inhibitory signal in cancer cells is mediated through the regulation of Bach-1 protein and Nrf2 protein nuclear translocation. J Biol Chem. 2014 Feb 7;289(6):3126-37.

Bandyopadhyay S, Pai SK, Watabe M, Gross SC, Hirota S, Hosobe S, Tsukada T, Miura K, Saito K, Markwell SJ, Wang Y, Huggenvik J, Pauza ME, Iiizumi M, Watabe K. FAS expression inversely correlates with PTEN level in prostate cancer and a PI 3-kinase inhibitor synergizes with FAS siRNA to induce apoptosis. Oncogene. 2005 Aug 11;24(34):5389-95.

Behbod F, Nagy ZS, Stepkowski SM, Karras J, Johnson CR, Jarvis WD, Kirken RA. Specific inhibition of Stat5a/b promotes apoptosis of IL-2-responsive primary and tumor-derived lymphoid cells. J Immunol. 2003 Oct 15;171(8):3919-27.

Bond M, Murphy G, Bennett MR, Amour A, Knauper V, Newby AC, Baker AH. Localization of the death domain of tissue inhibitor of metalloproteinase-3 to the N terminus. Metalloproteinase inhibition is associated with proapoptotic activity. J Biol Chem. 2000 Dec 29;275(52):41358-63.

Bordone L, Campbell C. DNA ligase III is degraded by calpain during cell death induced by DNA-damaging agents. J Biol Chem. 2002 Jul 19;277(29):26673-80.

Campagnolo L, Costanza G, Francesconi A, Arcuri G, Moscatelli I, Orlandi A. Sortilin expression is essential for pro-nerve growth factor-induced apoptosis of rat vascular smooth muscle cells. PLoS One. 2014;9(1):e84969. Epub 2014 Jan 3.

Carlson H, Ota S, Song Y, Chen Y, Hurlin PJ. Tbx3 impinges on the p53 pathway to suppress apoptosis, facilitate cell transformation and block myogenic differentiation. Oncogene. 2002 May 30;21(24):3827-35.

Casetti L, Martin-Lannerée S, Najjar I, Plo I, Augé S, Roy L, Chomel JC, Lauret E, Turhan AG, Dusanter-Fourt I. Differential contributions of STAT5A and STAT5B to stress protection and tyrosine kinase inhibitor resistance of chronic myeloid leukemia stem/progenitor cells. Cancer Res. 2013 Apr 1;73(7):2052-8.

Chambers JW, Pachori A, Howard S, Iqbal S, LoGrasso PV. Inhibition of JNK mitochondrial localization and signaling is protective against ischemia/reperfusion injury in rats. J Biol Chem. 2013 Feb 8;288(6):4000-11.

Cheng Z, Zhao H, Ze Y, Su J, Li B, Sheng L, Zhu L, Guan N, Gui S, Sang X, Zhao X, Sun Q, Wang L, Cheng J, Hu R, Hong F. Gene-expression changes in cerium chloride-induced injury of mouse hippocampus. PLoS One. 2013;8(4):e60092.

Cluskey S, Ramsden DB. Mechanisms of neurodegeneration in amyotrophic lateral sclerosis. Mol Pathol. 2001 Dec;54(6):386-92.

Csóka B, Németh ZH, Rosenberger P, Eltzschig HK, Spolarics Z, Pacher P, Selmeczy Z, Koscsó B, Himer L, Vizi ES, Blackburn MR, Deitch EA, Haskó G. A2B adenosine receptors protect against sepsis-induced mortality by dampening excessive inflammation. J Immunol. 2010 Jul 1;185(1):542-50.

Cui H, Darmanin S, Natsuisaka M, Kondo T, Asaka M, Shindoh M, Higashino F, Hamuro J, Okada F, Kobayashi M, Nakagawa K, Koide H, Kobayashi M. Enhanced expression of asparagine synthetase under glucose-deprived conditions protects pancreatic cancer cells from apoptosis induced by glucose deprivation and cisplatin. Cancer Res. 2007 Apr 1;67(7):3345-55.

Das M, Scappini E, Martin NP, Wong KA, Dunn S, Chen YJ, Miller SL, Domin J, O'Bryan JP. Regulation of neuron survival through an intersectin-phosphoinositide 3'-kinase C2beta-AKT pathway. Mol Cell Biol. 2007 Nov;27(22):7906-17.

Deng S, Yang Y, Han Y, Li X, Wang X, Li X, Zhang Z, Wang Y. UCP2 inhibits ROS-mediated apoptosis in A549 under hypoxic conditions. PLoS One. 2012;7(1):e30714

Diano S, Matthews RT, Patrylo P, Yang L, Beal MF, Barnstable CJ, Horvath TL. Uncoupling protein 2 prevents neuronal death including that occurring during seizures: a mechanism for preconditioning. Endocrinology. 2003 Nov;144(11):5014-21. Epub 2003 Aug 21.

Drynda A, Quax PH, Neumann M, van der Laan WH, Pap G, Drynda S, Meinecke I, Kekow J, Neumann W, Huizinga TW, Naumann M, König W, Pap T. Gene transfer of tissue inhibitor of metalloproteinases-3 reverses the inhibitory effects of TNF-alpha on Fas-induced apoptosis in rheumatoid arthritis synovial fibroblasts. J Immunol. 2005 May 15;174(10):6524-31.

Fernandez-Zapico ME, Lomberk GA, Tsuji S, DeMars CJ, Bardsley MR, Lin YH, Almada LL, Han JJ, Mukhopadhyay D, Ordog T, Buttar NS, Urrutia R. A functional family-wide screening of SP/KLF proteins identifies a subset of suppressors of KRAS-mediated cell growth. Biochem J. 2011 Apr 15;435(2):529-37.

Fischer OM, Hart S, Gschwind A, Prenzel N, Ullrich A. Oxidative and osmotic stress signaling in tumor cells is mediated by ADAM proteases and heparin-binding epidermal growth factor. Mol Cell Biol. 2004 Jun;24(12):5172-83.

Gao Y, Lei Z, Lu C, Roisen FJ, El-Mallakh RS. Effect of ionic stress on apoptosis and the expression of TRPM2 in human olfactory neuroepithelial-derived progenitors. World J Biol Psychiatry. 2010 Dec;11(8):972-84.

Ghoreschi K, Laurence A, O'Shea JJ. Janus kinases in immune cell signaling. Immunol Rev. 2009 Mar;228(1):273-87.

Giambarella U, Yamatsuji T, Okamoto T, Matsui T, Ikezu T, Murayama Y, Levine MA, Katz A, Gautam N, Nishimoto I. G protein betagamma complex-mediated apoptosis by familial Alzheimer's disease mutant of APP. EMBO J. 1997 Aug 15;16(16):4897-907.

Ham H, Guerrier S, Kim J, Schoon RA, Anderson EL, Hamann MJ, Lou Z, Billadeau DD. Dedicator of cytokinesis 8 interacts with talin and Wiskott-Aldrich syndrome protein to regulate NK cell cytotoxicity. J Immunol. 2013 Apr 1;190(7):3661-9.

Hou M, Cui J, Liu J, Liu F, Jiang R, Liu K, Wang Y, Yin L, Liu W, Yu B. Angiopoietin-like 4 confers resistance to hypoxia/serum deprivation-induced apoptosis through PI3K/Akt and ERK1/2 signaling pathways in mesenchymal stem cells. PLoS One. 2014;9(1):e85808.

Hung LY, Chen HL, Chang CW, Li BR, Tang TK. Identification of a novel microtubule-destabilizing motif in CPAP that binds to tubulin heterodimers and inhibits microtubule assembly. Mol Biol Cell. 2004 Jun;15(6):2697-706.

Ito A, Asamoto M, Hokaiwado N, Takahashi S, Shirai T. Tbx3 expression is related to apoptosis and cell proliferation in rat bladder both hyperplastic epithelial cells and carcinoma cells. Cancer Lett. 2005 Feb 28;219(1):105-12.

Jeon HK, Jin HS, Lee DH, Choi WS, Moon CK, Oh YJ, Lee TH. Proteome analysis associated with cadmium adaptation in U937 cells: identification of calbindin-D28k as a secondary cadmium-responsive protein that confers resistance to cadmium-induced apoptosis. J Biol Chem. 2004 Jul 23;279(30):31575-83.

Jiang L, Allagnat F, Nguidjoe E, Kamagate A, Pachera N, Vanderwinden JM, Brini M, Carafoli E, Eizirik DL, Cardozo AK, Herchuelz A. Plasma membrane Ca2+-ATPase overexpression depletes both mitochondrial and endoplasmic reticulum Ca2+ stores and triggers apoptosis in insulin-secreting BRIN-BD11 cells. J Biol Chem. 2010 Oct 1;285(40):30634-43.

Jounaidi Y, Waxman DJ. Combination of the bioreductive drug tirapazamine with the chemotherapeutic prodrug cyclophosphamide for P450/P450-reductase-based cancer gene therapy. Cancer Res. 2000 Jul 15;60(14):3761-9.

Kim I, Kim HG, Kim H, Kim HH, Park SK, Uhm CS, Lee ZH, Koh GY. Hepatic expression, synthesis and secretion of a novel fibrinogen/angiopoietin-related protein that prevents endothelial-cell apoptosis. Biochem J. 2000 Mar 15;346 Pt 3:603-10.

Kim C, Wong J, Wen J, Wang S, Wang C, Spiering S, Kan NG, Forcales S, Puri PL, Leone TC, Marine JE, Calkins H, Kelly DP, Judge DP, Chen HS. Studying arrhythmogenic right ventricular dysplasia with patient-specific iPSCs. Nature. 2013 Feb 7;494(7435):105-10.

Kriska T, Levchenko VV, Korytowski W, Atshaves BP, Schroeder F, Girotti AW. Intracellular dissemination of peroxidative stress. Internalization, transport, and lethal targeting of a cholesterol hydroperoxide species by sterol carrier protein-2-overexpressing hepatoma cells. J Biol Chem. 2006 Aug 18;281(33):23643-51.

Kung HN, Weng TY, Liu YL, Lu KS, Chau YP. Sulindac compounds facilitate the cytotoxicity of β-lapachone by up-regulation of NAD(P)H quinone oxidoreductase in human lung cancer cells. PLoS One. 2014;9(2):e88122. Epub 2014 Feb 5.

Lewis ND, Asim M, Barry DP, de Sablet T, Singh K, Piazuelo MB, Gobert AP, Chaturvedi R, Wilson KT. Immune evasion by Helicobacter pylori is mediated by induction of macrophage arginase II. J Immunol. 2011 Mar 15;186(6):3632-41.

Liu Y, Mei C, Sun L, Li X, Liu M, Wang L, Li Z, Yin P, Zhao C, Shi Y, Qiu S, Fan J, Zha X. The PI3K-Akt pathway regulates calpain 6 expression, proliferation, and apoptosis. Cell Signal. 2011 May;23(5):827-36.

Loinard C, Zouggari Y, Rueda P, Ramkhelawon B, Cochain C, Vilar J, Récalde A, Richart A, Charue D, Duriez M, Mori M, Arenzana-Seisdedos F, Lévy BI, Heymes C, Silvestre JS. C/EBP homologous protein-10 (CHOP-10) limits postnatal neovascularization through control of endothelial nitric oxide synthase gene expression. Circulation. 2012 Feb 28;125(8):1014-26. Epub 2012 Jan 20.

Long JS, Crighton D, O'Prey J, Mackay G, Zheng L, Palmer TM, Gottlieb E, Ryan KM. Extracellular adenosine sensing-a metabolic cell death priming mechanism downstream of p53. Mol Cell. 2013 May 9;50(3):394-406.

Lovat PE, Oliverio S, Ranalli M, Corazzari M, Rodolfo C, Bernassola F, Aughton K, Maccarrone M, Hewson QD, Pearson AD, Melino G, Piacentini M, Redfern CP. GADD153 and 12-lipoxygenase mediate fenretinide-induced apoptosis of neuroblastoma. Cancer Res. 2002 Sep 15;62(18):5158-67.

Méndez-Lucas A, Hyroššová P, Novellasdemunt L, Viñals F, Perales JC. Mitochondrial phosphoenolpyruvate carboxykinase (PEPCK-M) is a pro-survival, endoplasmic reticulum (ER) stress response gene involved in tumor cell adaptation to nutrient availability. J Biol Chem. 2014 Aug 8;289(32):22090-102. Epub 2014 Jun 27.

Mittoux V, Ouary S, Monville C, Lisovoski F, Poyot T, Conde F, Escartin C, Robichon R, Brouillet E, Peschanski M, Hantraye P. Corticostriatopallidal neuroprotection by adenovirus-mediated ciliary neurotrophic factor gene transfer in a rat model of progressive striatal degeneration. J Neurosci. 2002 Jun 1;22(11):4478-86.

Moretta A, Marcenaro E, Parolini S, Ferlazzo G, Moretta L. NK cells at the interface between innate and adaptive immunity. Cell Death Differ. 2008 Feb;15(2):226-33.

Niessen F, Schaffner F, Furlan-Freguia C, Pawlinski R, Bhattacharjee G, Chun J, Derian CK, Andrade-Gordon P, Rosen H, Ruf W. Dendritic cell PAR1-S1P3 signalling couples coagulation and inflammation. Nature. 2008 Apr 3;452(7187):654-8.

Nübel T, Preobraschenski J, Tuncay H, Weiss T, Kuhn S, Ladwein M, Langbein L, Zöller M. Claudin-7 regulates EpCAM-mediated functions in tumor progression. Mol Cancer Res. 2009 Mar;7(3):285-99.

Nykjaer A, Lee R, Teng KK, Jansen P, Madsen P, Nielsen MS, Jacobsen C, Kliemannel M, Schwarz E, Willnow TE, Hempstead BL, Petersen CM. Sortilin is essential for proNGF-induced neuronal cell death. Nature. 2004 Feb 26;427(6977):843-8.

Passer BJ, Nancy-Portebois V, Amzallag N, Prieur S, Cans C, Roborel de Climens A, Fiucci G, Bouvard V, Tuynder M, Susini L, Morchoisne S, Crible V, Lespagnol A, Dausset J, Oren M, Amson R, Telerman A. The p53-inducible TSAP6 gene product regulates apoptosis and the cell cycle and interacts with Nix and the Myt1 kinase. Proc Natl Acad Sci U S A. 2003 Mar 4;100(5):2284-9.

Peng Y, Zhang J, Xu H, He J, Ying X, Wang Y. Neuroprotective effect of protease-activated receptor-2 in the hypoxia-induced apoptosis of rat RGC-5 cells. J Mol Neurosci. 2013 May;50(1):98-108.

Persengiev SP, Devireddy LR, Green MR. Inhibition of apoptosis by ATFx: a novel role for a member of the ATF/CREB family of mammalian bZIP transcription factors. Genes Dev. 2002 Jul 15;16(14):1806-14.

Piccoli R, Di Gaetano S, De Lorenzo C, Grauso M, Monaco C, Spalletti-Cernia D, Laccetti P, Cinátl J, Matousek J, D'Alessio G. A dimeric mutant of human pancreatic ribonuclease with selective cytotoxicity toward malignant cells. Proc Natl Acad Sci U S A. 1999 Jul 6;96(14):7768-73.

Pillai JB, Isbatan A, Imai S, Gupta MP. Poly(ADP-ribose) polymerase-1-dependent cardiac myocyte cell death during heart failure is mediated by NAD+ depletion and reduced Sir2alpha deacetylase activity. J Biol Chem. 2005 Dec 30;280(52):43121-30.

Pliyev BK, Ivanova AV, Savchenko VG. Extracellular NAD(+) inhibits human neutrophil apoptosis. Apoptosis. 2014 Apr;19(4):581-93.

Pilzer I, Gozes I. VIP provides cellular protection through a specific splice variant of the PACAP receptor: a new neuroprotection target. Peptides. 2006 Nov;27(11):2867-76.

Predescu SA, Predescu DN, Knezevic I, Klein IK, Malik AB. Intersectin-1s regulates the mitochondrial apoptotic pathway in endothelial cells. J Biol Chem. 2007 Jun 8;282(23):17166-78.

Raasi S, Schmidtke G, Groettrup M. The ubiquitin-like protein FAT10 forms covalent conjugates and induces apoptosis. J Biol Chem. 2001 Sep 21;276(38):35334-43.

Rommel C, Camps M, Ji H. PI3K delta and PI3K gamma: partners in crime in inflammation in rheumatoid arthritis and beyond? Nat Rev Immunol. 2007 Mar;7(3):191-201. Epub 2007 Feb 9.

Rüttiger L, Sausbier M, Zimmermann U, Winter H, Braig C, Engel J, Knirsch M, Arntz C, Langer P, Hirt B, Müller M, Köpschall I, Pfister M, Münkner S, Rohbock K, Pfaff I, Rüsch A, Ruth P, Knipper M. Deletion of the Ca2+-activated potassium (BK) alpha-subunit but not the BKbeta1-subunit leads to progressive hearing loss. Proc Natl Acad Sci U S A. 2004 Aug 31;101(35):12922-7.

Ryu KY, Maehr R, Gilchrist CA, Long MA, Bouley DM, Mueller B, Ploegh HL, Kopito RR. The mouse polyubiquitin gene UbC is essential for fetal liver development, cell-cycle progression and stress tolerance. EMBO J. 2007 Jun 6;26(11):2693-706. Epub 2007 May 10.

Ryu J, Park SG, Lee PY, Cho S, Lee do H, Kim GH, Kim JH, Park BC. Dimerization of pro-oncogenic protein Anterior Gradient 2 is required for the interaction with BiP/GRP78. Biochem Biophys Res Commun. 2013 Jan 11;430(2):610-5. Epub 2012 Dec 4.

Sahr KE, Lambert AJ, Ciciotte SL, Mohandas N, Peters LL. Targeted deletion of the gamma-adducin gene (Add3) in mice reveals differences in alpha-adducin interactions in erythroid and nonerythroid cells. Am J Hematol. 2009 Jun;84(6):354-61.

Sasaki T, Irie-Sasaki J, Jones RG, Oliveira-dos-Santos AJ, Stanford WL, Bolon B, Wakeham A, Itie A, Bouchard D, Kozieradzki I, Joza N, Mak TW, Ohashi PS, Suzuki A, Penninger JM. Function of PI3Kgamma in thymocyte development, T cell activation, and neutrophil migration. Science. 2000 Feb 11;287(5455):1040-6.

Sengupta A, Arnett J, Dunn S, Williams DA, Cancelas JA. Rac2 GTPase deficiency depletes BCR-ABL+ leukemic stem cells and progenitors in vivo. Blood. 2010 Jul 8;116(1):81-4.

Shimizu K, Takahama S, Endo Y, Sawasaki T. Stress-inducible caspase substrate TRB3 promotes nuclear translocation of procaspase-3. PLoS One. 2012;7(8):e42721. Epub 2012 Aug 9.

Shiragami R, Murata S, Kosugi C, Tezuka T, Yamazaki M, Hirano A, Yoshimura Y, Suzuki M, Shuto K, Koda K. Enhanced antitumor activity of cerulenin combined with oxaliplatin in human colon cancer cells. Int J Oncol. 2013 Aug;43(2):431-8.

Steiner MS, Zhang X, Wang Y, Lu Y. Growth inhibition of prostate cancer by an adenovirus expressing a novel tumor suppressor gene, pHyde. Cancer Res. 2000 Aug 15;60(16):4419-25.

Sumitomo M, Milowsky MI, Shen R, Navarro D, Dai J, Asano T, Hayakawa M, Nanus DM. Neutral endopeptidase inhibits neuropeptide-mediated transactivation of the insulin-like growth factor receptor-Akt cell survival pathway. Cancer Res. 2001 Apr 15;61(8):3294-8.

Sun Z, Zhang J, Zhang J, Chen C, Du Q, Chang L, Cao C, Zheng M, Garcia-Barrio MT, Chen YE, Xiao RP, Mao J, Zhu X. Rad GTPase induces cardiomyocyte apoptosis through the activation of p38 mitogen-activated protein kinase. Biochem Biophys Res Commun. 2011 May 27;409(1):52-7.

Ueno M, Itoh M, Kong L, Sugihara K, Asano M, Takakura N. PSF1 is essential for early embryogenesis in mice. Mol Cell Biol. 2005 Dec;25(23):10528-32.

Vaidyanathan G, Cismowski MJ, Wang G, Vincent TS, Brown KD, Lanier SM. The Ras-related protein AGS1/RASD1 suppresses cell growth. Oncogene. 2004 Jul 29;23(34):5858-63

Wang H, Maechler P, Antinozzi PA, Herrero L, Hagenfeldt-Johansson KA, Bjorklund A, Wollheim CB. The transcription factor SREBP-1c is instrumental in the development of beta-cell dysfunction. J Biol Chem. 2003 May 9;278(19):16622-9.

Welch C, Santra MK, El-Assaad W, Zhu X, Huber WE, Keys RA, Teodoro JG, Green MR. Identification of a protein, G0S2, that lacks Bcl-2 homology domains and interacts with and antagonizes Bcl-2. Cancer Res. 2009 Sep 1;69(17):6782-9. Epub 2009 Aug 25.

Wilson HL, Wilson SA, Surprenant A, North RA. Epithelial membrane proteins induce membrane blebbing and interact with the P2X7 receptor C terminus. J Biol Chem. 2002 Sep 13;277(37):34017-23.

Woo IS, Eun SY, Jang HS, Kang ES, Kim GH, Kim HJ, Lee JH, Chang KC, Kim JH, Han CW, Seo HG. Identification of ADP-ribosylation factor 4 as a suppressor of N-(4-hydroxyphenyl)retinamide-induced cell death. Cancer Lett. 2009 Apr 8;276(1):53-60. Epub 2008 Nov 28.

Wu L, Ma CA, Zhao Y, Jain A. Aurora B interacts with NIR-p53, leading to p53 phosphorylation in its DNA-binding domain and subsequent functional suppression. J Biol Chem. 2011 Jan 21;286(3):2236-44.

Wu M, Xu LG, Zhai Z, Shu HB. SINK is a p65-interacting negative regulator of NF-kappaB-dependent transcription. J Biol Chem. 2003 Jul 18;278(29):27072-9. Epub 2003 May 7.

Ye J, Wang S, Leonard SS, Sun Y, Butterworth L, Antonini J, Ding M, Rojanasakul Y, Vallyathan V, Castranova V, Shi X. Role of reactive oxygen species and p53 in chromium(VI)-induced apoptosis. J Biol Chem. 1999 Dec 3;274(49):34974-80.

Zhang DS, Liu ZY, Li YJ, Sun ZL. NQO1 involves in the imine bond reduction of sanguinarine and recombinant adeno-associated virus mediated NQO1 overexpression decreases sanguinarine-induced cytotoxicity in rat BRL cells. Toxicol Lett. 2014 Feb 10;225(1):119-29.

Zhang D, Zhang J, Jiang X, Li X, Wang Y, Ma J, Jiang H. Heparin-binding epidermal growth factor-like growth factor: a hepatic stellate cell proliferation inducer via ErbB receptors. J Gastroenterol Hepatol. 2014 Mar;29(3):623-32.

Zhao Y, Altman BJ, Coloff JL, Herman CE, Jacobs SR, Wieman HL, Wofford JA, Dimascio LN, Ilkayeva O, Kelekar A, Reya T, Rathmell JC. Glycogen synthase kinase 3alpha and 3beta mediate a glucose-sensitive antiapoptotic signaling pathway to stabilize Mcl-1. Mol Cell Biol. 2007 Jun;27(12):4328-39.

Zhong Y, Cantwell A, Dube PH. Transforming growth factor beta and CD25 are important for controlling systemic dissemination following Yersinia enterocolitica infection of the gut. Infect Immun. 2010 Sep;78(9):3716-25.
