# Supplementary material for: Transcriptome adaptation of the bovine mammary gland to diets rich in unsaturated fatty acids shows greater impact of linseed oil over safflower oil on gene expression and metabolic pathways
Source: BMC Genomics. 2016 Feb 9;17:104. doi: 10.1186/s12864-016-2423-x (PMC4748538; doi:10.1186/s12864-016-2423-x)
Supplement: Additional file 17: — Differentially expressed genes implicated in apoptosis of cows in LSO treatment as compared to the same cows on the control diet. Expression direction of several genes predicted to decrease apoptosis. (DOCX 35 kb) [file 12864_2016_2423_MOESM17_ESM.docx]

**Additional file 17**

**Differentially expressed genes implicated in apoptosis between cows on control diets and same cows supplemented with linseed oil for 28 days. Apoptosis with a positive Z-score (0.039, P-value 1.9E-05) indicates a small degree of activation.**

| Genes in dataset | Prediction (based on expression direction) | Fold change | Literature findings (references) |
| --- | --- | --- | --- |
| RNASE1 | Increased | 3.182 | Increases (Piccoli et al. 1999) |
| CALB1 | Decreased | 2.990 | Decreases (Turneret al. 2004, Jeon et al. 2004) |
| KLF11 (TIEG2) | Increased | 2.592 | Increases (Fernandez-Zapico et al. 2011) |
| TRIB3 | Increased | 2.456 | Increases (Shimizu et al. 2012, Wu et al. 2003) |
| UCP2 | Decreased | 2.256 | Decreases (Degasperi et al.2008, Deng et al. 2012) |
| ANGPTL4 | Decreased | 2.166 | Decreases (Hou et al. 2014, Kim et al. 2000) |
| HBEGF | Decreased | 2.006 | Decreases (Fischer et al. 2004, Zhang et al. 2014) |
| ATF5 | Decreased | 1.820 | Decreases (Persengiev et al. 2002) |
| PIK3CG | Decreased | 1.788 | Decreases (Rommel et al. 2007, Sasaki et al. 2000) |
| RAPGEF4 | Decreased | 1.657 | Decreases (Ahmed et al. 2011) |
| DDIT3 | Increased | 1.553 | Increases (Lovat et al. 2002, Loinard et al. 2012) |
| G0S2 | Increased | 1.535 | Increases (Welch et al. 2009) |
| ASNS | Decreased | 1.495 | Decreases (Cui et al. 2007) |
| ARF4 | Decreased | 1.478 | Decreases (Woo et al. 2008) |
| ATP2B2 | Affected | 1.435 | Affects (Jiang et al. 2010) |
| TBX3 | Decreased | 1.396 | Decreases (Carlson et al. 2002, Ito et al. 2005) |
| GSR | Increased | 1.384 | Increases (Ye et al. 1999) |
| SORT1 | Increased | 1.368 | Increases (Nykjaer et al. 2004, Campagnolo et al. 2014) |
| NQO1 | Increased | 1.358 | Increases (Jamshidi et al. 2012, Zhang et al. 2014) |
| KCNMA1 | Affected | 1.336 | Affects (Rüttiger et al. 2004) |
| GNG2 | Increased | 1.309 | Increases (Giambarella et al. 1997) |
| NOC2L | Decreased | 1.280 | Decreases (Wu et al. 2011) |
| MME | Decreased | 1.265 | Decreases (Sumitomo et al. 2001) |
| GINS1 | Affected | 1.250 | Affects (Ueno et al. 2005) |
| SLC2A1 | Increased | -1.252 | Decreases (Zhao et al. 2007) |
| CLDN7 | Increased | -1.279 | Decreases (Nübel et al. 2007) |
| STEAP3 | Decreased | -1.279 | Increases (Steiner et al. 2000, Passer et al. 2003) |
| BACH1 | Decreased | -1.288 | Increases (Balan and Pal, 2014) |
| STAT5A | Increased | -1.317 | Decreases (Casetti et al. 2013, Ahonen et al. 2003) |
| F2RL1 | Decreased | -1.320 | Increases (Peng et al. 2013) |
| TIMP3 | Decreased | -1.328 | Increases (Bond et al. 2000, Drynda et al. 2005) |
| STAT5B | Increased | -1.328 | Decreases (Behbod et al. 2003, Casetti et al. 2013) |
| ITSN1 | Increased | -1.329 | Decreases (Das et al. 2007, Predescu et al. 2007) |
| F2R | Decreased | -1.375 | Increases (Mosnier et al. 2007) |
| PKP2 | Affected | -1.391 | Affects (Kim et al. 2013) |
| RAC2 | Increased | -1.420 | Decreases (Sengupta et al. 2010) |
| CSRNP1 | Affected | -1.433 | Affects (Cheng et al. 2013) |
| TRPM2 | Affected | -1.458 | Affects (Gao et al. 2010) |
| FASN | Increased | -1.520 | Decreases (Shiragami et al. 2013, Bandyopadhyay et al. 2005) |
| SREBF1 | Decreased | -1.645 | Increases (Wang et al. 2003) |
| RRAD | Decreased | -1.673 | Increases (Sun et al. 2011) |
| RASD1 | Decreased | -1.719 | Increases (Vaidyanathan et al. 2004) |
| UBD | Decreased | -1.757 | Increases (Raasi et al. 2001) |
| ADORA2B | Affected | -1.804 | Affects (Long et al. 2013, Csóka et al. 2010) |
| CENPJ | Increased | -1.855 | Decreases (Hung et al. 2004) |
| CAPN6 | Increased | -1.928 | Decreases (Liu et al. 2011) |
| ARG2 | Increased | -1.942 | Decreases (Lewis et al. 2011) |
| NAD+ (C3) | Decreased | -1.942 | Increases (Pliyev et al. 2014) |

^1^Expression direction of 22genes in this study decreases cell death and expressed direction of 19 genes increases cell death

**References**

Ahmed AA, Wang X, Lu Z, Goldsmith J, Le XF, Grandjean G, Bartholomeusz G, Broom B, Bast RC. Modulating microtubule stability enhances the cytotoxic response of cancer cells to Paclitaxel. Cancer Res. 2011 Sep 1;71(17):5806-17.

Ahonen TJ, Xie J, LeBaron MJ, Zhu J, Nurmi M, Alanen K, Rui H, Nevalainen MT. Inhibition of transcription factor Stat5 induces cell death of human prostate cancer cells. J Biol Chem. 2003 Jul 18;278(29):27287-92.

Balan M, Pal S. A novel CXCR3-B chemokine receptor-induced growth-inhibitory signal in cancer cells is mediated through the regulation of Bach-1 protein and Nrf2 protein nuclear translocation. J Biol Chem. 2014 Feb 7;289(6):3126-37.

Bandyopadhyay S, Pai SK, Watabe M, Gross SC, Hirota S, Hosobe S, Tsukada T, Miura K, Saito K, Markwell SJ, Wang Y, Huggenvik J, Pauza ME, Iiizumi M, Watabe K. FAS expression inversely correlates with PTEN level in prostate cancer and a PI 3-kinase inhibitor synergizes with FAS siRNA to induce apoptosis. Oncogene. 2005 Aug 11;24(34):5389-95.

Behbod F, Nagy ZS, Stepkowski SM, Karras J, Johnson CR, Jarvis WD, Kirken RA. Specific inhibition of Stat5a/b promotes apoptosis of IL-2-responsive primary and tumor-derived lymphoid cells. J Immunol. 2003 Oct 15;171(8):3919-27.

Bond M, Murphy G, Bennett MR, Amour A, Knauper V, Newby AC, Baker AH. Localization of the death domain of tissue inhibitor of metalloproteinase-3 to the N terminus. Metalloproteinase inhibition is associated with proapoptotic activity. J Biol Chem. 2000 Dec 29;275(52):41358-63.

Campagnolo L, Costanza G, Francesconi A, Arcuri G, Moscatelli I, Orlandi A. Sortilin expression is essential for pro-nerve growth factor-induced apoptosis of rat vascular smooth muscle cells. PLoS One. 2014;9(1):e84969. Epub 2014 Jan 3.

Carlson H, Ota S, Song Y, Chen Y, Hurlin PJ. Tbx3 impinges on the p53 pathway to suppress apoptosis, facilitate cell transformation and block myogenic differentiation. Oncogene. 2002 May 30;21(24):3827-35.

Casetti L, Martin-Lannerée S, Najjar I, Plo I, Augé S, Roy L, Chomel JC, Lauret E, Turhan AG, Dusanter-Fourt I. Differential contributions of STAT5A and STAT5B to stress protection and tyrosine kinase inhibitor resistance of chronic myeloid leukemia stem/progenitor cells. Cancer Res. 2013 Apr 1;73(7):2052-8.

Cheng Z, Zhao H, Ze Y, Su J, Li B, Sheng L, Zhu L, Guan N, Gui S, Sang X, Zhao X, Sun Q, Wang L, Cheng J, Hu R, Hong F. Gene-expression changes in cerium chloride-induced injury of mouse hippocampus. PLoS One. 2013;8(4):e60092.

Csóka B, Németh ZH, Rosenberger P, Eltzschig HK, Spolarics Z, Pacher P, Selmeczy Z, Koscsó B, Himer L, Vizi ES, Blackburn MR, Deitch EA, Haskó G. A2B adenosine receptors protect against sepsis-induced mortality by dampening excessive inflammation. J Immunol. 2010 Jul 1;185(1):542-50.

Cui H, Darmanin S, Natsuisaka M, Kondo T, Asaka M, Shindoh M, Higashino F, Hamuro J, Okada F, Kobayashi M, Nakagawa K, Koide H, Kobayashi M. Enhanced expression of asparagine synthetase under glucose-deprived conditions protects pancreatic cancer cells from apoptosis induced by glucose deprivation and cisplatin. Cancer Res. 2007 Apr 1;67(7):3345-55.

Das M, Scappini E, Martin NP, Wong KA, Dunn S, Chen YJ, Miller SL, Domin J, O'Bryan JP. Regulation of neuron survival through an intersectin-phosphoinositide 3'-kinase C2beta-AKT pathway. Mol Cell Biol. 2007 Nov;27(22):7906-17.

Degasperi GR, Romanatto T, Denis RG, Araújo EP, Moraes JC, Inada NM, Vercesi AE, Velloso LA. UCP2 protects hypothalamic cells from TNF-alpha-induced damage. FEBS Lett. 2008 Sep 3;582(20):3103-10.

Deng S, Yang Y, Han Y, Li X, Wang X, Li X, Zhang Z, Wang Y. UCP2 inhibits ROS-mediated apoptosis in A549 under hypoxic conditions. PLoS One. 2012;7(1):e30714

Drynda A, Quax PH, Neumann M, van der Laan WH, Pap G, Drynda S, Meinecke I, Kekow J, Neumann W, Huizinga TW, Naumann M, König W, Pap T. Gene transfer of tissue inhibitor of metalloproteinases-3 reverses the inhibitory effects of TNF-alpha on Fas-induced apoptosis in rheumatoid arthritis synovial fibroblasts. J Immunol. 2005 May 15;174(10):6524-31.

Fernandez-Zapico ME, Lomberk GA, Tsuji S, DeMars CJ, Bardsley MR, Lin YH, Almada LL, Han JJ, Mukhopadhyay D, Ordog T, Buttar NS, Urrutia R. A functional family-wide screening of SP/KLF proteins identifies a subset of suppressors of KRAS-mediated cell growth. Biochem J. 2011 Apr 15;435(2):529-37.

Fischer OM, Hart S, Gschwind A, Prenzel N, Ullrich A. Oxidative and osmotic stress signaling in tumor cells is mediated by ADAM proteases and heparin-binding epidermal growth factor. Mol Cell Biol. 2004 Jun;24(12):5172-83.

Gao Y, Lei Z, Lu C, Roisen FJ, El-Mallakh RS. Effect of ionic stress on apoptosis and the expression of TRPM2 in human olfactory neuroepithelial-derived progenitors. World J Biol Psychiatry. 2010 Dec;11(8):972-84.

Giambarella U, Yamatsuji T, Okamoto T, Matsui T, Ikezu T, Murayama Y, Levine MA, Katz A, Gautam N, Nishimoto I. G protein betagamma complex-mediated apoptosis by familial Alzheimer's disease mutant of APP. EMBO J. 1997 Aug 15;16(16):4897-907.

Hou M, Cui J, Liu J, Liu F, Jiang R, Liu K, Wang Y, Yin L, Liu W, Yu B. Angiopoietin-like 4 confers resistance to hypoxia/serum deprivation-induced apoptosis through PI3K/Akt and ERK1/2 signaling pathways in mesenchymal stem cells. PLoS One. 2014;9(1):e85808.

Hung LY, Chen HL, Chang CW, Li BR, Tang TK. Identification of a novel microtubule-destabilizing motif in CPAP that binds to tubulin heterodimers and inhibits microtubule assembly. Mol Biol Cell. 2004 Jun;15(6):2697-706.

Ito A, Asamoto M, Hokaiwado N, Takahashi S, Shirai T. Tbx3 expression is related to apoptosis and cell proliferation in rat bladder both hyperplastic epithelial cells and carcinoma cells. Cancer Lett. 2005 Feb 28;219(1):105-12.

Jamshidi M, Bartkova J, Greco D, Tommiska J, Fagerholm R, Aittomäki K, Mattson J, Villman K, Vrtel R, Lukas J, Heikkilä P, Blomqvist C, Bartek J, Nevanlinna H. NQO1 expression correlates inversely with NFκB activation in human breast cancer. Breast Cancer Res Treat. 2012 Apr;132(3):955-68.

Jiang L, Allagnat F, Nguidjoe E, Kamagate A, Pachera N, Vanderwinden JM, Brini M, Carafoli E, Eizirik DL, Cardozo AK, Herchuelz A. Plasma membrane Ca2+-ATPase overexpression depletes both mitochondrial and endoplasmic reticulum Ca2+ stores and triggers apoptosis in insulin-secreting BRIN-BD11 cells. J Biol Chem. 2010 Oct 1;285(40):30634-43.

Kim I, Kim HG, Kim H, Kim HH, Park SK, Uhm CS, Lee ZH, Koh GY. Hepatic expression, synthesis and secretion of a novel fibrinogen/angiopoietin-related protein that prevents endothelial-cell apoptosis. Biochem J. 2000 Mar 15;346 Pt 3:603-10.

Kim C, Wong J, Wen J, Wang S, Wang C, Spiering S, Kan NG, Forcales S, Puri PL, Leone TC, Marine JE, Calkins H, Kelly DP, Judge DP, Chen HS. Studying arrhythmogenic right ventricular dysplasia with patient-specific iPSCs. Nature. 2013 Feb 7;494(7435):105-10.

Lewis ND, Asim M, Barry DP, de Sablet T, Singh K, Piazuelo MB, Gobert AP, Chaturvedi R, Wilson KT. Immune evasion by Helicobacter pylori is mediated by induction of macrophage arginase II. J Immunol. 2011 Mar 15;186(6):3632-41.

Liu Y, Mei C, Sun L, Li X, Liu M, Wang L, Li Z, Yin P, Zhao C, Shi Y, Qiu S, Fan J, Zha X. The PI3K-Akt pathway regulates calpain 6 expression, proliferation, and apoptosis. Cell Signal. 2011 May;23(5):827-36.

Loinard C, Zouggari Y, Rueda P, Ramkhelawon B, Cochain C, Vilar J, Récalde A, Richart A, Charue D, Duriez M, Mori M, Arenzana-Seisdedos F, Lévy BI, Heymes C, Silvestre JS. C/EBP homologous protein-10 (CHOP-10) limits postnatal neovascularization through control of endothelial nitric oxide synthase gene expression. Circulation. 2012 Feb 28;125(8):1014-26. Epub 2012 Jan 20.

Long JS, Crighton D, O'Prey J, Mackay G, Zheng L, Palmer TM, Gottlieb E, Ryan KM. Extracellular adenosine sensing-a metabolic cell death priming mechanism downstream of p53. Mol Cell. 2013 May 9;50(3):394-406.

Lovat PE, Oliverio S, Ranalli M, Corazzari M, Rodolfo C, Bernassola F, Aughton K, Maccarrone M, Hewson QD, Pearson AD, Melino G, Piacentini M, Redfern CP. GADD153 and 12-lipoxygenase mediate fenretinide-induced apoptosis of neuroblastoma. Cancer Res. 2002 Sep 15;62(18):5158-67.

Mosnier LO, Yang XV, Griffin JH. Activated protein C mutant with minimal anticoagulant activity, normal cytoprotective activity, and preservation of thrombin activable fibrinolysis inhibitor-dependent cytoprotective functions. J Biol Chem. 2007 Nov 9;282(45):33022-33.

Nübel T, Preobraschenski J, Tuncay H, Weiss T, Kuhn S, Ladwein M, Langbein L, Zöller M. Claudin-7 regulates EpCAM-mediated functions in tumor progression. Mol Cancer Res. 2009 Mar;7(3):285-99.

Nykjaer A, Lee R, Teng KK, Jansen P, Madsen P, Nielsen MS, Jacobsen C, Kliemannel M, Schwarz E, Willnow TE, Hempstead BL, Petersen CM. Sortilin is essential for proNGF-induced neuronal cell death. Nature. 2004 Feb 26;427(6977):843-8.

Passer BJ, Nancy-Portebois V, Amzallag N, Prieur S, Cans C, Roborel de Climens A, Fiucci G, Bouvard V, Tuynder M, Susini L, Morchoisne S, Crible V, Lespagnol A, Dausset J, Oren M, Amson R, Telerman A. The p53-inducible TSAP6 gene product regulates apoptosis and the cell cycle and interacts with Nix and the Myt1 kinase. Proc Natl Acad Sci U S A. 2003 Mar 4;100(5):2284-9.

Peng Y, Zhang J, Xu H, He J, Ying X, Wang Y. Neuroprotective effect of protease-activated receptor-2 in the hypoxia-induced apoptosis of rat RGC-5 cells. J Mol Neurosci. 2013 May;50(1):98-108.

Persengiev SP, Devireddy LR, Green MR. Inhibition of apoptosis by ATFx: a novel role for a member of the ATF/CREB family of mammalian bZIP transcription factors. Genes Dev. 2002 Jul 15;16(14):1806-14.

Piccoli R, Di Gaetano S, De Lorenzo C, Grauso M, Monaco C, Spalletti-Cernia D, Laccetti P, Cinátl J, Matousek J, D'Alessio G. A dimeric mutant of human pancreatic ribonuclease with selective cytotoxicity toward malignant cells. Proc Natl Acad Sci U S A. 1999 Jul 6;96(14):7768-73.

Pillai JB, Isbatan A, Imai S, Gupta MP. Poly(ADP-ribose) polymerase-1-dependent cardiac myocyte cell death during heart failure is mediated by NAD+ depletion and reduced Sir2alpha deacetylase activity. J Biol Chem. 2005 Dec 30;280(52):43121-30.

Pliyev BK, Ivanova AV, Savchenko VG. Extracellular NAD(+) inhibits human neutrophil apoptosis. Apoptosis. 2014 Apr;19(4):581-93.

Predescu SA, Predescu DN, Knezevic I, Klein IK, Malik AB. Intersectin-1s regulates the mitochondrial apoptotic pathway in endothelial cells. J Biol Chem. 2007 Jun 8;282(23):17166-78.

Raasi S, Schmidtke G, Groettrup M. The ubiquitin-like protein FAT10 forms covalent conjugates and induces apoptosis. J Biol Chem. 2001 Sep 21;276(38):35334-43.

Rommel C, Camps M, Ji H. PI3K delta and PI3K gamma: partners in crime in inflammation in rheumatoid arthritis and beyond? Nat Rev Immunol. 2007 Mar;7(3):191-201. Epub 2007 Feb 9.

Rüttiger L, Sausbier M, Zimmermann U, Winter H, Braig C, Engel J, Knirsch M, Arntz C, Langer P, Hirt B, Müller M, Köpschall I, Pfister M, Münkner S, Rohbock K, Pfaff I, Rüsch A, Ruth P, Knipper M. Deletion of the Ca2+-activated potassium (BK) alpha-subunit but not the BKbeta1-subunit leads to progressive hearing loss. Proc Natl Acad Sci U S A. 2004 Aug 31;101(35):12922-7.

Sahr KE, Lambert AJ, Ciciotte SL, Mohandas N, Peters LL. Targeted deletion of the gamma-adducin gene (Add3) in mice reveals differences in alpha-adducin interactions in erythroid and nonerythroid cells. Am J Hematol. 2009 Jun;84(6):354-61.

Sasaki T, Irie-Sasaki J, Jones RG, Oliveira-dos-Santos AJ, Stanford WL, Bolon B, Wakeham A, Itie A, Bouchard D, Kozieradzki I, Joza N, Mak TW, Ohashi PS, Suzuki A, Penninger JM. Function of PI3Kgamma in thymocyte development, T cell activation, and neutrophil migration. Science. 2000 Feb 11;287(5455):1040-6.

Sengupta A, Arnett J, Dunn S, Williams DA, Cancelas JA. Rac2 GTPase deficiency depletes BCR-ABL+ leukemic stem cells and progenitors in vivo. Blood. 2010 Jul 8;116(1):81-4.

Shimizu K, Takahama S, Endo Y, Sawasaki T. Stress-inducible caspase substrate TRB3 promotes nuclear translocation of procaspase-3. PLoS One. 2012;7(8):e42721. Epub 2012 Aug 9.

Shiragami R, Murata S, Kosugi C, Tezuka T, Yamazaki M, Hirano A, Yoshimura Y, Suzuki M, Shuto K, Koda K. Enhanced antitumor activity of cerulenin combined with oxaliplatin in human colon cancer cells. Int J Oncol. 2013 Aug;43(2):431-8.

Steiner MS, Zhang X, Wang Y, Lu Y. Growth inhibition of prostate cancer by an adenovirus expressing a novel tumor suppressor gene, pHyde. Cancer Res. 2000 Aug 15;60(16):4419-25.

Sumitomo M, Milowsky MI, Shen R, Navarro D, Dai J, Asano T, Hayakawa M, Nanus DM. Neutral endopeptidase inhibits neuropeptide-mediated transactivation of the insulin-like growth factor receptor-Akt cell survival pathway. Cancer Res. 2001 Apr 15;61(8):3294-8.

Sun Z, Zhang J, Zhang J, Chen C, Du Q, Chang L, Cao C, Zheng M, Garcia-Barrio MT, Chen YE, Xiao RP, Mao J, Zhu X. Rad GTPase induces cardiomyocyte apoptosis through the activation of p38 mitogen-activated protein kinase. Biochem Biophys Res Commun. 2011 May 27;409(1):52-7.

Turner PR, Mefford S, Christakos S, Nissenson RA. Apoptosis mediated by activation of the G protein-coupled receptor for parathyroid hormone (PTH)/PTH-related protein (PTHrP). Mol Endocrinol. 2000 Feb;14(2):241-54.

Ueno M, Itoh M, Kong L, Sugihara K, Asano M, Takakura N. PSF1 is essential for early embryogenesis in mice. Mol Cell Biol. 2005 Dec;25(23):10528-32.

Vaidyanathan G, Cismowski MJ, Wang G, Vincent TS, Brown KD, Lanier SM. The Ras-related protein AGS1/RASD1 suppresses cell growth. Oncogene. 2004 Jul 29;23(34):5858-63

Wang H, Maechler P, Antinozzi PA, Herrero L, Hagenfeldt-Johansson KA, Bjorklund A, Wollheim CB. The transcription factor SREBP-1c is instrumental in the development of beta-cell dysfunction. J Biol Chem. 2003 May 9;278(19):16622-9.

Welch C, Santra MK, El-Assaad W, Zhu X, Huber WE, Keys RA, Teodoro JG, Green MR. Identification of a protein, G0S2, that lacks Bcl-2 homology domains and interacts with and antagonizes Bcl-2. Cancer Res. 2009 Sep 1;69(17):6782-9. Epub 2009 Aug 25.

Woo IS, Eun SY, Jang HS, Kang ES, Kim GH, Kim HJ, Lee JH, Chang KC, Kim JH, Han CW, Seo HG. Identification of ADP-ribosylation factor 4 as a suppressor of N-(4-hydroxyphenyl)retinamide-induced cell death. Cancer Lett. 2009 Apr 8;276(1):53-60. Epub 2008 Nov 28.

Wu L, Ma CA, Zhao Y, Jain A. Aurora B interacts with NIR-p53, leading to p53 phosphorylation in its DNA-binding domain and subsequent functional suppression. J Biol Chem. 2011 Jan 21;286(3):2236-44.

Wu M, Xu LG, Zhai Z, Shu HB. SINK is a p65-interacting negative regulator of NF-kappaB-dependent transcription. J Biol Chem. 2003 Jul 18;278(29):27072-9. Epub 2003 May 7.

Ye J, Wang S, Leonard SS, Sun Y, Butterworth L, Antonini J, Ding M, Rojanasakul Y, Vallyathan V, Castranova V, Shi X. Role of reactive oxygen species and p53 in chromium(VI)-induced apoptosis. J Biol Chem. 1999 Dec 3;274(49):34974-80.

Zhang DS, Liu ZY, Li YJ, Sun ZL. NQO1 involves in the imine bond reduction of sanguinarine and recombinant adeno-associated virus mediated NQO1 overexpression decreases sanguinarine-induced cytotoxicity in rat BRL cells. Toxicol Lett. 2014 Feb 10;225(1):119-29.

Zhang D, Zhang J, Jiang X, Li X, Wang Y, Ma J, Jiang H. Heparin-binding epidermal growth factor-like growth factor: a hepatic stellate cell proliferation inducer via ErbB receptors. J Gastroenterol Hepatol. 2014 Mar;29(3):623-32.

Zhao Y, Altman BJ, Coloff JL, Herman CE, Jacobs SR, Wieman HL, Wofford JA, Dimascio LN, Ilkayeva O, Kelekar A, Reya T, Rathmell JC. Glycogen synthase kinase 3alpha and 3beta mediate a glucose-sensitive antiapoptotic signaling pathway to stabilize Mcl-1. Mol Cell Biol. 2007 Jun;27(12):4328-39.
