# Supplementary material for: Transcriptome adaptation of the bovine mammary gland to diets rich in unsaturated fatty acids shows greater impact of linseed oil over safflower oil on gene expression and metabolic pathways
Source: BMC Genomics. 2016 Feb 9;17:104. doi: 10.1186/s12864-016-2423-x (PMC4748538; doi:10.1186/s12864-016-2423-x)
Supplement: Additional file 18: — The relationships between DE genes (day-14 vs day+28) in the network of genes implicated in lipid metabolism/small molecule biochemistry/molecular transport in (A) linseed oil and (B) safflower oil treatments. (PDF 226 kb) [file 12864_2016_2423_MOESM18_ESM.pdf]

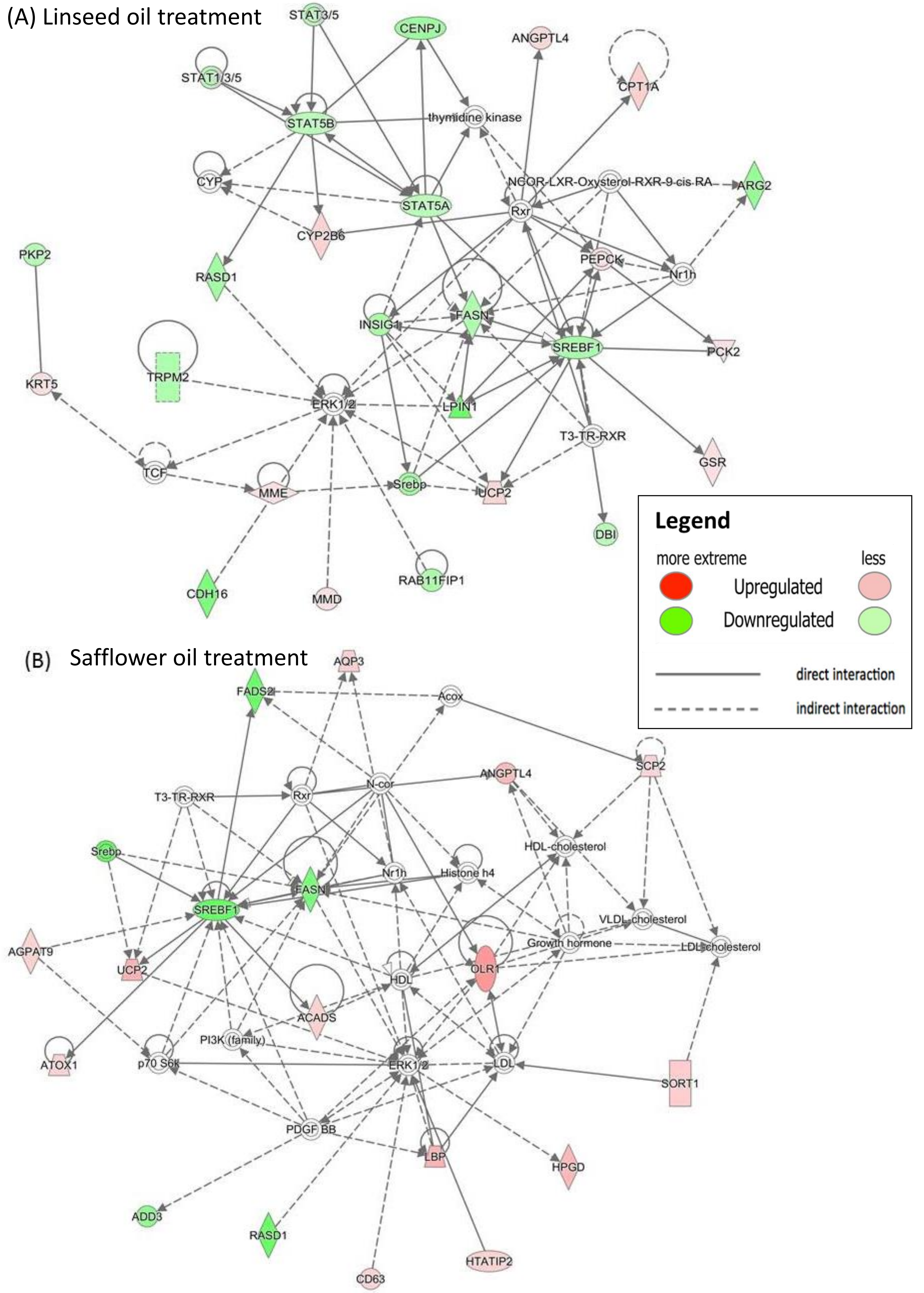

Additional file 18: The relationships between differentially expressed genes (day-14 vs day+28) in the network of genes implicated in lipid metabolism/small molecule biochemistry/molecular transport in (A) linseed oil and (B) safflower oil treatments
