# Supplementary material for: Transcriptome adaptation of the bovine mammary gland to diets rich in unsaturated fatty acids shows greater impact of linseed oil over safflower oil on gene expression and metabolic pathways
Source: BMC Genomics. 2016 Feb 9;17:104. doi: 10.1186/s12864-016-2423-x (PMC4748538; doi:10.1186/s12864-016-2423-x)
Supplement: Additional file 8: — Principal component analysis of the gene expression of samples from cows whose diets were supplemented with 5 % linseed oil or 5 % safflower oil (on dry matter bases). Neither component 1 and 2 (A), or 2 and 3 (B) could separate the samples according to treatments. Samples were widely dispersed while those from the same animal tended to group together, hinting at wide inter-animal variations. (PDF 178 kb) [file 12864_2016_2423_MOESM8_ESM.pdf]

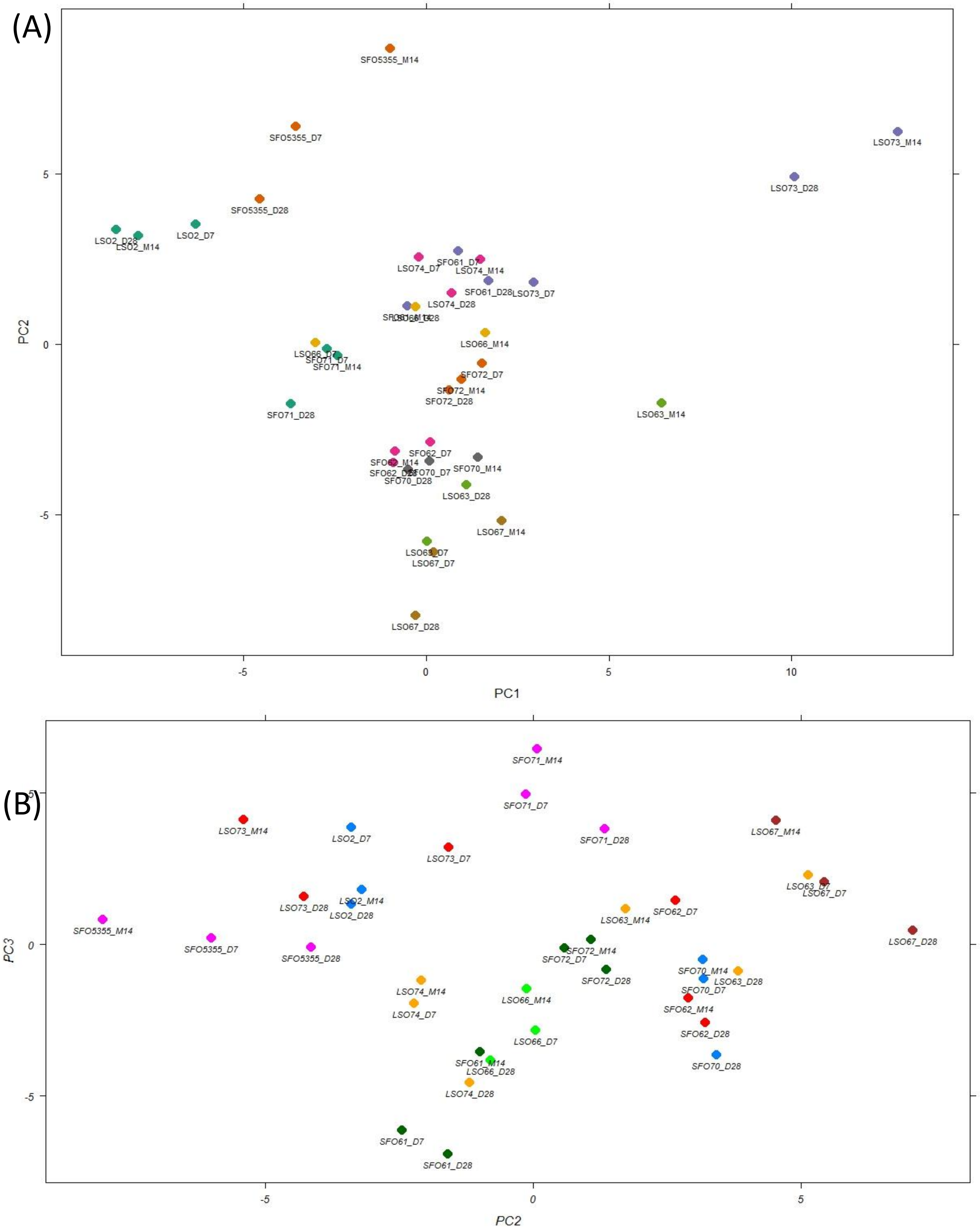

Additional file 8: Principal component analysis of the gene expression of samples from cows whose diets were supplemented with 5% linseed oil or 5% safflower oil (on dry matter bases). Neither component 1 and 2 (A), or 2 and 3 (B) could separate the samples according to treatments. Samples were widely dispersed while those from the same animal tended to group together, hinting at wide inter-animal variations.
